# Modelling the pyrenoid-based CO_2_-concentrating mechanism provides insights into its operating principles and a roadmap for its engineering into crops

**DOI:** 10.1038/s41477-022-01153-7

**Published:** 2022-05-19

**Authors:** Chenyi Fei, Alexandra T. Wilson, Niall M. Mangan, Ned S. Wingreen, Martin C. Jonikas

**Affiliations:** 1 Department of Molecular Biology, Princeton University, Princeton, NJ USA; 2 Lewis-Sigler Institute for Integrative Genomics, Princeton University, Princeton, NJ USA; 3 Department of Biology, Massachusetts Institute of Technology, Cambridge, MA USA; 4 Department of Engineering Sciences and Applied Mathematics, Northwestern University, Evanston, IL USA; 5 Howard Hughes Medical Institute, Princeton University, Princeton, NJ USA

**Keywords:** Plant biotechnology, Biological models

## Abstract

Many eukaryotic photosynthetic organisms enhance their carbon uptake by supplying concentrated CO_2_ to the CO_2_-fixing enzyme Rubisco in an organelle called the pyrenoid. Ongoing efforts seek to engineer this pyrenoid-based CO_2_-concentrating mechanism (PCCM) into crops to increase yields. Here we develop a computational model for a PCCM on the basis of the postulated mechanism in the green alga *Chlamydomonas reinhardtii*. Our model recapitulates all *Chlamydomonas* PCCM-deficient mutant phenotypes and yields general biophysical principles underlying the PCCM. We show that an effective and energetically efficient PCCM requires a physical barrier to reduce pyrenoid CO_2_ leakage, as well as proper enzyme localization to reduce futile cycling between CO_2_ and HCO_3_^−^. Importantly, our model demonstrates the feasibility of a purely passive CO_2_ uptake strategy at air-level CO_2_, while active HCO_3_^−^ uptake proves advantageous at lower CO_2_ levels. We propose a four-step engineering path to increase the rate of CO_2_ fixation in the plant chloroplast up to threefold at a theoretical cost of only 1.3 ATP per CO_2_ fixed, thereby offering a framework to guide the engineering of a PCCM into land plants.

## Abstract (web summary)

To enhance CO_2_ fixation, algae concentrate CO_2_ in an organelle called the pyrenoid. A biophysical model provides systematic analysis of the mechanism and determines the minimal steps for its engineering into crops to enhance yields.

## Main

The CO_2_-fixing enzyme Rubisco mediates the entry of roughly 10^14^ kilograms of carbon into the biosphere each year^1–3^. However, in many plants Rubisco fixes CO_2_ at less than one-third of its maximum rate under atmospheric levels of CO_2_ (Supplementary Fig. 1)^4–6^, which limits the growth of crops such as rice and wheat^7^. To overcome this limitation, many photosynthetic organisms, including C_4_ plants^8,9^, crassulacean acid metabolism (CAM) plants^10^, algae^11,12^ and cyanobacteria^13^, enhance Rubisco’s CO_2_ fixation rate by supplying it with concentrated CO_2_^14,15^. In algae, such a CO_2_-concentrating mechanism occurs within a phase-separated organelle called the pyrenoid^16–19^. Pyrenoid-based CO_2_-concentrating mechanisms (PCCMs) mediate approximately one-third of global CO_2_ fixation^16^.

While previous works have identified essential molecular components for the PCCM^16,20–29^, key operating principles of this mechanism remain poorly understood due to a lack of quantitative and systematic analysis. At the same time, there is growing interest in engineering a PCCM into C_3_ crops to improve yields and nitrogen- and water-use efficiency^30,31^. Key questions are: (1) What is the minimal set of components necessary to achieve a functional PCCM? (2) What is the energetic cost of operating a minimal PCCM?

**Fig. 1:**
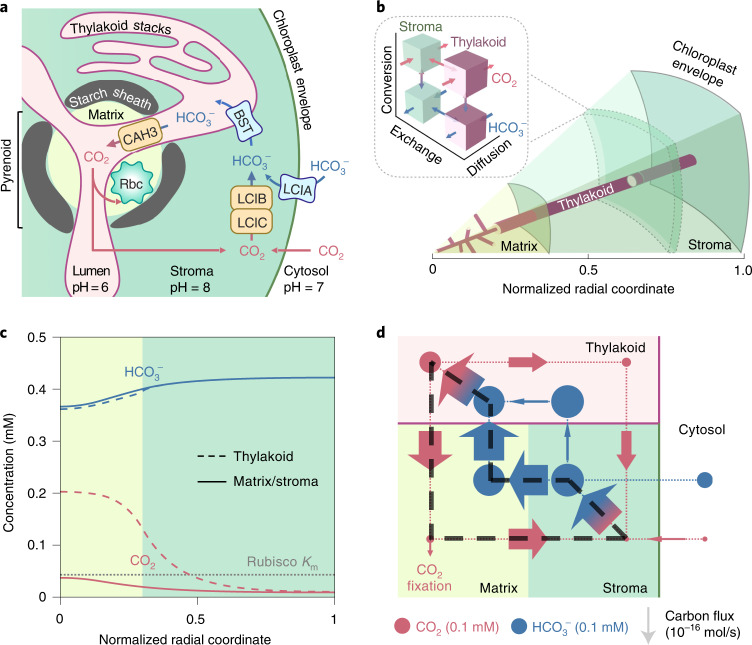
A multicompartment reaction-diffusion model describes the *Chlamydomonas* PCCM. **a**, Cartoon of a *Chlamydomonas* chloroplast with known PCCM components. HCO_3_^−^ is transported across the chloroplast membrane by LCIA and across the thylakoid membranes by BST1–3 (referred to as BST henceforth for simplicity). In the acidic thylakoid lumen, a carbonic anhydrase CAH3 converts HCO_3_^−^ into CO_2_, which diffuses into the pyrenoid matrix where the CO_2_-fixing enzyme Rubisco (Rbc) is localized. CO_2_ leakage out of the matrix and the chloroplast can be impeded by potential diffusion barriers—a starch sheath and stacks of thylakoids—and by conversion to HCO_3_^−^ by a CO_2_-recapturing complex LCIB/LCIC (referred to as LCIB henceforth for simplicity) in the basic chloroplast stroma. **b**, A schematic of the modelled PCCM, which considers intracompartment diffusion and intercompartment exchange of CO_2_ and HCO_3_^−^, as well as their interconversion, as indicated in the inset. Colour code as in **a**. The model is spherically symmetric and consists of a central pyrenoid matrix surrounded by a stroma. Thylakoids run through the matrix and stroma; their volume and surface area correspond to a reticulated network at the centre of the matrix extended by cylinders running radially outward. **c**, Concentration profiles of CO_2_ and HCO_3_^−^ in the thylakoid (dashed curves) and in the matrix/stroma (solid curves) for the baseline PCCM model that lacks LCIA activity and diffusion barriers. Dotted grey line indicates the effective Rubisco *K*_m_ for CO_2_ (Methods). Colour code as in **a**. **d**, Net fluxes of inorganic carbon between the indicated compartments. The width of arrows is proportional to flux; the area of circles is proportional to the average molecular concentration in the corresponding regions. The black dashed loop denotes the major futile cycle of inorganic carbon in the chloroplast. Colour code as in **a**. For **c** and **d**, LCIA^C^-mediated chloroplast membrane permeability to HCO_3_^−^ $\kappa _{{{{\mathrm{chlor}}}}}^{H^ - }$ = 10^−8^ m s^−1^, BST-mediated thylakoid membrane permeability to HCO_3_^−^ $\kappa _{{{{\mathrm{thy}}}}}^{H^ - }$ = 10^−2^ m s^−1^, LCIB rate *V*_LCIB_ = 10^3^ s^−1^ and CAH3 rate *V*_CAH3_ = 10^4^ s^−1^ (Methods). Other model parameters are estimated from experiments (Supplementary Table 2).

To advance our understanding of the PCCM, we develop a reaction-diffusion model on the basis of the postulated mechanism in the green alga *Chlamydomonas reinhardtii* (*Chlamydomonas* hereafter; Fig. 1a)^31–33^: Briefly, external inorganic carbon (Ci: CO_2_ and HCO_3_^−^) is transported across the plasma membrane by transporters LCI1 (Cre03.g162800) and HLA3 (Cre02.g097800)^23,24,34^. Cytosolic Ci becomes concentrated in the chloroplast stroma in the form of HCO_3_^−^, either via conversion of CO_2_ to HCO_3_^−^ by the putative stromal carbonic anhydrase LCIB/LCIC (Cre10.g452800/Cre06.g307500) complex (LCIB hereafter)^22,35,36^ or via direct transport across the chloroplast membrane by the poorly characterized HCO_3_^−^ transporter LCIA (Cre06.g309000)^24,37^. It is currently not known whether LCIA is a passive channel or a pump; therefore, in the model we first consider it as a passive channel (denoted by LCIA^C^) and later consider it as an active pump (denoted by LCIA^P^). Once in the stroma, HCO_3_^−^ travels via the putative HCO_3_^−^ channels BST1–3 (Cre16.g662600, Cre16.g663400 and Cre16.g663450)^25^ into the thylakoid lumen, and diffuses via membrane tubules into the pyrenoid where the carbonic anhydrase CAH3 (Cre09.g415700)^38–40^ converts HCO_3_^−^ into CO_2_. This CO_2_ diffuses from the thylakoid tubule lumen into the pyrenoid matrix, where Rubisco catalyses fixation. Supplementary Table 1 summarizes the acronyms of key proteins in the *Chlamydomonas* PCCM.

We model the above enzymatic activities and Ci transport in a spherical chloroplast. We assume that carbonic anhydrases catalyse the bidirectional interconversion of CO_2_ and HCO_3_^−^, producing a net flux in one direction where the two species are out of equilibrium. We consider three chloroplast compartments at constant pH values: a spherical pyrenoid matrix (pH 8, ref. ^41^) in the centre, a surrounding stroma (pH 8, ref. ^41,42^), and thylakoids (luminal pH 6, ref. ^43^) traversing both the matrix and stroma (Fig. 1b and Supplementary Fig. 2). The flux balance of intracompartment reaction and diffusion and intercompartment exchange sets the steady-state concentration profiles of Ci species in all compartments (Methods). To account for the effect of Ci transport across the cell membrane, we simulate a broad range of surrounding cytosolic Ci pools from which the chloroplast can uptake Ci. We characterize the performance of the modelled PCCM with two metrics: (1) its efficacy, quantified by the computed CO_2_ fixation flux normalized by the maximum possible flux through Rubisco; and (2) its efficiency, quantified by the ATP cost per CO_2_ fixed (Methods).

## Results

### A baseline PCCM driven by intercompartmental pH differences

To identify the minimal components of a functional PCCM, we build a baseline model (Fig. 1c,d), with the carbonic anhydrase LCIB diffuse throughout the stroma, BST channels for HCO_3_^−^ uniformly distributed across the thylakoid membranes, the carbonic anhydrase CAH3 localized to the thylakoid lumen within the pyrenoid, and Rubisco condensed within the pyrenoid matrix. This model lacks the HCO_3_^−^ transporter LCIA and potential diffusion barriers to Ci. We first analyse modelled PCCM performance under air-level CO_2_ (10 μM cytosolic); lower CO_2_ conditions are discussed in later sections.

CO_2_ diffusing into the chloroplast is converted to HCO_3_^−^ in the high-pH stroma where the equilibrium CO_2_:HCO_3_^−^ ratio is 1:80 (Methods). Since passive diffusion of HCO_3_^−^ across the chloroplast envelope is very slow, this concentrated HCO_3_^−^ becomes trapped in the stroma. The BST channels equilibrate HCO_3_^−^ across the thylakoid membrane, so HCO_3_^−^ also reaches a high concentration in the thylakoid lumen (Fig. 1c). The low pH in the thylakoid lumen favours a roughly equal equilibrium partition between CO_2_ and HCO_3_^−^; however, HCO_3_^−^ is not brought into equilibrium with CO_2_ immediately upon entering the thylakoid outside the pyrenoid, since no carbonic anhydrase (CA) is present there. Instead, HCO_3_^−^ diffuses within the thylakoid lumen towards the pyrenoid, where CAH3 localized within the pyrenoid radius rapidly converts HCO_3_^−^ back to CO_2_ (Fig. 1d). This CO_2_ can diffuse across the thylakoid membrane into the pyrenoid matrix. This baseline model, driven solely by intercompartmental pH differences, achieves a pyrenoidal CO_2_ concentration approximately 2.5 times that found in a model with no PCCM.

### The baseline PCCM suffers from pyrenoid CO_2_ leakage

**Fig. 2:**
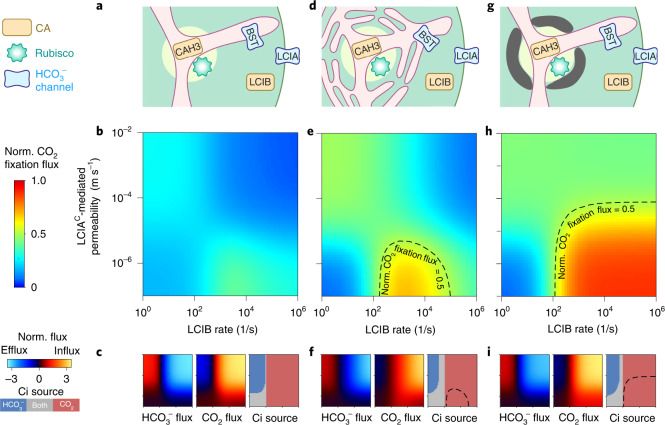
Barriers to CO_2_ diffusion out of the pyrenoid matrix enable an effective PCCM driven only by intercompartmental pH differences. **a**–**i**, A model with no barrier to CO_2_ diffusion out of the pyrenoid matrix (**a**–**c**) is compared to a model with thylakoid stacks slowing inorganic carbon diffusion in the stroma (**d**–**f**) and a model with an impermeable starch sheath (**g**–**i**) under air-level CO_2_ (10 µM cytosolic). **a**,**d**,**g**, Schematics of the modelled chloroplast. **b**,**e**,**h**, Heatmaps of normalized CO_2_ fixation flux, defined as the ratio of the total Rubisco carboxylation flux to its maximum if Rubisco were saturated, at varying LCIA^C^-mediated chloroplast membrane permeabilities to HCO_3_^−^ and varying LCIB rates. The BST-mediated thylakoid membrane permeability to HCO_3_^−^ is the same as in Fig. 1c,d. For **e** and **h**, dashed black curves indicate a normalized CO_2_ fixation flux of 0.5. **c**,**f**,**i**, Overall fluxes of HCO_3_^−^ (left) and CO_2_ (middle) into the chloroplast, normalized by the maximum CO_2_ fixation flux if Rubisco were saturated, at varying LCIA^C^-mediated chloroplast membrane permeabilities to HCO_3_^−^ and varying LCIB rates. Negative values denote efflux out of the chloroplast. The inorganic carbon (Ci) species with a positive influx is defined as the Ci source (right). Axes are the same as in **b**, **e** and **h**.

The substantial CO_2_ leakage out of the matrix in the baseline model (Fig. 1d) is in part due to the relatively slow kinetics of Rubisco. During the average time required for a CO_2_ molecule to be fixed by Rubisco in the pyrenoid, that CO_2_ molecule can typically diffuse a distance larger than the pyrenoid radius (Supplementary Note I). Therefore, most of the CO_2_ molecules entering the pyrenoid matrix will leave without being fixed by Rubisco (Supplementary Fig. 3). One might think that adding LCIA^C^ as a passive channel to enhance HCO_3_^−^ diffusion into the chloroplast could overcome this deficit (Fig. 2a). However, even with the addition of LCIA^C^ to our baseline PCCM model, no combination of enzymatic activities and channel transport rates achieves an effective PCCM, that is, more than half-saturation of Rubisco with CO_2_ (Fig. 2b and Supplementary Fig. 4). Thus, the pH-driven PCCM cannot operate effectively without a diffusion barrier.

### Barriers to pyrenoidal CO_2_ leakage enable a pH-driven PCCM

To operate a more effective PCCM, the cell must reduce CO_2_ leakage from the pyrenoid matrix. A barrier to CO_2_ diffusion has been regarded as essential for various CO_2_-concentrating mechanisms^44–47^. Although the matrix is densely packed with Rubisco, our analysis suggests that the slowed diffusion of CO_2_ in the pyrenoid matrix due to volume occupied by Rubisco can only account for a 10% decrease in CO_2_ leakage (Supplementary Note VI.C). Thus, we consider alternative barriers in our model.

We speculate that thylakoid membrane sheets and the pyrenoid starch sheath could serve as effective barriers to decrease leakage of CO_2_ from the matrix. Thylakoid membrane sheets could serve as effective barriers to CO_2_ diffusion because molecules in the stroma must diffuse between and through the interdigitated membranes^45^. Indeed, our first-principle simulations suggest that the thylakoid stacks, modelled with realistic geometry^48^, effectively slow the diffusion of Ci in the stroma (Supplementary Fig. 5). Evidence on the role of the starch sheath in the PCCM is limited and mixed. While early work suggested that a starchless *Chlamydomonas* mutant had normal PCCM performance in air^49^, the phenotype was not compared to the appropriate parental strain. A more recent study found that a mutant (*sta2-1*) with a thinner starch sheath than wild-type strains displays decreased PCCM efficacy at very low CO_2_^50^. On the basis of the latter work, we hypothesize that the starch sheath that surrounds the matrix may act as a barrier to CO_2_ diffusion. Since the starch sheath consists of many lamellae of crystalline amylopectin^51–53^, we model it as an essentially impermeable barrier equivalent to 10 lipid bilayers; in its presence, most CO_2_ leakage out of the matrix occurs through the thylakoid tubules (Supplementary Fig. 6).

We next test whether the above two realistic diffusion barriers allow for an effective pH-driven PCCM. Adding either thylakoid stacks or a starch sheath to the baseline PCCM model above drastically reduces CO_2_ leakage from the matrix to the stroma (Supplementary Fig. 7). The resulting PCCM is highly effective under air-level CO_2_ (10 μM cytosolic) conditions: pyrenoidal CO_2_ concentrations are raised above the effective half-saturation constant *K*_m_ of Rubisco (Methods) using only the intercompartmental pH differential and passive Ci uptake (Fig. 2e,h). PCCM performance with both barriers present closely resembles the impermeable starch sheath case (Supplementary Fig. 8); for simplicity, we omit such a combined model from further discussion.

### Optimal passive Ci uptake uses cytosolic CO_2_, not HCO_3_^−^

In addition to the requirement for a diffusion barrier, the efficacy of the pH-driven PCCM depends on the LCIB rate and the LCIA^C^-mediated chloroplast membrane permeability to HCO_3_^−^ (Fig. 2b,e,h). Depending on LCIB activity, our model suggests two distinct strategies to passively uptake Ci. If LCIB activity is low, CO_2_ fixation flux increases with higher LCIA^C^-mediated permeability to HCO_3_^−^, which facilitates the diffusion of cytosolic HCO_3_^−^ into the stroma (Fig. 2c,f,i). In contrast, if LCIB activity is high, CO_2_ fixation flux is maximized when LCIA^C^-mediated permeability is low; in this case, a diffusive influx of CO_2_ into the chloroplast is rapidly converted by LCIB into HCO_3_^−^, which becomes trapped and concentrated in the chloroplast. Under this scenario, permeability of the chloroplast membrane to HCO_3_^−^ due to LCIA^C^ is detrimental, since it allows HCO_3_^−^ converted by LCIB in the stroma to diffuse back out to the cytosol (Fig. 2c,f,i).

Interestingly, the highest CO_2_ fixation flux is achieved by passive CO_2_ uptake mediated by the carbonic anhydrase activity of LCIB, not by passive HCO_3_^−^ uptake via LCIA^C^ channels (Fig. 2), even though HCO_3_^−^ is more abundant than CO_2_ in the cytosol. The key consideration is that the stroma (at pH 8) is more basic than the cytosol (at pH 7.1, ref. ^54^), which allows LCIB to equilibrate passively acquired CO_2_ with HCO_3_^−^ to create an even higher HCO_3_^−^ concentration in the stroma than in the cytosol.

### The PCCM requires active Ci uptake under very low CO_2_

**Fig. 3:**
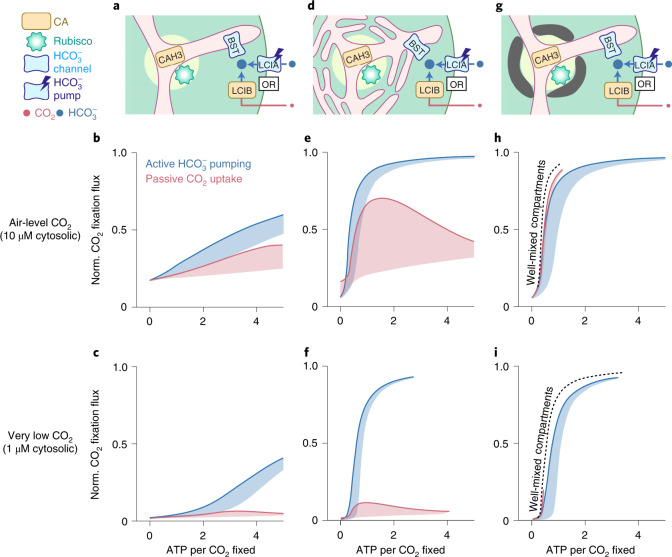
Feasible inorganic carbon uptake strategies for the chloroplast depend on the environmental level of CO_2_. **a**–**i**, Results are shown for a model with no barrier to CO_2_ diffusion out of the pyrenoid matrix (**a**–**c**), a model with thylakoid stacks serving as diffusion barriers (**d**–**f**) and a model with an impermeable starch sheath (**g**–**i**). **a**,**d**,**g**, Schematics of the modelled chloroplast employing LCIB for passive CO_2_ uptake (red), or employing active LCIA^P^-mediated HCO_3_^−^ pumping across the chloroplast envelope and no LCIB activity (blue). PCCM performance under air-level CO_2_ (10 µM cytosolic) (**b**,**e**,**h**) and under very low CO_2_ (1 µM cytosolic) (**c**,**f**,**i**) are shown, as measured by normalized CO_2_ fixation flux versus ATP spent per CO_2_ fixed, for the two inorganic carbon uptake strategies in **a**, **d** and **g**. Solid curves indicate the minimum energy cost necessary to achieve a certain normalized CO_2_ fixation flux. Shaded regions represent the range of possible performances found by varying HCO_3_^−^ transport rates and LCIB rates. Colour code as in **a**. In **h** and **i,** dashed black curves indicate the optimal PCCM performance of a simplified model that assumes fast intracompartmental diffusion, fast HCO_3_^−^ diffusion across the thylakoid membranes, and fast equilibrium between CO_2_ and HCO_3_^−^ catalysed by CAH3 in the thylakoid tubules inside the pyrenoid (Methods).

While the passive CO_2_ uptake strategy can power the pH-driven PCCM under air-level CO_2_ (10 μM cytosolic), its Ci uptake rate is ultimately limited by the diffusion of CO_2_ across the chloroplast envelope. Indeed, our simulations show that under very low CO_2_ conditions (1 μM cytosolic)^55^, a chloroplast using the passive CO_2_ uptake strategy can only achieve at most 20% of its maximum CO_2_ fixation flux, even in the presence of barriers to Ci diffusion (Fig. 3). Since passive HCO_3_^−^ uptake cannot concentrate more Ci than passive CO_2_ uptake (Fig. 2), we hypothesize that active Ci transport is required for an effective PCCM at very low CO_2_. To test this idea, we consider a model employing active LCIA HCO_3_^−^ pumps (LCIA^P^) without LCIB activity (Fig. 3a,d,g). We find that, indeed, HCO_3_^−^ pumping enables saturating CO_2_ fixation flux under very low CO_2_ conditions (Fig. 3 and Supplementary Fig. 12).

### Both passive and active Ci uptake can have low energy cost

According to our model, both passive CO_2_ uptake and active HCO_3_^−^ pumping can support an effective PCCM under air-level CO_2_. However, the latter directly consumes energy to achieve non-reversible transport. What is the total energy cost of a PCCM that employs active HCO_3_^−^ uptake, and how does this cost compare to that of the passive CO_2_ uptake strategy? To answer these questions, we used a nonequilibrium thermodynamics framework to compute the energy cost of different Ci uptake strategies (Supplementary Note II and Fig. 13)^56^. First, a PCCM without diffusion barriers is energetically expensive regardless of the Ci uptake strategies employed (Fig. 3a–c). Second, in the presence of diffusion barriers, we find that the passive CO_2_ uptake strategy can achieve similar energy efficiency (~1 ATP cost per CO_2_ fixed) to the active HCO_3_^−^ uptake strategy (Fig. 3d–i). Thus, both strategies can achieve high PCCM performance at air-level CO_2_; however, active HCO_3_^−^ uptake is necessary to achieve high efficacy under lower CO_2_.

### The PCCM depends on cytosolic Ci and its chloroplast uptake

How does Ci transport across the cell’s plasma membrane impact the feasible Ci uptake strategies at the chloroplast level? To explore this question in our chloroplast-scale model, we assess PCCM performance under a broad range of cytosolic CO_2_ and HCO_3_^−^ concentrations (Supplementary Fig. 15). Unsurprisingly, we find that the performance of a particular chloroplast Ci uptake strategy increases with the cytosolic level of its target Ci species. Thus, it is important to replenish cytosolic Ci species taken up by the chloroplast. Moreover, regardless of the makeup of the cytosolic Ci pool, a chloroplast lacking both passive CO_2_ uptake and active HCO_3_^−^ uptake fails to achieve high PCCM efficacy, unless the cytosolic CO_2_ concentration is 100 μM or higher. Creating such a pool would presumably result in substantial CO_2_ leakage across the plasma membrane and thus high energy cost. Therefore, effective mechanisms for Ci uptake from the external environment to the cytosol and from cytosol to the chloroplast are both essential for high PCCM performance.

### Carbonic anhydrase localization alters modelled Ci fluxes

**Fig. 4:**
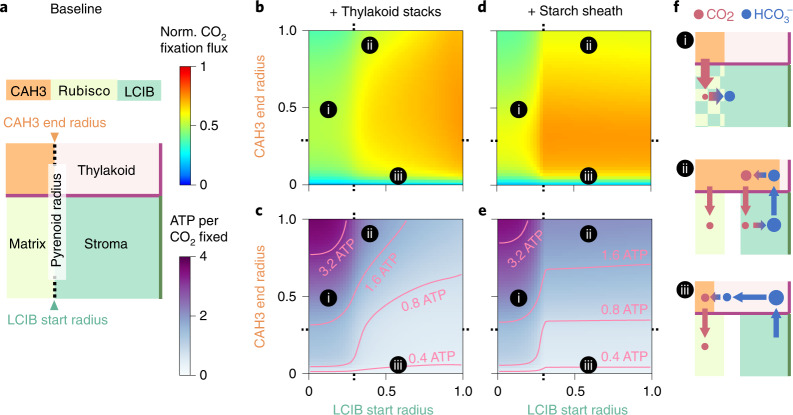
Proper localization of carbonic anhydrases enhances PCCM performance. **a**, Schematics of varying localization of carbonic anhydrases. The CAH3 domain starts in the centre of the intrapyrenoid tubules (radius *r* = 0) and the LCIB domain ends at the chloroplast envelope. Colour code as in Fig. 1d. Orange denotes region occupied by CAH3. **b**–**e**, CAH3 end radius and LCIB start radius are varied in a modelled chloroplast employing the passive CO_2_ uptake strategy under air-level CO_2_, with thylakoid stacks slowing inorganic carbon diffusion in the stroma (**b**,**c**) or with an impermeable starch sheath (**d**,**e**). Normalized CO_2_ fixation flux (**b**,**d**) and ATP spent per CO_2_ fixed (**c**,**e**) when the localizations of carbonic anhydrases are varied. **f**, Schematics of inorganic carbon fluxes for the localization patterns (i–iii) indicated in **b**–**e**. Colour code as in **a** and Fig. 1d. Dotted ticks in **b**–**e** denote pyrenoid radius as in **a**. Simulation parameters are the same as in Fig. 1c,d.

So far, we have only considered the carbonic anhydrase localization patterns that are thought to exist in *Chlamydomonas* under air-level CO_2_^40,57^. To assess the benefits of such localization, we vary the localization of CAH3 and LCIB while maintaining the total number of molecules of each carbonic anhydrase (Fig. 4a). We find that ectopic carbonic anhydrase localization compromises PCCM performance. First, LCIB mislocalized to the basic pyrenoid matrix (pH 8) converts Rubisco’s substrate CO_2_ into HCO_3_^−^, and hence decreases CO_2_ fixation (Fig. 4b–f, region i). Second, when CAH3 is distributed in the thylakoids outside the pyrenoid, CO_2_ molecules produced by this CAH3 can diffuse directly into the stroma, making them less likely to be concentrated in the pyrenoid and thus decreasing the efficacy of the PCCM (Fig. 4b–f, region ii, and Supplementary Fig. 16). Moreover, CAH3 mislocalization outside the pyrenoid decreases PCCM efficiency as it leads to increased futile cycling of Ci between the stroma and thylakoid, increasing the energetic cost required to maintain the intercompartmental pH differences. Finally, concentrating CAH3 to a small region of thylakoid lumen in the centre of the pyrenoid increases the distance over which HCO_3_^−^ needs to diffuse before it is converted to CO_2_, thus lowering the CO_2_ production flux by CAH3 (Fig. 4b–f, region iii). All these results hold true both at air-level CO_2_ employing passive CO_2_ uptake (Fig. 4) and at very low CO_2_ employing active HCO_3_^−^ uptake (Supplementary Fig. 17). Thus, our model shows that proper carbonic anhydrase localization is crucial to overall PCCM performance.

### Effects of LCIB activity and localization at very low CO_2_

**Fig. 5:**
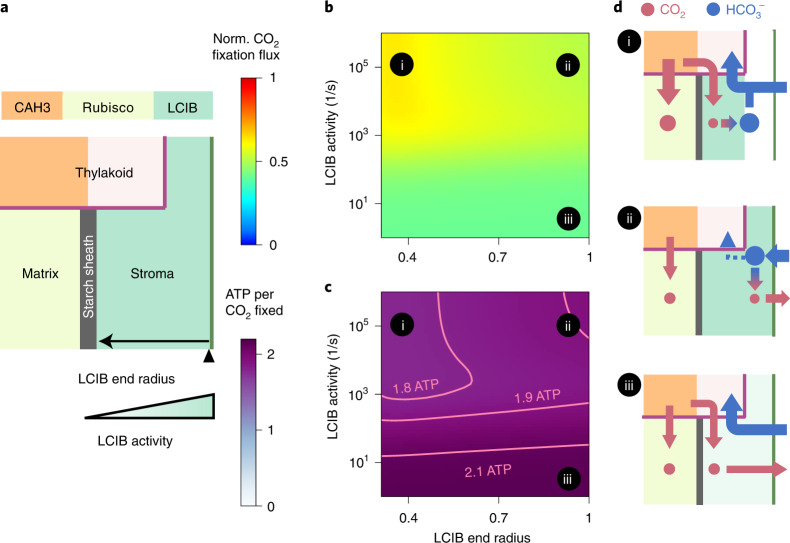
Localization of LCIB around the pyrenoid periphery reduces Ci leakage out of the chloroplast. **a**, Schematics of varying activity and end radius of LCIB in a modelled chloroplast employing an impermeable starch sheath and active HCO_3_^−^ pumping across the chloroplast envelope under very low CO_2_. Colour code as in Fig. 4a. The LCIB domain starts at the pyrenoid radius (0.3 on the *x* axis in **b** and **c**). **b**,**c**, Normalized CO_2_ fixation flux (**b**) and ATP spent per CO_2_ fixed (**c**) when the designated characteristics of LCIB are varied. **d**, Schematics of inorganic carbon fluxes for the LCIB states (i–iii) indicated in **b** and **c**. Colour code as in Fig. 4f. Simulation parameters as in Fig. 4. Active LCIA^P^-mediated HCO_3_^−^ pumping is described by the rate $\kappa _{{{{\mathrm{chlor}}}}}^{H^ - }$ = 10^−4^ m s^−1^ and the reversibility *γ* = 10^−4^. To show a notable variation in normalized CO_2_ fixation flux, a model with shortened thylakoid tubules is simulated (Methods). The qualitative results hold true independent of this specific choice.

When shifted from air levels to very low levels of CO_2_ (~1 μM dissolved), *Chlamydomonas* relocalizes LCIB from diffuse throughout the stroma to localized around the pyrenoid periphery^57^. To better understand the value of LCIB localization to the pyrenoid periphery under very low CO_2_, we vary both the end radius of stromal LCIB, which defines how far LCIB extends towards the chloroplast envelope, and the total number of LCIB molecules in a model employing a starch sheath barrier and active HCO_3_^−^ uptake (Fig. 5a). Our analysis shows that it is energetically wasteful to allow concentrated CO_2_ to leak out of the chloroplast (Supplementary Fig. 13). Consequently, LCIB relocalized near the starch sheath increases energy efficiency by recapturing CO_2_ molecules that diffuse out of the matrix and trapping them as HCO_3_^−^ in the chloroplast (Fig. 5b–c, region i). The energy cost is higher without any LCIB for CO_2_ recapture (Fig. 5b–c, region iii), or with diffuse stromal LCIB, which allows incoming HCO_3_^−^ to be converted into CO_2_ near the chloroplast membrane at which point it can leak back to the cytosol (Fig. 5b–c, region ii, and Supplementary Fig. 19). Our model thus suggests that under very low CO_2_ and in the presence of a strong CO_2_ diffusion barrier around the pyrenoid, localizing LCIB at the pyrenoid periphery allows for efficient Ci recycling, therefore enhancing PCCM performance.

### Intercompartmental pH differences are key to PCCM function

**Fig. 6:**
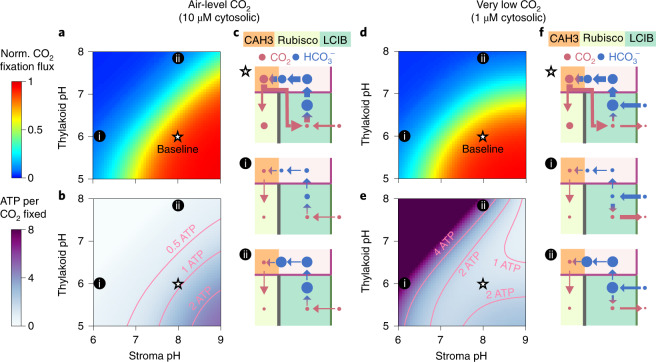
High PCCM performance requires low-pH thylakoids and a high-pH stroma. **a**–**f**, pH values of the thylakoid lumen and the stroma are varied in a modelled chloroplast with an impermeable starch sheath employing passive CO_2_ uptake under air-level CO_2_ (**a**–**c**) (10 μM cytosolic; parameters as in Fig. 4d,e) or active HCO_3_^−^ pumping under very low CO_2_ (**d**–**f**) (1 μM cytosolic, parameters as in Supplementary Fig. 17c,d). Normalized CO_2_ fixation flux (**a**,**d**) and ATP spent per CO_2_ fixed (**b**,**e**) as functions of the pH values in the two compartments are shown. **c**,**f**, Schematics of inorganic carbon pools and fluxes for the pH values indicated in **a**, **b**, **d** and **e**. White stars indicate the baseline pH values used in all other simulations.

To determine the impact of thylakoid lumen and stromal pH on PCCM function, we vary the pH values of the two compartments (Fig. 6 and Supplementary Fig. 20). We find that regardless of Ci uptake strategy, the modelled PCCM achieves high efficacy only when the thylakoid lumen is much more acidic than the stroma (Fig. 6a,d). Indeed, carbonic anhydrase activity in a low-pH stroma (Fig. 6, region i) or in a high-pH intrapyrenoid tubule lumen (Fig. 6, region ii) would lead to low concentrations of HCO_3_^−^ or CO_2_, respectively, in those compartments; both would be detrimental to the PCCM. Interestingly, variation in pH differentially influences the energy efficiency of the PCCM employing passive CO_2_ uptake (Fig. 6a–c) and the PCCM employing active HCO_3_^−^ pumping (Fig. 6d–f). Specifically, only the latter shows a dramatically increased energy cost when the stroma has a relatively low pH; in this case, most HCO_3_^−^ pumped into the stroma is converted to CO_2_ and is subsequently lost to the cytosol (Fig. 6e,f, regions i and ii). Thus, our results suggest that high PCCM performance requires maintenance of a high-pH stroma and a low-pH thylakoid lumen.

### The model recapitulates *Chlamydomonas* PCCM mutant phenotypes

We next explore whether our model can account for the phenotypes of known *Chlamydomonas* PCCM-deficient mutants. We select model parameters to best represent the effect of each mutation, assuming that the *Chlamydomonas* PCCM switches from passive CO_2_ uptake under air-level CO_2_ to active HCO_3_^−^ uptake under very low CO_2_ (Supplementary Figs. 23 and 24). Our simulation results show semi-quantitative agreement with experimental results for all published mutants (Supplementary Table 5) and provide mechanistic explanations for all recorded phenotypes. For example, our model captures that the *lcib* mutant fails to grow in air, presumably due to a defect in passive CO_2_ uptake. This phenotype implies that *Chlamydomonas* does not pump HCO_3_^−^ into the chloroplast under air-level CO_2_ because a modelled *lcib* mutant employing HCO_3_^−^ pumping has a PCCM effective enough to drive growth in air. Notably, the *lcib* mutant recovers growth under very low CO_2_, which we attribute to the activation of an HCO_3_^−^ uptake system under this condition^22,57,58^. Indeed, knockdown of the gene encoding the LCIA HCO_3_^−^ transporters in the *lcib* mutant background results in a dramatic decrease in CO_2_ fixation and growth under very low CO_2_^57^.

More broadly, our model recapitulates phenotypes of *Chlamydomonas* mutants lacking the HCO_3_^−^ transporter HLA3 or the CO_2_ transporter LCI1 at the plasma membrane. Indeed, knockdown of the gene encoding HLA3 (simulated as a lower level of cytosolic HCO_3_^−^) leads to a dramatic decrease in PCCM efficacy under very low CO_2_, presumably due to reduced HCO_3_^−^ import into the cell and thus into the chloroplast^23,24^. In contrast, the *lci1* single mutant shows a moderate decrease in PCCM efficacy under air-level CO_2_, presumably due to a reduced CO_2_ influx into the cytosol and thus into the chloroplast, but no effect on the PCCM under very low CO_2_, presumably due to the activation of an active HCO_3_^−^ uptake system under this condition^34^.

Finally, our model captures the phenotypes of *Chlamydomonas* starch mutants, which survive under both air-level and very low CO_2_ conditions presumably because thylakoid stacks can effectively block CO_2_ leakage from the pyrenoid in the absence of a starch sheath. The existence of non-starch diffusion barriers, such as the thylakoid stacks, may also help explain why some other pyrenoid-containing algae do not have a starch sheath^59^.

### Various thylakoid architectures can support PCCM function

The analysis of Ci fluxes in our model supports the long-held view that the thylakoid tubules traversing the pyrenoid in *Chlamydomonas* can deliver stromal HCO_3_^−^ to the pyrenoid, where it can be converted to CO_2_ by CAH3^32,60^. However, is a *Chlamydomonas*-like thylakoid architecture necessary to a functional PCCM? Certainly, eukaryotic algae display a variety of thylakoid morphologies, such as multiple non-connecting parallel thylakoid stacks passing through the pyrenoid, a single disc of thylakoids bisecting the pyrenoid matrix, or thylakoid sheets surrounding but not traversing the pyrenoid^61–64^. Our calculations show that different thylakoid morphologies could in principle support the functioning of an effective PCCM, as long as HCO_3_^−^ can diffuse into the low-pH thylakoid lumen and the thylakoid carbonic anhydrase is localized to the pyrenoid-proximal lumen (Supplementary Fig. 25).

### An effective PCCM needs Ci uptake, transport and trapping

Our model identifies a minimal PCCM configuration sufficient to effectively concentrate CO_2_. Next, we ask: can alternative configurations of the same minimal elements achieve an effective PCCM? We restrict our focus to PCCMs employing passive Ci uptake strategies. We measured the efficacy and energy cost of 216 partial PCCM configurations in air, varying the presence and localization of Rubisco, thylakoid and stromal carbonic anhydrases, HCO_3_^−^ channels on the thylakoid membranes and the chloroplast envelope, and diffusion barriers (Supplementary Fig. 26).

**Fig. 7:**
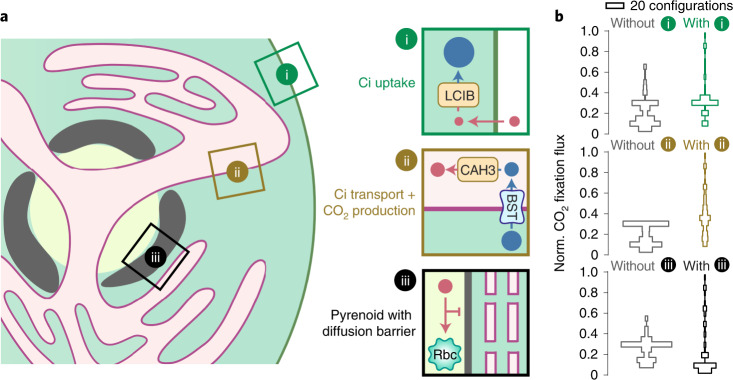
An effective PCCM is composed of three essential modules. **a**, Schematics of the three essential modules with designated functions (same style as in Fig. 1a). In *Chlamydomonas*, LCIB can be used for passive uptake of CO_2_, which is then trapped in the stroma as HCO_3_^−^ (module i); BST allows stromal HCO_3_^−^ to diffuse into the thylakoid lumen where CAH3 converts HCO_3_^−^ into CO_2_ (module ii); and a starch sheath and thylakoid stacks could act as diffusion barriers to slow CO_2_ escape out of the pyrenoid matrix (module iii). **b**, Histograms of normalized CO_2_ fixation flux for CCM configurations without (left, grey) or with (right, coloured) the respective module. We tested 216 CCM configurations by varying the presence and/or localization of enzymes, HCO_3_^−^ channels and diffusion barriers in the model (see Supplementary Fig. 26).

Our results summarize three central modules of an effective pH-driven PCCM (Fig. 7a): (i) a stromal carbonic anhydrase (LCIB) to convert passively acquired CO_2_ into HCO_3_^−^, (ii) a thylakoid membrane HCO_3_^−^ channel (BST) and a luminal carbonic anhydrase (CAH3) that together allow conversion of HCO_3_^−^ to CO_2_ near Rubisco, and (iii) a Rubisco condensate surrounded by diffusion barriers. We find that PCCM configurations lacking any one of these modules show a compromised ability to concentrate CO_2_ (Fig. 7b). The *Chlamydomonas*-like PCCM configuration is the only configuration possessing all three modules; thus, this configuration is not only sufficient but also necessary to achieve an effective PCCM using the considered minimal elements.

### Possible strategies for engineering a PCCM into land plants

Many land plants, including most crop plants, are thought to lack any form of CCM. Our analysis shows that a typical plant chloroplast configuration can only support ~30% of the maximum CO_2_ fixation flux through Rubisco (Supplementary Table 6). Engineering a PCCM into crops has emerged as a promising strategy to increase yields through enhanced CO_2_ fixation^30,31^. Despite early engineering advances including expressing individual PCCM components^65^ and reconstituting a pyrenoid matrix in plants^66^, the optimal order of engineering steps needed to establish an effective PCCM in a plant chloroplast remains unknown. Here we leverage our partial PCCM configurations to propose an engineering path that results in monotonic improvement of efficacy and avoids excessive energy costs.

**Fig. 8:**
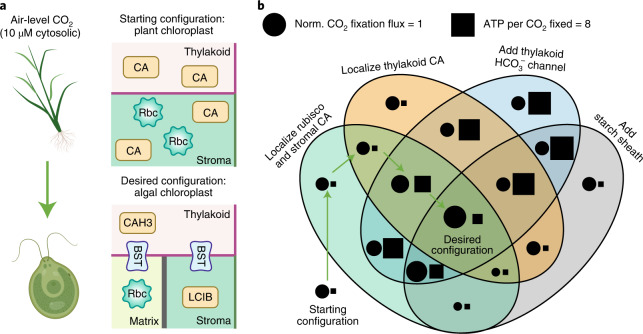
Proposed engineering path for installing a minimal PCCM into land plants. **a**, Top: schematics of the starting configuration representing a typical plant chloroplast that contains diffuse thylakoid carbonic anhydrase, diffuse stromal carbonic anhydrase, and diffuse Rubisco, and lacks HCO_3_^−^ transporters and diffusion barriers. Bottom: the desired configuration representing a *Chlamydomonas* chloroplast that employs the passive CO_2_ uptake strategy and a starch sheath (as in Fig. 2g). **b**, Venn diagram showing the normalized CO_2_ fixation flux (circle, area in proportion to magnitude) and ATP spent per CO_2_ fixed (square, area in proportion to magnitude) of various configurations after implementing the designated changes. Arrows denote the proposed sequential steps to transform the starting configuration into the desired configuration (see text). The starting configuration has a normalized CO_2_ fixation flux of 0.31 and negligible ATP cost. All costs below 0.25 ATP per CO_2_ fixed are represented by a square of the minimal size.

To the best of our knowledge, the plant chloroplast contains diffuse carbonic anhydrase and diffuse plant Rubisco in the stroma, and lacks HCO_3_^−^ channels and diffusion barriers^67^. We note that plant Rubisco has a lower *K*_m_ for CO_2_ than *Chlamydomonas* Rubisco; our engineering calculations account for this and employ values from plant Rubisco. Studies have also suggested that native plant carbonic anhydrases are diffuse in the thylakoid lumen^68^, which we therefore assume in our modelled plant chloroplast configuration (Fig. 8, starting configuration). This configuration contains only one of the three essential modules for an effective PCCM (Fig. 7a), that is, the passive CO_2_ uptake system.

After exploring all possible stepwise paths to install the remaining two modules to achieve the *Chlamydomonas*-like PCCM configuration (Fig. 8, desired configuration), we suggest the following path consisting of four minimal engineering steps (Fig. 8b, arrows). The first step is the localization of plant Rubisco to a pyrenoid matrix, which we assume would inherently exclude the plant stromal carbonic anhydrase, as the tight packing of Rubisco in the matrix appears to exclude protein complexes greater than ~80 kDa^26,69^. The second step is the localization of the thylakoid carbonic anhydrase to thylakoids that border or traverse the matrix. These first two steps do not yield notable changes to either the efficacy or the efficiency of the PCCM. The next step is to introduce HCO_3_^−^ channels to the thylakoid membranes, which increases the CO_2_ fixation flux to ~175% of that of the starting configuration. This step also increases the cost of the PCCM to around 4 ATPs per CO_2_ fixed. Such a high-cost step cannot be avoided, and all other possible paths with increasing efficacy at each step have more costly intermediate configurations (Fig. 8b and Supplementary Table 6). Importantly for engineering, the increased CO_2_ fixation flux resulting from this step would provide evidence that the installed channels are functional. The final step of the suggested path is to add a starch sheath to block CO_2_ leakage from the pyrenoid matrix, which triples the CO_2_ fixation flux compared with the starting configuration and reduces the cost to only 1.3 ATPs per CO_2_ fixed.

Selecting an alternative implementation order for the four minimal engineering steps leads to decreased performance of the PCCM in intermediate stages. For example, adding HCO_3_^−^ channels on the thylakoid membranes before the stromal and thylakoid carbonic anhydrases are localized (Fig. 8b, blue oval) leads to futile cycling generated by overlapping carbonic anhydrases (Fig. 4, region ii). Additionally, adding a starch sheath before HCO_3_^−^ channels are added to the thylakoids could decrease CO_2_ fixation (Fig. 8b, grey oval); without channels, HCO_3_^−^ cannot readily diffuse to the thylakoid carbonic anhydrase to produce CO_2_, and the starch sheath impedes diffusion of CO_2_ from the stroma to Rubisco. Thus, our suggested path avoids intermediate configurations with decreased efficacy or excessive energy cost.

## Discussion

To better understand the composition and function of a minimal PCCM, we developed a multicompartment reaction-diffusion model on the basis of the *Chlamydomonas* PCCM. The model not only accounts for all published *Chlamydomonas* PCCM mutants, but also lays the quantitative and biophysical groundwork for understanding the operating principles of a minimal PCCM. Systematic analysis of the model suggests that keys to an effective and energetically efficient PCCM are barriers preventing CO_2_ efflux from the pyrenoid matrix and carbonic anhydrase localizations preventing futile Ci fluxes. The model demonstrates the feasibility of passive CO_2_ uptake at air-level CO_2_, and shows that at lower external CO_2_ levels, an effective PCCM requires active import of HCO_3_^−^. Both uptake strategies can function at a low energy cost.

While not explicitly considered in our model, protons are produced in Rubisco-catalysed CO_2_-fixing reactions^5^ and are consumed in CAH3-catalysed HCO_3_^−^-to-CO_2_ conversions. Protons must then be depleted in the pyrenoid matrix and replenished in the intrapyrenoid thylakoid lumen to maintain physiological pH values^41,43^. However, our flux-balance analysis shows that the concentrations of free protons are too low to account for the expected proton depletion/replenishment fluxes by free proton diffusion (Supplementary Note VI.D and Fig. 27). Thus, efficient transport of protons must employ alternative mechanisms. One possibility, suggested by recent modelling work^70^, is that proton carriers such as RuBP and 3-PGA could be present at millimolar concentrations^71^ and hence could enable sufficient flux to transport protons between compartments. Understanding the molecular mechanisms underlying proton transport will be an important topic for future studies.

Another class of CCM is the carboxysome-based CCM (CCCM) employed by cyanobacteria^13^. In the CCCM, HCO_3_^−^ becomes concentrated in the cytosol via active transport^72^ and diffuses into carboxysomes—compartments that are typically 100 to 400 nm in diameter, each composed of an icosahedral protein shell enclosing Rubisco^73^. The protein shell is thought to serve as a diffusion barrier, which is necessary for an effective CCCM^46,47^. Whereas the pyrenoid matrix does not appear to have a carbonic anhydrase, the carboxysome matrix contains a carbonic anhydrase that converts HCO_3_^−^ to CO_2_ to locally feed Rubisco. Recent studies suggest that protons produced during Rubisco’s carboxylation could acidify the carboxysome, which in turn favours the carbonic anhydrase-catalysed production of CO_2_^70^. One may ask: what are the benefits of operating a PCCM versus a CCCM? One possibility is that the PCCM uses more complex spatial organization to segregate Rubisco from the thylakoid lumen carbonic anhydrase, which allows the two enzymes to operate at pH values optimal for their respective catalytic functions. Thus, the PCCM may require a smaller Ci pool than the CCCM to produce sufficient CO_2_ in the vicinity of Rubisco. Indeed, cyanobacteria appear to accumulate roughly 30 mM intracellular HCO_3_^−^^74,75^, while *Chlamydomonas* creates an internal HCO_3_^−^ pool of only 1 mM^76^. Future experimentation comparing the performance of the PCCM and the CCCM will advance our understanding of the two distinct mechanisms.

The PCCM has the potential to be transferred into crop plants to improve yields. Our model provides a framework to evaluate overall performance, considering both the efficacy and the energetic efficiency of the PCCM (Supplementary Fig. 28), and allows us to propose a favoured order of engineering steps. Moreover, we expect that our model will help engineers narrow down potential challenges by providing a minimal design for a functional PCCM. If the native plant carbonic anhydrases are inactive or absent, it might be favourable to express and localize other carbonic anhydrases with known activities. Additionally, a key step will be to test whether heterologously expressed *Chlamydomonas* BST channels function as HCO_3_^−^ channels and to verify that they do not interfere with native ion channels in plants. We hope that our model provides practical information for engineers aiming to install a minimal PCCM into plants, and that it will serve as a useful quantitative tool to guide basic PCCM studies in the future.

## Methods

### Reaction-diffusion model

To better understand the operation of the PCCM, we developed a multicompartment reaction-diffusion model on the basis of the postulated mechanism in *Chlamydomonas*. The model takes into account the key PCCM enzymes and transporters and the relevant architecture of the *Chlamydomonas* chloroplast^48^. For simplicity, our model assumes spherical symmetry and considers a spherical chloroplast of radius *R*_chlor_ in an infinite cytosol. Thus, all model quantities can be expressed as functions of the radial distance *r* from the centre of the chloroplast (Fig. 1b). The modelled chloroplast consists of three compartments: a spherical pyrenoid matrix of radius *R*_pyr_ (pH 8) in the centre, surrounded by a stroma (pH 8), with thylakoids (luminal pH 6) traversing both the matrix and stroma (Fig. 1)^41–43^. At steady state, flux-balance equations set the spatially dependent concentrations of CO_2_, HCO_3_^−^, and H_2_CO_3_ in their respective compartments (indicated by subscripts; see Supplementary Table 2 and Note I):

1a $$D^C\nabla _{{{{\mathrm{thy}}}}}^2C_{{{{\mathrm{thy}}}}} - j_{{{{\mathrm{CAH}}}}3} - j_{{{{\mathrm{sp}}}}} - j_{{{{\mathrm{mem}}}}}^Cf_{{{\mathrm{s}}}} = 0$$

1b $$D^C\nabla _{{{{\mathrm{pyr}}}}}^2C_{{{{\mathrm{pyr}}}}} - j_{{{{\mathrm{LCIB}}}}} - j_{{{{\mathrm{sp}}}}} - j_{{{{\mathrm{Rbc}}}}} + j_{{{{\mathrm{mem}}}}}^C\frac{{f_{{{\mathrm{s}}}}f_{{{\mathrm{v}}}}}}{{1 - f_{{{\mathrm{v}}}}}} = 0$$

1c $$D_{{{{\mathrm{str}}}}}^C\nabla _{{{{\mathrm{str}}}}}^2C_{{{{\mathrm{str}}}}} - j_{{{{\mathrm{LCIB}}}}} - j_{{{{\mathrm{sp}}}}} - j_{{{{\mathrm{Rbc}}}}} + j_{{{{\mathrm{mem}}}}}^C\frac{{f_{{{\mathrm{s}}}}f_{{{\mathrm{v}}}}}}{{1 - f_{{{\mathrm{v}}}}}} = 0$$

1d $$D^H\nabla _{{{{\mathrm{thy}}}}}^2H_{{{{\mathrm{thy}}}}} + j_{{{{\mathrm{CAH}}}}3} + j_{{{{\mathrm{sp}}}}} - j_{{{{\mathrm{mem}}}}}^Hf_{{{\mathrm{s}}}} = 0$$

1e $$D^H\nabla _{{{{\mathrm{pyr}}}}}^2H_{{{{\mathrm{pyr}}}}} + j_{{{{\mathrm{LCIB}}}}} + j_{{{{\mathrm{sp}}}}} + j_{{{{\mathrm{mem}}}}}^H\frac{{f_{{{\mathrm{s}}}}f_{{{\mathrm{v}}}}}}{{1 - f_{{{\mathrm{v}}}}}} = 0$$

1f $$D_{{{{\mathrm{str}}}}}^H\nabla _{{{{\mathrm{str}}}}}^2H_{{{{\mathrm{str}}}}} + j_{{{{\mathrm{LCIB}}}}} + j_{{{{\mathrm{sp}}}}} + j_{{{{\mathrm{mem}}}}}^H\frac{{f_{{{\mathrm{s}}}}f_{{{\mathrm{v}}}}}}{{1 - f_{{{\mathrm{v}}}}}} = 0.$$

Here, *C* denotes the concentration of CO_2_, and *H* denotes the combined concentration of HCO_3_^−^ and H_2_CO_3_, which are assumed to be in fast equilibrium^77^. Thus, their respective concentrations are given by $H^ - = \frac{\eta }{{1 + \eta }}H$ for HCO_3_^−^ and $H^0 = \frac{1}{{1 + \eta }}H$ for H_2_CO_3_, where *η* = $10^{{\mathrm{pH-pKa}}_1}$ is a pH-dependent partition factor and pKa_1_ = 3.4 is the negative log of the first acid dissociation constant of H_2_CO_3_^78^. The first terms in equations (1a–1f) describe the diffusive fluxes of inorganic carbon (Ci) within compartments. *D*^*C*^ and *D*^*H*^ respectively denote the diffusion coefficients of CO_2_, and HCO_3_^−^ and H_2_CO_3_ combined, in aqueous solution. In a model with thylakoid stacks slowing Ci diffusion in the stroma, the effective diffusion coefficients *D*_str_^*C*^^/^^*H*^ are obtained using a standard homogenization approach (see Supplementary Fig. 5 and Note I.G); $D_{{{{\mathrm{str}}}}}^{C/H} = D^{C/H}$ otherwise. The other flux terms (*j*_X_) in equations (1a–1f) describe enzymatic reactions and intercompartment Ci transport, and the factors *f*_s_ and *f*_v_ describe the geometry of the thylakoids. Their expressions are provided in subsequent sections.

The boundary conditions at *r* = *R*_pyr_ are determined by the diffusive flux of Ci across the starch sheath at the matrix–stroma interface, that is,

2a $$- D^C\partial _rC_{{{{\mathrm{pyr}}}}} = - D_{{{{\mathrm{str}}}}}^C\partial _rC_{{{{\mathrm{str}}}}} = \kappa _{{{{\mathrm{starch}}}}}\left( {C_{{{{\mathrm{pyr}}}}} - C_{{{{\mathrm{str}}}}}} \right)$$

2b $$- D^H\partial _rH_{{{{\mathrm{pyr}}}}} = - D_{{{{\mathrm{str}}}}}^H\partial _rH_{{{{\mathrm{str}}}}} = \kappa _{{{{\mathrm{starch}}}}}(H_{{{{\mathrm{pyr}}}}} - H_{{{{\mathrm{str}}}}}),$$

where ∂_*r*_ denotes derivative with respect to *r*, and the starch sheath is assumed to have the same permeability *κ*_starch_ for all Ci species. *κ*_starch_→∞ when there is no starch sheath and Ci can diffuse freely out of the matrix. *κ*_starch_ = 0 describes an impermeable starch sheath (see Supplementary Note I.F). Similarly, Ci transport flux across the chloroplast envelope yields the boundary conditions at *r* = *R*_chlor_, that is,

3a $$D_{{{{\mathrm{str}}}}}^\mathrm{C}\partial _rC_{{{{\mathrm{str}}}}} = \kappa ^\mathrm{C}\left( {C_{{{{\mathrm{cyt}}}}} - C_{{{{\mathrm{str}}}}}} \right)$$

3b $$D_{{{{\mathrm{str}}}}}^\mathrm{H}\partial _rH_{{{{\mathrm{str}}}}} = \kappa ^{H^0}\left( {H_{{{{\mathrm{cyt}}}}}^0 - H_{{{{\mathrm{str}}}}}^0} \right) + \kappa ^{H^ - }\left( {H_{{{{\mathrm{cyt}}}}}^ - - H_{{{{\mathrm{str}}}}}^ - } \right) + \kappa _{{{{\mathrm{chlor}}}}}^{H^ - }\left( {H_{{{{\mathrm{cyt}}}}}^ - - \gamma H_{{{{\mathrm{str}}}}}^ - } \right),$$

where $\kappa _{{{{\mathrm{chlor}}}}}^{H^ - }$ and *γ* denote the rate and reversibility of inward HCO_3_^−^ transport from the cytosol, representing the action of the uncharacterized chloroplast envelope HCO_3_^−^ transporter LCIA^24,37^; *γ* = 1 corresponds to a passive bidirectional channel and *γ* < 1 corresponds to an active pump. The external CO_2_ conditions are specified by cytosolic CO_2_ concentration *C*_cyt_. We set *C*_cyt_ = 10 μM for air-level CO_2_ conditions, and *C*_cyt_ = 1 μM for very low CO_2_ conditions. Unless otherwise specified, all cytosolic Ci species are assumed to be in equilibrium at pH 7.1^54^.

#### Thylakoid geometry

The thylakoid geometry has been characterized by cryo-electron tomography in *Chlamydomonas*^48^. In our model, we account for this geometry by varying the local volume fraction *f*_v_ and surface-to-volume ratio *f*_s_ of the thylakoids. These fractions describe a tubule meshwork at the centre of the pyrenoid (*r* ≤ *R*_mesh_), extended radially by *N*_tub_ cylindrical tubules, each of radius *a*_tub_ (see Supplementary Note I.C), that is,

4 $$f_{{{\mathrm{v}}}} = \left\{ {\begin{array}{*{20}{l}} {(N_{{{{\mathrm{tub}}}}}a_{{{{\mathrm{tub}}}}}^2)/(4R_{{{{\mathrm{mesh}}}}}^2)} \hfill & {{{{\mathrm{for}}}}\,r \le R_{{{{\mathrm{mesh}}}}}} \hfill \\ {(N_{{{{\mathrm{tub}}}}}a_{{{{\mathrm{tub}}}}}^2)/(4r^2)} \hfill & {{{{\mathrm{for}}}}\,r > R_{{{{\mathrm{mesh}}}}}} \hfill \end{array}} \right.,{\mathrm{and}}\, f_{\mathrm{s}}= 2/a_{\mathrm{tub}}.$$

In the baseline model, the thylakoid tubules are assumed to extend to the chloroplast envelope, that is, the outer radius of tubules *R*_tub_ = *R*_chlor_. In a model with shorter tubules, we choose $R_{{{{\mathrm{tub}}}}} = 0.4\,R_{{{{\mathrm{chlor}}}}}$, and set *f*_v_ = 0 and *f*_s_ = 0 for *r* > *R*_tub_. Thus, the Laplace–Beltrami operators in equation (1) are given by $\nabla _{{{{\mathrm{thy}}}}}^2 = r^{ - 2}f_{{{\mathrm{v}}}}^{ - 1}\partial _rf_{{{\mathrm{v}}}}r^2\partial _r$ for the thylakoid tubules, and by $\nabla _{{{{\mathrm{pyr}}}}}^2 = \nabla _{{{{\mathrm{str}}}}}^2 = r^{ - 2}(1 - f_{{{\mathrm{v}}}})^{ - 1}\partial _r(1 - f_{{{\mathrm{v}}}})r^2\partial _r$ for the matrix and stroma.

#### Enzyme kinetics

The model considers three key *Chlamydomonas* PCCM enzymes, that is, the carbonic anhydrases (CAs) CAH3 and LCIB and the CO_2_-fixing enzyme Rubisco. The interconversion between CO_2_ and HCO_3_^−^ is catalysed by both CAs and follows reversible Michaelis-Menten kinetics^79^. The rate of CA-mediated CO_2_-to-HCO_3_^−^ conversion is given by

5 $$\begin{array}{*{20}{c}} {j_{{{{\mathrm{CA}}}}}(C,H^ - ) = \frac{{(V_{{{{\mathrm{max}}}},{{{\mathrm{CA}}}}}^C/K_{{{\mathrm{m}}}}^C)(C - K^{{{{\mathrm{eq}}}}}H^ - )}}{{1 + C/K_{{{\mathrm{m}}}}^C + H^ - /K_{{{\mathrm{m}}}}^{H^ - }}}{{{\mathcal{L}}}}_{{{{\mathrm{CA}}}}},} \end{array}$$

where $V_{{{{\mathrm{max}}}},{{{\mathrm{CA}}}}}^C$ denotes the maximum rate of CA, $K_{{{\mathrm{m}}}}^C$ and $K_{{{\mathrm{m}}}}^{H^ - }$ respectively denote the half-saturation concentrations for CO_2_ and HCO_3_^−^, and $V_{{{{\mathrm{max}}}},{{{\mathrm{CA}}}}}^C/K_{{{\mathrm{m}}}}^C$ denotes the first-order rate constant which we refer to as the ‘rate’ of the CA (Fig. 2). Finally, $K^{{{{\mathrm{eq}}}}} = 10^{{{{\mathrm{pK}}}}_{{{{\mathrm{eff}}}}} - {{{\mathrm{pH}}}}}$ denotes the equilibrium ratio of CO_2_ to HCO_3_^−^, where the effective pKa is given by ${{{\mathrm{pK}}}}_{{{{\mathrm{eff}}}}} = 6.1$^80,81^. The localization function ${{{\mathcal{L}}}}_{{{{\mathrm{CA}}}}}$ is equal to one for *r* where CA is present and zero elsewhere. The uncatalysed spontaneous rate of CO_2_-to-HCO_3_^−^ conversion, with a first-order rate constant $k_{{{{\mathrm{sp}}}}}^C$, is given by $j_{{{{\mathrm{sp}}}}} = k_{{{{\mathrm{sp}}}}}^C(C - K^{{{{\mathrm{eq}}}}}H^ - )$^82^. Note that negative values of *j*_CA_ and *j*_sp_ denote fluxes of CO_2_-to-HCO_3_^−^ conversion.

The rate of CO_2_ fixation catalysed by Rubisco is calculated from

6 $$\begin{array}{*{20}{c}} {j_{{{{\mathrm{Rbc}}}}}(C) = V_{{{{\mathrm{max}}}},{{{\mathrm{Rbc}}}}}^C\frac{C}{{K_{{{\mathrm{m}}}}^{{{{\mathrm{eff}}}}} + C}}{{{\mathcal{L}}}}_{{{{\mathrm{Rbc}}}}}.} \end{array}$$

Here, $V_{{{{\mathrm{max}}}},{{{\mathrm{Rbc}}}}}^C$ denotes the maximum rate, and the effective *K*_m_ (Rubisco *K*_m_ in Fig. 1) is given by $K_{{{\mathrm{m}}}}^{{{{\mathrm{eff}}}}} = K_{{{{\mathrm{m}}}},{{{\mathrm{Rbc}}}}}^C(1 + O/K_{{{{\mathrm{m}}}},{{{\mathrm{Rbc}}}}}^O)$ to account for competitive inhibition by O_2_^83,84^, where *O* denotes the concentration of O_2_, and $K_{{{{\mathrm{m}}}},{{{\mathrm{Rbc}}}}}^C$ and $K_{{{{\mathrm{m}}}},{{{\mathrm{Rbc}}}}}^O$ denote the half-saturation substrate concentrations for CO_2_ and O_2_, respectively. ${{{\mathcal{L}}}}_{{{{\mathrm{Rbc}}}}}$ is equal to one where Rubisco is localized, and zero elsewhere.

In our baseline model, we assume that CAH3 is localized in the thylakoid tubules traversing the pyrenoid^40^, LCIB is distributed diffusely in the stroma^57^ and Rubisco is localized in the pyrenoid matrix^16^. To explore the effect of enzyme localization, we vary the start and end radii of the enzymes while maintaining a constant number of molecules (Figs. 4 and 5, and Supplementary Note III).

#### Transport of Ci across thylakoid membranes

The flux of CO_2_ diffusing across the thylakoid membrane from the thylakoid lumen to the matrix or stroma is given by

7 $$j_{{{{\mathrm{mem}}}}}^C = \left\{ {\begin{array}{*{20}{l}} {\kappa ^C(C_{{{{\mathrm{thy}}}}} - C_{{{{\mathrm{pyr}}}}})} \hfill & {{{{\mathrm{for}}}}\,r \le R_{{{{\mathrm{pyr}}}}}} \hfill \\ {\kappa ^C(C_{{{{\mathrm{thy}}}}} - C_{{{{\mathrm{str}}}}})} \hfill & {{{{\mathrm{for}}}}\,r > R_{{{{\mathrm{pyr}}}}}} \hfill \end{array}} \right.,$$

where *κ*^*C*^ denotes the permeability of thylakoid membranes to CO_2_. Similarly, the cross-membrane diffusive flux of HCO_3_^−^ and H_2_CO_3_, $j_{{{{\mathrm{mem}}}}}^H$, is given by

8 $$\begin{array}{*{20}{c}} {j_{{{{\mathrm{mem}}}}}^{H} = \left\{ {\begin{array}{*{20}{l}} {(\kappa ^{H^ - } + \kappa _{{{{\mathrm{thy}}}}}^{H^ - })(H_{{{{\mathrm{thy}}}}}^{-} - H_{{{{\mathrm{pyr}}}}}^ - ) + \kappa ^{H^0}(H_{{{{\mathrm{thy}}}}}^0 - H_{{{{\mathrm{pyr}}}}}^0)} \hfill & {{{{\mathrm{for}}}}\,r \le R_{{{{\mathrm{pyr}}}}}} \hfill \\ {(\kappa ^{H^ - } + \kappa _{{{{\mathrm{thy}}}}}^{H^ - })(H_{{{{\mathrm{thy}}}}}^ - - H_{{{{\mathrm{str}}}}}^ - ) + \kappa ^{H^0}(H_{{{{\mathrm{thy}}}}}^0 - H_{{{{\mathrm{str}}}}}^0)} \hfill & {{{{\mathrm{for}}}}\,r > R_{{{{\mathrm{pyr}}}}}} \hfill \end{array}} \right.,} \end{array}$$

where $\kappa^{H^-}$ and $\kappa^{H^0}$ respectively denote the baseline membrane permeability to HCO_3_^−^ and H_2_CO_3_, and $\kappa _{{{{\mathrm{thy}}}}}^{H^ - }$ denotes the additional permeability of thylakoid membranes to HCO_3_^−^ due to bestrophin-like channels^25^. Note that the final terms of equations (1a) and (1a–1c) differ by a factor of $\frac{{f_{{{\mathrm{v}}}}}}{{1 - f_{{{\mathrm{v}}}}}}$ because the cross-membrane fluxes have a larger impact on the concentrations in the thylakoid compartment, which has a smaller volume fraction.

#### Choice of parameters and numerical simulations

The model parameters were estimated from experiment (see Supplementary Table 2 and references therein), except for the rates of LCIB and CAH3 and the kinetic parameters of the HCO_3_^−^ transporters, which are not known. We performed a systematic scan for these unknown parameters within a range of reasonable values (Fig. 2 and Supplementary Fig. 4). The numerical solutions of equation (1) were obtained by performing simulations using a finite element method. Partial differential equations were converted to their equivalent weak forms, computationally discretized by first-order elements^85^ and implemented in the open-source computing platform FEniCS^86^. A parameter sensitivity analysis was performed to verify the robustness of the model results (Supplementary Fig. 30). A convergence study was performed to ensure sufficient spatial discretization (Supplementary Fig. 31).

### Energetic cost of the CCM

We computed the energetic cost using the framework of nonequilibrium thermodynamics^56^ (see Supplementary Note II.B for details). In brief, the free-energy cost of any nonequilibrium process (reaction, diffusion, or transport) is given by (*j*_+_ −*j*_−_)ln(*j*_+_/*j*_−_) (in units of thermal energy *RT*), where *j*_+_ and *j*_−_ denote the forward and backward flux, respectively. Summing the energetic cost of nonequilibrium processes described in equation (1), we show that the total energy required to operate the PCCM can be approximated (in units of *RT*) by

$$\begin{array}{*{20}{c}} {\dot W_{{{{\mathrm{PCCM}}}}} \approx J_{{{{\mathrm{str}}}}}^{C \to H^ - }{{{\mathrm{ln}}}}\frac{{K_{{{{\mathrm{thy}}}}}^{{{{\mathrm{eq}}}}}}}{{K_{{{{\mathrm{str}}}}}^{{{{\mathrm{eq}}}}}}} + J_{{{{\mathrm{chlor}}}}}^C{{{\mathrm{ln}}}}\frac{{\gamma ^{ - 1}K_{{{{\mathrm{thy}}}}}^{{{{\mathrm{eq}}}}}}}{{K_{{{{\mathrm{str}}}}}^{{{{\mathrm{eq}}}}}}} + J_{{{{\mathrm{Rbc}}}}}{{{\mathrm{ln}}}}\frac{{\gamma ^{ - 1}K_{{{{\mathrm{thy}}}}}^{{{{\mathrm{eq}}}}}}}{{K_{{{{\mathrm{str}}}}}^{{{{\mathrm{eq}}}}}}},} \end{array}$$

Here, $J_{{{{\mathrm{str}}}}}^{C \to H^ - } = - {\int}_0^{R_{{{{\mathrm{chlor}}}}}} {4\mathrm{\pi} r^2(1 - f_{{{\mathrm{v}}}})(j_{{{{\mathrm{LCIB}}}}} + j_{{{{\mathrm{sp}}}}})dr}$ integrates the flux of LCIB-mediated and spontaneous conversion from CO_2_ to HCO_3_^−^ in the stroma, with 4π*r*^2^(1 − *f*_v_)*dr* being the geometric factor. $J_{{{{\mathrm{chlor}}}}}^C = 4\pi R_{{{{\mathrm{chlor}}}}}^2\kappa ^C(C_{{{{\mathrm{str}}}}}|_{r = R_{{{{\mathrm{chlor}}}}}} - C_{{{{\mathrm{cyt}}}}})$ denotes the flux of CO_2_ diffusing from the stroma back out into the cytosol. $J_{{{{\mathrm{Rbc}}}}} = {\int}_0^{R_{{{{\mathrm{chlor}}}}}} {4\pi r^2(1 - f_{{{\mathrm{v}}}})j_{{{{\mathrm{Rbc}}}}}dr}$ integrates the flux of CO_2_ fixation by Rubisco. The ln*γ*^−1^ and ${{{\mathrm{ln}}}}({K_{{{{\mathrm{thy}}}}}^{{{{\mathrm{eq}}}}}}/{K_{{{{\mathrm{str}}}}}^{{{{\mathrm{eq}}}}}})$ terms denote the free-energy cost of pumping HCO_3_^−^ across the chloroplast envelope and pumping protons across the thylakoid membranes, respectively. Using ATP hydrolysis energy $|{\Delta}G_{ATP}| = 51.5\,RT$^87^, we compute the equivalent ATP spent per CO_2_ fixed as $\dot W_{{{{\mathrm{PCCM}}}}}/J_{{{{\mathrm{Rbc}}}}}/|{\Delta}G_{{{{\mathrm{ATP}}}}}|$.

### Well-mixed compartment model

To better understand the biophysical limit of the PCCM, we consider a well-mixed compartment simplification of the full model. Specifically, we assume that (i) the diffusion of Ci is fast in the matrix and stroma, and therefore the concentrations of CO_2_ and HCO_3_^−^ are constant across radii in each of the two compartments, taking values denoted by $C_{{{{\mathrm{pyr}}}}},C_{{{{\mathrm{str}}}}},H_{{{{\mathrm{pyr}}}}}^ -$ and $H_{{{{\mathrm{str}}}}}^ -$; (ii) HCO_3_^−^ transport across the thylakoid membranes is fast, and thus the thylakoid tubule concentration of HCO_3_^−^ inside the pyrenoid is equal to $H_{{{{\mathrm{pyr}}}}}^ -$, while the thylakoid tubule concentration outside the pyrenoid is equal to $H_{{{{\mathrm{str}}}}}^ -$; (iii) HCO_3_^−^ and CO_2_ are in equilibrium (catalysed by CAH3) in the thylakoid tubules inside the pyrenoid, and thus the CO_2_ concentration therein is given by $C_{{{{\mathrm{thy}}}}} = K_{{{{\mathrm{thy}}}}}^{{{{\mathrm{eq}}}}}H_{{{{\mathrm{pyr}}}}}^ -$; and (iv) the concentration of CO_2_ in the thylakoid tubules approaches *C*_str_ toward the chloroplast envelope. Thus, the flux-balance conditions are described by a set of algebraic equations of 4 variables, $C_{{{{\mathrm{pyr}}}}},C_{{{{\mathrm{thy}}}}},C_{{{{\mathrm{str}}}}}$ and $H_{{{{\mathrm{str}}}}}^ -$ (see Supplementary Notes IV and V). The algebraic equations are solved using the Python-based computing library SciPy (version 1.5.0)^88^. The energetic cost of the well-mixed compartment model is computed similarly as above.

### Engineering paths

We are interested in how adding and removing individual components affects the overall functioning of the PCCM. We thus measured the efficacy and energy efficiency of 216 PCCM configurations, modulating the presence and localization of enzymes, HCO_3_^−^ channels and diffusion barriers. Each configuration was simulated using the reaction-diffusion model above, with the appropriate parameters for that strategy (Supplementary Fig. 26).

To find all possible engineering paths between these configurations, we considered a graph on which each possible configuration is a node. Nodes were considered to be connected by an undirected edge if they were separated by one engineering step. Thus, by taking steps on the graph, we searched all possible engineering paths, given a start node with poor PCCM performance and a target node with good performance. A single engineering step could be the addition or removal of an enzyme, a channel, or a diffusion barrier, as well as the localization of a single enzyme. The exception is the localization of Rubisco, which we assumed can exclude LCIB from the matrix as it forms a phase-separated condensate^26^. We did not consider strategies employing both a starch sheath and thylakoid stacks as diffusion barriers. We used a custom depth-first search algorithm in MATLAB (R2020a) to identify all shortest engineering paths between a start and a target node.

### Reporting Summary

Further information on research design is available in the Nature Research Reporting Summary linked to this article.

## Supplementary information

Supplementary Information Supplementary Notes I–VI, Figs. 1–31 and Tables 1–4.

Reporting Summary

Supplementary Table 5 and 6 Supplementary Tables 5 and 6.

## Data Availability

All data generated or analysed during this study are included in this Article and the supplementary tables. The raw datasets have been deposited in the Zenodo repository at 10.5281/zenodo.6406849.

## Code availability

Custom simulation codes are available on GitHub at https://github.com/f-chenyi/Chlamydomonas-CCM.
