# Supplementary material for: Modelling the pyrenoid-based CO2-concentrating mechanism provides insights into its operating principles and a roadmap for its engineering into crops
Source: Nat Plants. 2022 May 19;8(5):583–95. doi: 10.1038/s41477-022-01153-7 (PMC9122830; doi:10.1038/s41477-022-01153-7)
Supplement: Supplementary file 1 — Supplementary Notes I–VI, Figs. 1–31 and Tables 1–4. [file 41477_2022_1153_MOESM1_ESM.pdf]

nature portfolio

https://doi.org/10.1038/s41477-022-01153-7

Supplementary information

Modelling the pyrenoid-based CO_2-concentrating mechanism provides insights into its operating principles and a roadmap for its engineering into crops

In the format provided by the authors and unedited

Supplementary Information for

Modeling the pyrenoid-based CO_2-concentrating mechanism provides insights into its operating principles and a roadmap for its engineering into crops

Chenyi Fei[a,b,†], Alexandra T. Wilson[a,c,†], Niall M. Mangan[d,*], Ned S. Wingreen[a,b,*], Martin C. Jonikas[a,e,*]

[a]Department of Molecular Biology, Princeton University, Princeton, NJ 08544; [b]Lewis-Sigler Institute for Integrative Genomics, Princeton University, Princeton, NJ 08544; [c]Department of Biology, Massachusetts Institute of Technology, Cambridge, MA 02139; [d]Department of Engineering Sciences and Applied Mathematics, Northwestern University, Evanston, IL 60208; [e]Howard Hughes Medical Institute, Chevy Chase, MD 20815

[†]These authors contributed equally.
[*]To whom correspondence may be addressed. Email: niall.mangan@northwestern.edu, wingreen@princeton.edu, or mjonikas@princeton.edu.

TABLE OF CONTENTS

List of Supplementary Figures 2

List of Supplementary Tables 3

I. A multi-compartment reaction-diffusion model of the Chlamydomonas PCCM 4
A. General formulation 4
B. Enzyme kinetics 5
C. Thylakoid geometry 6
D. Diffusion and transport of inorganic carbon across the thylakoid membranes 7
E. Diffusion and transport of inorganic carbon across the chloroplast envelope 7
F. Starch sheath: a potential barrier to diffusion of inorganic carbon out of the pyrenoid matrix 8
G. Thylakoid stacks: a potential barrier to slow diffusion of inorganic carbon in the stroma 8
H. Model discussion: CO_2 leakage 9
I. Model discussion: Effect of different cytosolic Ci compositions 10

II. Evaluating the performance of the PCCM 11
A. Normalized CO_2 fixation flux and net carbon fixation flux 11
B. Energetic cost of the PCCM 11
C. A combined measure of PCCM performance 15

III. Localization and regulation of key PCCM enzymes 18
A. Localization and regulation of CAH3 18
B. Localization of LCIB 18

IV. Well-mixed compartment model with an impermeable starch sheath 20
A. Governing equations 20
B. Two extreme PCCM strategies 21
C. Feasible PCCM strategies under varying CO_2 conditions 21
D. Limits of the passive CO_2 uptake strategy 22

V. Well-mixed compartment model with thylakoid stacks 23
A. Diffusion length scale in the stroma 23
B. Flux-balance equations 23
C. Results and discussion 25

VI. Supplementary discussion 27
A. Alternative thylakoid morphologies 27
B. Import of bicarbonate into the cell 28
C. Ci diffusion in the pyrenoid 29
D. Proton transport 29
E. Effect of LCIB-Rubisco complexes 30

Supplementary figures and tables 32

References 67

LIST OF SUPPLEMENTARY FIGURES

1 CO_2 fixation by Rubisco is inhibited by oxygen at air-level CO_2. 32
2 Volume fraction of the modeled chloroplast compartments. 32
3 Low fixation efficiency at air-level CO_2 (10 μM cytosolic) in the absence of a diffusion barrier is due to severe CO_2 leakage from the pyrenoid matrix. 33
4 CO_2 fixation flux increases with BST-mediated thylakoid membrane permeability to HCO_3^-. 34
5 Model for the effect of thylakoid stacks on diffusion in the stroma. 35
6 CO_2 concentrating performance is compromised by weaker barriers to the diffusion of inorganic carbon. 36
7 Comparison between two types of diffusion barriers under air-level CO_2 (10 μM cytosolic). 37
8 A model including both thylakoid stacks and an impermeable starch sheath behaves similarly to a model with only the latter. 38
9 In a modeled chloroplast employing thylakoid stacks and no starch sheath, LCIB activity increases intra-chloroplast HCO_3^- concentration by converting external CO_2 but also effectively "steals" CO_2 from the pyrenoid matrix, leading to a nonmonotonic impact of LCIB rate on CO_2 concentration inside the pyrenoid matrix. 39
10 A trade-off between CO_2 transport and CO_2 leakage leads to the existence of an intermediate membrane permeability to CO_2 that optimizes CO_2-concentrating performance. 40
11 Localizing LCIB close to the chloroplast envelope prevents excessive CO_2 efflux drawn by LCIB from the pyrenoid matrix in a modeled chloroplast with thylakoid stacks slowing diffusion in the stroma and no starch sheath. 41
12 Active HCO_3^- transport across the chloroplast envelope enables an effective PCCM under very low CO_2. 42
13 Illustrations of the nonequilibrium processes that cost free energy. 43
14 The least costly PCCM strategies. 44
15 The composition of cytosolic Ci impacts the efficacy of Ci import strategies. 45
16 CAH3 localization and activity that enable an effective PCCM depend on environmental CO_2 levels. . . 47
17 Localization of carbonic anhydrases enhances PCCM performance under very low CO_2. 48
18 A strong diffusion barrier limits the dependence of PCCM activity on LCIB localization for a passive CO_2 uptake strategy. 49
19 Localization of LCIB around the pyrenoid in a model with a starch sheath allows for CO_2 recycling without inducing carbon leakage from the chloroplast when the active HCO_3^- pumping strategy is employed. 50
20 High PCCM performance requires low-pH thylakoids and high-pH stroma. 51
21 A well-mixed compartment model of the PCCM. 52
22 Two extreme CO_2 uptake strategies are compared under various CO_2 conditions for a model employing an impermeable starch sheath. 53
23 Feasible PCCM strategies depend on external CO_2 levels in the well-mixed compartment model. 54
24 Feasible PCCM strategies depend on external CO_2 levels in the full reaction-diffusion model. 55
25 Alternative thylakoid morphologies also support an effective PCCM. 56
26 Characterizing various PCCM configurations yields feasible engineering paths for installing a PCCM into plant chloroplast. 57
27 Diffusion fluxes of free protons are not sufficient to maintain compartment pH. 58
28 Modeled cell growth rate is determined jointly by the efficacy and energy cost of the PCCM. 59
29 Protons derived from Rubisco CO_2 fixation lead to a notable increase in local proton concentration and CO_2 availability only in 10 μm CA-Rubisco condensates. 60
30 Parameter sensitivity analysis of the multi-compartment reaction-diffusion model. 61
31 Mesh convergence of the finite-element simulations. 62

LIST OF SUPPLEMENTARY TABLES

1 Key Chlamydomonas PCCM proteins considered in the model. 63
2 Summary of the notations and parameter values used in the reaction-diffusion model of the PCCM. Subheadings of parameter groups are in bold. 64
3 Summary of the notations and parameter values used in the combined metric calculation (Sec. II C). Subheadings of parameter groups are in bold. 65
4 Summary of the parameters and their values used in the well-mixed compartment model. Subheadings of parameter groups are in bold. 66

I. A MULTI-COMPARTMENT REACTION-DIFFUSION MODEL OF THE CHLAMYDOMONAS PCCM

Many eukaryotic algae operate a pyrenoid-based CO_2-concentrating mechanism (PCCM) to supply the central carbon-fixing enzyme, Rubisco, with a high concentration of its substrate, CO_2. To better understand how the PCCM works, we build a multi-compartment reaction-diffusion model consisting of the essential mechanics of the PCCM in the best-characterized model alga Chlamydomonas (see Fig. 1A), including the relevant architecture of the Chlamydomonas chloroplast. In brief, we model the chloroplast as a sphere of radius R_{chlor}, comprised of three compartments — a spherical pyrenoid matrix of radius R_{pyr} in the center, surrounded by a stroma, with thylakoids traversing both matrix and stroma [1]. We assume a constant pH in each compartment, with the matrix and stroma at pH 8 [2] and the thylakoids at pH 6 [3]. In the model, we consider intra-compartment diffusion and inter-compartment exchange of inorganic carbon in the forms of CO_2, H_2CO_3, and HCO_3^-, as well as their interconversion catalyzed by CAH3 in the thylakoids and by LCIB in the stroma (see Figs. 1A and B). Carbonate (CO_3^{2-}) can be neglected at the pH values we consider [4].

Below we describe our model in full: in Sec. I A we derive the steady-state reaction-diffusion kinetics of inorganic carbon; in Sec. I B we describe the reaction kinetics of key PCCM enzymes; in Sec. I C we specify the modeled geometry of the thylakoids; in Secs. I D and I E we detail the inter-compartment diffusion and transport of inorganic carbon; in Secs. I F and I G we introduce the presence of potential barriers to inorganic carbon diffusion; and in Sec. I H we discuss the issue of CO_2 leakage from the pyrenoid matrix. Notations and parameter values used in our model are provided in Supplementary Table 2.

A. General formulation

We denote by C the concentration of CO_2, by H^- the concentration of HCO_3^-, by H^0 the concentration of H_2CO_3, and by H the total concentration of HCO_3^- and H_2CO_3, with respective compartments indicated by subscripts. For simplicity, our model assumes radial symmetry, i.e., concentrations of inorganic carbon are functions of radial coordinate r only, $C = C(r)$ and $H = H(r)$. Thus, at steady state, the mass conservation of thylakoid CO_2 between r and $r + dr$ is given by

$$\left[(4\pi r^2 f_{\mathrm{v}}) D^C \frac{\partial C_{\mathrm{thy}}}{\partial r}\right]_r^{r+dr} - (j_{\mathrm{sp}} + j_{\mathrm{CAH3}})(4\pi r^2 f_{\mathrm{v}} dr) - j_{\mathrm{mem}}^C ds(r, dr) = 0, \tag{S1}$$

where $f_{\mathrm{v}}(r)$ denotes the volume fraction of the thyalkoids, $ds(r, dr)$ denotes the surface area of thylakoid membrane between r and $r+dr$, D^C denotes the diffusion coefficient of CO_2, j_{mem}^C denotes the transport flux across the thylakoid membrane, j_{sp} denotes the flux of spontaneous conversion of CO_2 to HCO_3^-,and j_{CAH3} denotes the flux of conversion of CO_2 to HCO_3^- catalyzed by CAH3. j_{mem} is positive for a flux from the thylakoid lumen into the matrix or stroma. Defining $f_{\mathrm{s}} \equiv \frac{ds(r,dr)}{4\pi r^2 f_{\mathrm{v}} dr}$, we obtain

$$D^C \frac{1}{r^2 f_{\mathrm{v}}} \frac{\partial}{\partial r}\left(r^2 f_{\mathrm{v}} \frac{\partial C_{\mathrm{thy}}}{\partial r}\right) - j_{\mathrm{sp}} - j_{\mathrm{CAH3}} - j_{\mathrm{mem}}^C f_{\mathrm{s}} = 0. \tag{S2}$$

We provide the expressions for j_{sp} and j_{CAH3} in Sec. I B, the expressions for f_{v} and f_{s} in Sec. I C, and the expression for j_{mem}^C in Sec. I D. Similarly, the steady-state flux-balance conditions for CO_2 in the pyrenoid matrix ($r \leqslant R_{\mathrm{pyr}}$) and in the stroma ($R_{\mathrm{pyr}} \leqslant r \leqslant R_{\mathrm{chlor}}$) yield, respectively,

$$D^C \frac{1}{(1-f_{\mathrm{v}})r^2} \frac{\partial}{\partial r}\left((1-f_{\mathrm{v}}) r^2 \frac{\partial C_{\mathrm{pyr}}}{\partial r}\right) - j_{\mathrm{sp}} - j_{\mathrm{LCIB}} - j_{\mathrm{Rbc}} + j_{\mathrm{mem}}^C \frac{f_{\mathrm{s}} f_{\mathrm{v}}}{1-f_{\mathrm{v}}} = 0, \text{ and} \tag{S3}$$

$$D_{\mathrm{str}}^C \frac{1}{(1-f_{\mathrm{v}})r^2} \frac{\partial}{\partial r}\left((1-f_{\mathrm{v}}) r^2 \frac{\partial C_{\mathrm{str}}}{\partial r}\right) - j_{\mathrm{sp}} - j_{\mathrm{LCIB}} - j_{\mathrm{Rbc}} + j_{\mathrm{mem}}^C \frac{f_{\mathrm{s}} f_{\mathrm{v}}}{1-f_{\mathrm{v}}} = 0, \tag{S4}$$

where j_{LCIB} denotes the flux of conversion of CO_2 to HCO_3^- catalyzed by LCIB and j_{Rbc} denotes the flux of CO_2 fixation by Rubisco. Their expressions are given in Sec. I B. D_{str}^C denotes the diffusion coefficient of CO_2 in the stroma (see **Choice of parameters** below).

Steady-state flux-balance conditions for HCO_3^- and H_2CO_3 in the thylakoids are given, respectively, by

$$D^{H^-}\frac{1}{r^2 f_\mathrm{v}}\frac{\partial}{\partial r}\left(r^2 f_\mathrm{v}\frac{\partial H^-_\mathrm{thy}}{\partial r}\right)+j_\mathrm{sp}+j_\mathrm{CAH3}-j^{H^-}_\mathrm{mem}f_\mathrm{s}+j^H_\mathrm{int}=0,\ \text{and} \tag{S5}$$

$$D^{H^0}\frac{1}{r^2 f_\mathrm{v}}\frac{\partial}{\partial r}\left(r^2 f_\mathrm{v}\frac{\partial H^0_\mathrm{thy}}{\partial r}\right)-j^{H^0}_\mathrm{mem}f_\mathrm{s}-j^H_\mathrm{int}=0. \tag{S6}$$

Here, D^{H^-} and D^{H^0} denote the diffusion coefficients of HCO_3^- and H_2CO_3, respectively. j^H_int denotes the flux of interconversion between HCO_3^- and H_2CO_3. Since this reaction is very fast [4, 5], we set all the other terms to zero in Eqs. (S5) and (S6) and solve for the relation between H^- and H^0, which yields $H^- = 10^{\mathrm{pH}-\mathrm{pKa}_1}H^0 \equiv \eta H^0$, i.e., $H^- = \frac{\eta}{1+\eta}H$ and $H^0 = \frac{1}{1+\eta}H$, where $\mathrm{pKa}_1 = 3.4$ is the first pKa of H_2CO_3 and η is a constant factor depending on the compartmental pH. Summing Eqs. (S5) and (S6) yields

$$D^{H}\frac{1}{r^2 f_\mathrm{v}}\frac{\partial}{\partial r}\left(r^2 f_\mathrm{v}\frac{\partial H_\mathrm{thy}}{\partial r}\right)+j_\mathrm{sp}+j_\mathrm{CAH3}-j^{H}_\mathrm{mem}=0, \tag{S7}$$

where $D^H = \frac{\eta}{1+\eta}D^{H^-} + \frac{1}{1+\eta}D^{H^0}$ is the effective diffusion coefficient of the two species, and $j^H_\mathrm{mem} = j^{H^-}_\mathrm{mem} + j^{H^0}_\mathrm{mem}$ denotes combined transport flux whose expression is provided in Sec. I D. Likewise, we obtain the following steady-state flux-balance conditions in the matrix and stroma:

$$D^{H}\frac{1}{(1-f_\mathrm{v})r^2}\frac{\partial}{\partial r}\left((1-f_\mathrm{v})r^2\frac{\partial H_\mathrm{pyr}}{\partial r}\right)+j_\mathrm{sp}+j_\mathrm{LCIB}+j^{H}_\mathrm{mem}\frac{f_\mathrm{s}f_\mathrm{v}}{1-f_\mathrm{v}}=0,\ \text{and} \tag{S8}$$

$$D^{H}_\mathrm{str}\frac{1}{(1-f_\mathrm{v})r^2}\frac{\partial}{\partial r}\left((1-f_\mathrm{v})r^2\frac{\partial H_\mathrm{str}}{\partial r}\right)+j_\mathrm{sp}+j_\mathrm{LCIB}+j^{H}_\mathrm{mem}\frac{f_\mathrm{s}f_\mathrm{v}}{1-f_\mathrm{v}}=0. \tag{S9}$$

Here, D^H_str denotes the diffusion coefficient of HCO_3^- and H_2CO_3 in the stroma (see **Choice of parameters** below). Note that one can use the relations $H^- = \frac{\eta}{1+\eta}H$ and $H^0 = \frac{1}{1+\eta}H$ to express Eqs. (S7 - S9) as closed equations for H.

Choice of parameters: The sizes of the chloroplast and the pyrenoid matrix are estimated to be $R_\mathrm{chlor} = 3.14$ µm and $R_\mathrm{pyr} = 0.3R_\mathrm{chlor}$ from the experimental images in [6]. Diffusion coefficients of CO_2 and HCO_3^- in aqueous solution are measured to be $D^C = 1.88 \times 10^3$ µm^2/s [7] and $D^{H^-} = 1.15 \times 10^3$ µm^2/s [8]. We approximate the diffusion coefficient of H_2CO_3 by that of acetic acid due to their identical charge and similar size, i.e., $D^{H^0} \approx 1.2 \times 10^3$ µm^2/s [9]. Thus, for simplicity, we set $D^{H^0} = D^{H^-}$, which results in $D^H \equiv D^{H^-}$. In the presence of thylakoid stacks slowing inorganic carbon diffusion in the stroma, the effective diffusion coefficients $D^{C/H}_\mathrm{str} = D^{C/H}_\mathrm{eff}$ are computed in Sec. I G; $D^{C/H}_\mathrm{str} = D^{C/H}$ otherwise.

B. Enzyme kinetics

Carbonic anhydrases: The two carbonic anhydrases (CAs) CAH3 and LCIB catalyze the interconversion between CO_2 and HCO_3^- [10, 11] and are known to be key to proper functioning of the PCCM [12, 13]. Since the full reaction $CO_2 + H_2O \leftrightarrow HCO_3^- + H^+$ involves a proton, the equilibrium CO_2-to-HCO_3^- ratio $K^\mathrm{eq} \equiv 10^{(\mathrm{pK_{eff}}-\mathrm{pH})}$ depends on the compartmental pH, where $\mathrm{pK_{eff}} = 6.1$ is the effective $\mathrm{pK_a}$, defined as the pH at which CO_2 and HCO_3^- have equal equilibrium concentrations [4]. We assume the reactions catalyzed by CAs follow Michaelis–Menten kinetics, so that

$$j_\mathrm{CA}(C,H^-) = \frac{\left(V^C_\mathrm{max,CA}/K^C_\mathrm{m}\right)\left(C-K^\mathrm{eq}H^-\right)}{1+C/K^C_\mathrm{m}+H^-/K^{H^-}_\mathrm{m}}\mathcal{L}_\mathrm{CA}, \tag{S10}$$

where $V^C_\mathrm{max,CA}$ denotes the maximum rate at which CO_2 is converted to HCO_3^-, K^C_m and $K^{H^-}_\mathrm{m}$ denote the half-saturation substrate concentrations for CO_2 and HCO_3^-, respectively, and $V^C_\mathrm{max,CA}/K^C_\mathrm{m}$ denotes the first-order rate constant which we refer to as the "rate" of that enzyme (see for example Fig. 2). Here, $\mathcal{L}_\mathrm{CA}$ denotes the localization of CAs (see **Enzyme localization** below). Note that the uncatalyzed spontaneous conversion of CO_2 to HCO_3^- is much slower than the reactions catalyzed by CA. Nevertheless, we include it in our model for completeness, with a flux of $j_\mathrm{sp} = k^C_\mathrm{sp}(C - K^\mathrm{eq}H^-)$.

Rubisco: Rubisco not only catalyzes the reaction of CO_2 fixation but also catalyzes the oxygenation reaction in

which O_2 competes with CO_2 for the active sites of Rubisco [14]. Thus, the flux of CO_2 fixation by Rubisco $j_{\rm Rbc}$ depends on the concentrations of both CO_2 and O_2. The Michaelis-Menten kinetics of competitive inhibition reads (Supplementary Fig. 1) [14]

$$j_{\rm Rbc} = \frac{V^C_{\rm max,Rbc} C}{K^C_{\rm m,Rbc}(1 + O/K^O_{\rm m,Rbc}) + C} \mathcal{L}_{\rm Rbc}, \tag{S11}$$

where $V^C_{\rm max,Rbc}$ denotes the maximum rate of CO_2 fixation, O denotes the concentration of O_2, and $K^C_{\rm m,Rbc}$ and $K^O_{\rm m,Rbc}$ denote the half-saturation substrate concentrations for CO_2 and O_2, respectively. We term $K^C_{\rm m,Rbc}(1 + \frac{O}{K^O_{\rm m,Rbc}})$ the effective $K_{\rm m}$ of CO_2 fixation (denoted by $K^{\rm eff}_{\rm m}$) or simply "Rubisco $K_{\rm m}$" (see Fig. 1 and Supplementary Fig. 1). Similar to above, $\mathcal{L}_{\rm Rbc}$ denotes the localization of Rubisco (see **Enzyme localization** below).

Enzyme localization: In our spherically symmetric model, an enzyme ranging from $R_{\rm s}$ to $R_{\rm e}$ is described by a localization function $\mathcal{L}_{\rm enzyme} = \ell(r; R_{\rm s}, R_{\rm e}) \equiv \Theta(r - R_{\rm s})\Theta(R_{\rm e} - r)$ which is equal to one between its start radius $R_{\rm s}$ and its end radius $R_{\rm e}$ and zero elsewhere. Here, $\Theta(x)$ denotes the Heaviside step function. Under air-level CO_2 (10 μM cytosolic), Rubisco is localized to the pyrenoid matrix [15, 16], CAH3 is localized in the thylakoid tubules traversing the matrix [17], and LCIB is diffusely distributed in the stroma [18]. Thus, for the baseline model, we set $\mathcal{L}_{\rm Rbc} = \ell(r; 0, R_{\rm pyr})$, $\mathcal{L}_{\rm CAH3} = \ell(r; 0, R_{\rm pyr})$ and $\mathcal{L}_{\rm LCIB} = \ell(r; R_{\rm pyr}, R_{\rm chlor})$. To explore the effect of enzyme localization on PCCM performance (see Sec. III), we vary the start and end radii of the enzymes while maintaining a constant number of molecules.

Choice of parameters: For both CAH3 and LCIB, we estimate $K^C_{\rm m} = K^{H^-}_{\rm m} = 5$ mM as the values for a typical CA [19, 20]. To the best of our knowledge, the enzyme kinetics of CAH3 and LCIB have not been measured. Thus, we vary the $V^C_{\rm max}$ of both carbonic anhydrases in our model. In the baseline model, we choose the first order rate constants of CAH3 and LCIB to be 10^4 s^{-1} and 10^3 s^{-1}, respectively, which allows for the functioning of an effective PCCM (Fig. 2 and Supplementary Fig. 16). The rate constant for spontaneous conversion of CO_2 to HCO_3^- is set to be $k^C_{\rm sp} = 0.036$ s^{-1} [21].

For Chlamydomonas Rubisco, the half-saturation substrate concentrations are measured to be $K^C_{\rm m,Rbc} = 29$ μM and $K^O_{\rm m,Rbc} = 480$ μM [22, 23]. The oxygen concentration is assumed to be the same as that in aqueous solution in contact with air, $O = 230$ μM [24], since oxygen evolution only minimally affects the concentration of oxygen in the chloroplast. $V^C_{\rm max,Rbc}$ can be estimated either from the measured $k_{\rm cat}$ [22, 25] and concentration [26] of Rubisco or from saturating oxygen evolution measurements [16]. Both yield similar estimates of $V^C_{\rm max,Rbc} = 29$ mM/s. Note that we will use this set of kinetic parameters for Rubisco in our analysis unless otherwise specified (see for example Sec. VI E for discussion of plant Rubisco).

C. Thylakoid geometry

In vivo, the thylakoids enter the pyrenoid matrix through gaps in the starch sheath in the form of roughly cylindrical membrane tubules, and as they reach the center of the matrix (when $r \leqslant R_{\rm mesh} \approx 0.4$ μm) they become connected and form a reticulated meshwork [1]. These two geometries are modeled as follows:

Cylindrical tubules: For $r > R_{\rm mesh}$, we approximate the geometry of the thylakoids as cylindrical tubules exntending in the radial direction. We consider $N_{\rm tub}$ tubules, each having a constant radius of $a_{\rm tub}$. The volume fraction of these tubules at r is given by $f_{\rm v} = \frac{N_{\rm tub}\pi a^2_{\rm tub} dr}{4\pi r^2 dr} = \frac{N_{\rm tub} a^2_{\rm tub}}{4r^2}$. The infinitesimal surface area at radius r is given by $ds(r, dr) = N_{\rm tub}(2\pi a_{\rm tub})dr$, and we define $f_{\rm s} \equiv \frac{ds(r,dr)}{4\pi r^2 f_{\rm v} dr} = \frac{2}{a_{\rm tub}}$. We employ these functions $f_{\rm v}$ and $f_{\rm s}$ in our radially symmetric model of the PCCM.

Reticulated meshwork: For $r \leqslant R_{\rm mesh}$, we approximate the geometry of the thylakoids as a reticulated meshwork. To establish the necessary parameters for our radially symmetric model, we consider a cubic volume of L^3 containing a cubic mesh of thin tubules whose radius $a_{\rm tub}$ is much smaller than the intertubule mesh size ΔL. The volume fraction of the tubules in the meshwork can be estimated by $f_{\rm v} \approx 3(\frac{L^2}{\Delta L^2})\pi a^2_{\rm tub} L/L^3 = 3\pi a^2_{\rm tub}/(\Delta L)^2$, which is constant. The total surface area of tubules in the meshwork can be calculated as $3(\frac{L^2}{\Delta L^2})2\pi a_{\rm tub} L$. The ratio of this total surface area to the total volume of tubules in the meshwork $f_{\rm v} L^3$ yields $f_{\rm s} = \frac{2}{a_{\rm tub}}$.

Taken together, the geometric factors in Eqs. (S2 - S4) and (S7 - S9) are given by (Supplementary Fig. 2)

$$f_{\rm v}(r) = \begin{cases} \frac{N_{\rm tub} a_{\rm tub}^2}{4R_{\rm mesh}^2} & r \leqslant R_{\rm mesh} \\ \frac{N_{\rm tub} a_{\rm tub}^2}{4r^2} & r > R_{\rm mesh} \end{cases}, \text{ and } f_{\rm s}(r) \equiv \frac{2}{a_{\rm tub}}. \tag{S12}$$

Choice of parameters: For simplicity, in the baseline model we assume that the thylakoid tubules extend to the radius of the chloroplast $R_{\rm chlor}$. When tubules extend to a radius $R_{\rm tub}$ that is smaller than $R_{\rm chlor}$, we set $f_{\rm v} = 0$ and $f_{\rm s} = 0$ for $r > R_{\rm tub}$. Tubule geometric parameters are estimated from experimental images in [1] to be $N_{\rm tub} = 40$, and $a_{\rm tub} = 0.05$ μm.

D. Diffusion and transport of inorganic carbon across the thylakoid membranes

The diffusive flux of CO_2 across the thylakoid membrane from the thylakoid lumen to the matrix or stroma is calculated from

$$j^C_{\rm mem} = \begin{cases} \kappa^C (C_{\rm thy} - C_{\rm pyr}) & \text{for } r < R_{\rm pyr}, \text{ and} \\ \kappa^C (C_{\rm thy} - C_{\rm str}) & \text{for } r \geqslant R_{\rm pyr}, \end{cases} \tag{S13}$$

where κ^C denotes the permeability of thylakoid membranes to CO_2. The calculation of the diffusion flux takes the same form for H_2CO_3 and HCO_3^- with permeability coefficients κ^{H^0} and κ^{H^-}, respectively. In addition, Chlamydomonas cells possess thylakoid localized bestrophin-like anion channels that are essential to the PCCM [27, 28]. These proteins are the prime candidates for HCO_3^- channels to facilitate HCO_3^- diffusion across thylakoid membranes. Thus, we obtain the cross-membrane exchange flux of H_2CO_3 and HCO_3^- to be

$$j^H_{\rm mem} = \begin{cases} \kappa^{H^0}\left(\frac{1}{1+\eta_{\rm thy}} H_{\rm thy} - \frac{1}{1+\eta_{\rm pyr}} H_{\rm pyr}\right) + (\kappa^{H^-} + \kappa^{H^-}_{\rm thy})\left(\frac{\eta_{\rm thy}}{1+\eta_{\rm thy}} H_{\rm thy} - \frac{\eta_{\rm pyr}}{1+\eta_{\rm pyr}} H_{\rm pyr}\right) & \text{for } r < R_{\rm pyr} \\ \kappa^{H^0}\left(\frac{1}{1+\eta_{\rm thy}} H_{\rm thy} - \frac{1}{1+\eta_{\rm str}} H_{\rm str}\right) + (\kappa^{H^-} + \kappa^{H^-}_{\rm thy})\left(\frac{\eta_{\rm thy}}{1+\eta_{\rm thy}} H_{\rm thy} - \frac{\eta_{\rm str}}{1+\eta_{\rm str}} H_{\rm str}\right) & \text{for } r \geqslant R_{\rm pyr} \end{cases}, \tag{S14}$$

where $\kappa^{H^-}_{\rm thy}$ denotes the additional permeability of thylakoid membranes to HCO_3^- due to bestrophin-like channels.

Choice of parameters: We estimate $\kappa^C = 300$ μm/s and $\kappa^{H^-} = 0.05$ μm/s from previous measurements in diatoms [29]. We estimate $\kappa^{H^0} = 30$ μm/s assuming that H_2CO_3 has the same membrane permeability as formic acid (H_2CO_2) due to their identical charge and similar size [4, 30]. The bestrophin-like channels are not well characterized. Thus, we treat $\kappa^{H^-}_{\rm thy}$ as a variable. Note that we have assumed that these channels only allow the passage of HCO_3^- but not CO_2 and H_2CO_3. Such charge selectivity can presumably be achieved through electrostatic interactions of HCO_3^- with the channel [31, 32].

E. Diffusion and transport of inorganic carbon across the chloroplast envelope

We assume that the modeled chloroplast sits in an effectively infinite sea of cytosol which serves as a reservoir of inorganic carbon. For simplicity, we assume that the ratio of HCO_3^- concentration (denoted by $H^-_{\rm cyt}$) to CO_2 concentration (denoted by $C_{\rm cyt}$) is roughly 10 in the cytosol, which corresponds to the equilibrium at pH 7.1, close to the measured cytosolic pH [33].

CO_2 enters the chloroplast by diffusing passively through the chloroplast envelope, which sets the boundary condition

$$D^C_{\rm str} \frac{\partial C_{\rm str}}{\partial r}\Big|_{r=R_{\rm chlor}} = \kappa^C (C_{\rm cyt} - C_{\rm str}|_{r=R_{\rm chlor}}), \tag{S15}$$

where we have assumed that the permeability κ^C of the chloroplast membrane to CO_2 is the same as that of the thylakoid membranes.

Similarly, both H_2CO_3 and HCO_3^- can enter the chloroplast via passive diffusion with permeability coefficients κ^{H^-} and κ^{H^0}, respectively. In addition, Chlamydomonas cells can employ an active HCO_3^- uptake system, which consists of an ATP-binding cassette (ABC) transporter HLA3 at the plasma membrane and a formate/nitrite-transporter (FNT) LCIA at the chloroplast membrane to uptake HCO_3^- [34]. It is currently not known whether LCIA is a passive channel or a pump; therefore, in the model we first consider both possibilities, denoting by LCIA$^{\rm C}$ a passive channel

and denoting by LCIA^{P} an active pump. We introduce a chloroplast envelope HCO_3^- transporter with a rate $\kappa^{H^-}_{\text{chlor}}$ and a reversibility γ of inward HCO_3^- transport to represent the action of LCIA. Thus, the total influx of H_2CO_3 and HCO_3^- across the chloroplast envelope is

$$\kappa^{H^0}\Big(\frac{1}{1+\eta_{\text{cyt}}}H_{\text{cyt}} - \frac{1}{1+\eta_{\text{str}}}H_{\text{str}}|_{r=R_{\text{chlor}}}\Big) + \kappa^{H^-}\Big(\frac{\eta_{\text{cyt}}}{1+\eta_{\text{cyt}}}H_{\text{cyt}} - \frac{\eta_{\text{str}}}{1+\eta_{\text{str}}}H_{\text{str}}|_{r=R_{\text{chlor}}}\Big)$$
$$+ \kappa^{H^-}_{\text{chlor}}\Big(\frac{\eta_{\text{cyt}}}{1+\eta_{\text{cyt}}}H_{\text{cyt}} - \gamma\frac{\eta_{\text{str}}}{1+\eta_{\text{str}}}H_{\text{str}}|_{r=R_{\text{chlor}}}\Big) \equiv D^H_{\text{str}}\frac{\partial H_{\text{str}}}{\partial r}\Big|_{r=R_{\text{chlor}}}. \tag{S16}$$

Choice of parameters: The environmental CO_2 condition is specified by C_{cyt} in our model. In particular, we consider air-level CO_2 (400 ppm) which gives us $C_{\text{cyt}} = 10$ μM and very low CO_2 under which $C_{\text{cyt}} = 1$ μM [34]. For the chloroplast envelope HCO_3^- transporter, we vary its rate $\kappa^{H^-}_{\text{chlor}}$ and reversibility γ to explore possible PCCM performances (Fig. 3). Note that $\gamma = 0$ corresponds to completely unidirectional pumping while $\gamma = 1$ corresponds to facilitated diffusion by passive channels.

F. Starch sheath: a potential barrier to diffusion of inorganic carbon out of the pyrenoid matrix

In Chlamydomonas, the conditions that induce the PCCM also induce the formation of a starch sheath around the pyrenoid matrix [35]. This correlation has led to the speculation that the starch sheath could act as a barrier to slow the efflux of CO_2 (and other forms of inorganic carbon) from the pyrenoid matrix [35]. Here, we model the starch sheath as a thin membrane between the matrix and the stroma, with a permeability κ_{starch} to all forms of inorganic carbon. Thus, the boundary conditions at the pyrenoid radius R_{pyr} are given by

$$-D^C\frac{\partial C_{\text{pyr}}}{\partial r}\Big|_{r=R_{\text{pyr}}} = -D^C_{\text{str}}\frac{\partial C_{\text{str}}}{\partial r}\Big|_{r=R_{\text{pyr}}} = \kappa_{\text{starch}}\big(C_{\text{pyr}}|_{r=R_{\text{pyr}}} - C_{\text{str}}|_{r=R_{\text{pyr}}}\big),\ \text{and} \tag{S17a}$$
$$-D^H\frac{\partial H_{\text{pyr}}}{\partial r}\Big|_{r=R_{\text{pyr}}} = -D^H_{\text{str}}\frac{\partial H_{\text{str}}}{\partial r}\Big|_{r=R_{\text{pyr}}} = \kappa_{\text{starch}}\big(H_{\text{pyr}}|_{r=R_{\text{pyr}}} - H_{\text{str}}|_{r=R_{\text{pyr}}}\big). \tag{S17b}$$

Choice of parameters: Note that $\kappa_{\text{starch}} \to \infty$ corresponds to the case where the starch sheath plays no role in slowing the diffusion of inorganic carbon out of the pyrenoid matrix, while $\kappa_{\text{starch}} \to 0$ corresponds to the case where the starch sheath is completely impermeable to inorganic carbon, which can then enter or leave the pyrenoid only via the thylakoid tubules. In the absence of other types of diffusion barriers, we term the limit $\kappa_{\text{starch}} \to \infty$ "no diffusion barrier", and the limit $\kappa_{\text{starch}} \to 0$ "starch sheath" (see Fig. 2 and Supplementary Fig. 7). The performance of the PCCM for finite starch permeabilites between these two limits is shown in Supplementary Fig. 6. Note that for $\kappa_{\text{starch}} \lesssim \kappa^C = 10^{-4}$ m/s, CO_2 leakage from the pyrenoid occurs primarily via thylakoid tubules (Supplementary Fig. 6D). Thus, a starch sheath with a CO_2 permeability comparable to that of a typical membrane can be as effective a barrier as an impermeable starch sheath.

G. Thylakoid stacks: a potential barrier to slow diffusion of inorganic carbon in the stroma

Despite the proposed role of the starch sheath as a diffusion barrier preventing CO_2 efflux, mutants that are unable to synthesize starch still appear to grow similarly to wild-type Chlamydomonas at air-level CO_2 (10 μM cytosolic) [36, 37]. According to our model (see Fig. 2), however, the PCCM should be compromised in the absence of a barrier to CO_2 diffusion due to CO_2 leakage from the pyrenoid (see Sec. I H and Supplementary Fig. 3). To resolve this discrepancy, we speculate that the thylakoid stacks, which comprise layers of membranes surrounding the pyrenoid, could form another diffusion barrier to slow CO_2 (as well HCO_3^- and H_2CO_3) efflux.

For simplicity, we model the stroma packed with thylakoid stacks as a homogeneous compartment in which the diffusion of inorganic carbon is slowed. The effective diffusion coefficient D_{eff} depends on the geometry of the thylakoid stacks and the rate κ at which inorganic carbon species diffuse across thylakoid membranes. We model a realistic geometry of the thylakoid stacks *in silico* as follows (Supplementary Fig. 5): Thylakoids of luminal width d_h and membrane thickness d_t are stacked laterally with alternating large and small interthylakoid stroma spaces d_{l} and d_{s}. Thylakoid sheets possess wide and narrow gaps Δ_{w} and Δ_{n} through which inorganic carbon molecules can diffuse freely. The gaps are center-aligned and have a longitudinal repeat length of L_{rep}. Given the diffusion coefficient D in the thylakoid lumen and interthylakoid stroma space and the diffusion coefficient $D_{\text{mem}} = \kappa d_t$ through the thylakoid membranes, we numerically simulate 1D lateral diffusion across the *in silico* thylakoid stacks and obtain

$D_{\text{eff}}(D, D_{\text{mem}}) = D\phi(D_{\text{mem}}/D)$ where ϕ is the dimensionless calibration function shown in Supplementary Fig. 5.

In a model where we consider the thylakoid stacks as a diffusion barrier, we replace the diffusion coefficients in Eq. (S4, S9, S15, and S16) with $D^C_{\text{eff}} = D^C\phi(\frac{\kappa^C}{D^C/d_t})$ for CO_2 and with $D^H_{\text{eff}} = \frac{1}{1+\eta_{\text{str}}}D^{H^0}_{\text{eff}} + \frac{\eta_{\text{str}}}{1+\eta_{\text{str}}}D^{H^-}_{\text{eff}} \approx D^{H^-}\phi(\frac{\kappa^{H^-}+\kappa^{H^-}_{\text{thy}}}{D^{H^-}/d_t})$ for HCO_3^- and H_2CO_3.

Choice of parameters: We analyzed cryo-electron tomography images from [1, 38] and measured the following geometric parameters: $d_t = 5$ nm, $d_h = 10$ nm, $d_{\text{s}} = 4$ nm, $d_{\text{l}} = 40$ nm, $\Delta_{\text{n}} = 5.6$ nm, $\Delta_{\text{w}} = 50$ nm, and $L_{\text{rep}} = 800$ nm. In addition, we estimated the fraction of narrow gaps to be 40%. Note that PCCM performance for a model with both thylakoid stacks and an impermeable starch sheath is very similar to that of a model with only an impermeable starch sheath (Supplementary Fig. 8). In the absence of a starch sheath, the model dependence of PCCM performance on the strength of the diffusion barrier formed by the thylakoid stacks is shown in Supplementary Fig. 6.

H. Model discussion: CO_2 leakage

To illustrate the important issue of CO_2 leakage from the pyrenoid matrix in the absence of a diffusion barrier, we consider a simplified problem where CO_2 is produced at a flux of Q_i in the center of a sphere of radius R packed with Rubisco. CO_2 can either diffuse away or be consumed by Rubisco via linear kinetics with a first-order rate coefficient μ.

Reaction-diffusion kinetics. Assume that the diffusion coefficients of CO_2 molecules inside and outside the sphere are given by D_{in} and D_{out}, respectively. The reaction-diffusion kinetics can be described by

$$\begin{cases} D_{\text{in}}\frac{1}{r^2}\frac{\partial}{\partial r}\left(r^2\frac{\partial C}{\partial r}\right) - \mu c = 0 & \text{for } r \leqslant R, \text{ and} \\ D_{\text{out}}\frac{1}{r^2}\frac{\partial}{\partial r}\left(r^2\frac{\partial C}{\partial r}\right) = 0 & \text{for } r > R. \end{cases} \tag{S18}$$

The boundary conditions (B.C.s) are given by ① $C|_{r\to\infty} = C_\infty$, ② $C|_{r=R^+} = C|_{r=R^-}$, ③ $D_{\text{out}}\frac{\partial C}{\partial r}\big|_{r=R^+} = D_{\text{in}}\frac{\partial C}{\partial r}\big|_{r=R^-}$, and ④ $-4\pi r^2 D_{\text{in}}\frac{\partial C}{\partial r}\big|_{r=0} = Q_i$. Here, C_∞ denotes the concentration of CO_2 at infinity. Using B.C. ①, we obtain the solution for $r > R$, $C(r) = \mathcal{K}(R/r) + C_\infty$, where $\mathcal{K}$ is an undetermined constant. The general solution for $r \leqslant R$ can be written as $C = \mathcal{A}\sinh(\frac{r}{R_\mu})(R/r) + \mathcal{B}\cosh(\frac{r}{R_\mu})(R/r)$ where $\mathcal{A}$ and $\mathcal{B}$ are two constants, and $R_\mu = (D_{\text{in}}/\mu)^{1/2}$ denotes the typical length over which a CO_2 molecule can diffuse before being consumed by Rubisco. Next, we determine $\mathcal{A}, \mathcal{B}, \mathcal{K}$ using B.C.s ② - ④, which yield

$$② \Rightarrow \mathcal{A}\sinh(\tilde{R}) + \mathcal{B}\cosh(\tilde{R}) = c_\infty + \mathcal{K}, \tag{S19}$$

$$③ \Rightarrow \mathcal{A}\big[\cosh(\tilde{R})\tilde{R} - \sinh(\tilde{R})\big] + \mathcal{B}\big[\sinh(\tilde{R})\tilde{R} - \cosh(\tilde{R})\big] = -\epsilon_D\mathcal{K}, \text{ and} \tag{S20}$$

$$④ \Rightarrow \mathcal{B} = \frac{Q_i}{4\pi D_{\text{in}}R}, \tag{S21}$$

where we have defined two dimensionless variable $\epsilon_D = D_{\text{out}}/D_{\text{in}}$ and $\tilde{R} = R/R_\mu$. Equations (S19) and (S20) yield

$$\mathcal{A} = \frac{C_\infty\epsilon_D + \mathcal{B}\big[(1-\epsilon_D)\cosh(\tilde{R}) - \tilde{R}\sinh(\tilde{R})\big]}{\tilde{R}\cosh(\tilde{R}) - (1-\epsilon_D)\sinh(\tilde{R})}. \tag{S22}$$

Percent leakage. Within the simple model, how much CO_2, out of all the CO_2 produced at the center $r = 0$, diffuses away to infinity without being fixed by Rubisco? We compute the total leakage flux as $Q_R = -4\pi R^2 D_{\text{out}}\frac{\partial C}{\partial r}\big|_{r=R^+} = 4\pi D_{\text{out}}R\,\mathcal{K} = 4\pi D_{\text{out}}R\left[\mathcal{A}\sinh(\tilde{R}) + \mathcal{B}\cosh(\tilde{R}) - C_\infty\right]$. Introducing Eqs. (S21) and (S22) into the expression for Q_R, we obtain, in the low CO_2 limit $C_\infty \to 0$,

$$\frac{Q_R}{Q_i} \approx \frac{\epsilon_D\tilde{R}}{\tilde{R}\cosh(\tilde{R}) - (1-\epsilon_D)\sinh(\tilde{R})}. \tag{S23}$$

When the inside and outside have equal diffusivity, i.e., when $\epsilon_D = 1$, the right side of Eq. (S23) becomes $[\cosh(\tilde{R})]^{-1}$. When the radius R of the sphere of Rubisco is much smaller then the typical capture length R_μ, $\tilde{R} \ll 1$ and $Q_R/Q_i \approx 1$, i.e., almost all CO_2 diffuses away to infinity without being fixed by Rubisco. When $R \gg R_\mu$, Q_R/Q_i approaches zero as $\frac{1}{2}\exp(-\tilde{R})$ and CO_2 leakage is minimal. Choosing parameters relavant to the Chlamydomonas pyrenoid,

$R = R_{\mathrm{pyr}} \approx 1$ µm, $D_{\mathrm{in}} = D^C = 1.88 \times 10^3$ µm^2/s and $\mu \approx 676$/s (Supplementary Table 2), yields $\tilde{R} \approx 0.565$. Thus, as shown in Supplementary Fig. 3A, the CO_2 leakage percentage is 86% when there is no CO_2 diffusion barrier, i.e., when $\epsilon_D = 1$, and it decreases as ϵ_D drops, e.g., in the presence of thylakoid stacks acting to slow diffusion in the stroma.

Next, we assume $\epsilon_D = 1$ and consider how the percent CO_2 leakage varies with the size of the sphere filled with Rubisco. If the concentration of Rubisco is fixed, i.e., μ does not change with R, then the percent leakage decreases with increasing R (Supplementary Fig. 3B) — since R_μ is unchanged, $\tilde{R}$, and thus the probability of CO_2 being fixed, increases with R when there is more Rubiso. However, if the total number of Rubiscos is fixed, then $\mu \propto R^{-3}$ and therefore $\tilde{R} \propto R^{-1/2}$, so that the percent leakage actually decreases as R becomes smaller (Supplementary Fig. 3C), indicating that concentrating Rubisco in the pyrenoid matrix could help reduce CO_2 leakage.

I. Model discussion: Effect of different cytosolic Ci compositions

For simplicity, our model assumes a constant ratio of CO_2 to HCO_3^- in the cytosol, corresponding to the equilibrium ratio at pH 7.1 (see Sec. I). However, there is no known CA in the Chlamydomonas cytosol that could catalyze the otherwise slow interconversion between CO_2 and HCO_3^-. There is a periplasmic CA (CAH1), although mutants lacking this enzyme have no significant growth defect [39]. In addition, Chlamydomonas is known to employ transporters at the cell membrane to import Ci from the external environment to the cytosol [27, 40]. Thus, to explore the effect of different cytosolic Ci compositions on the efficacy of the PCCM, we varied C_{cyt} and H^-_{cyt} independently, assuming that HCO_3^- and H_2CO_3 remain in equilibrium at the cytosolic pH 7.1, and that the pH values of the different chloroplast compartments maintain their usual values under all conditions.

As shown in Supplementary Fig. 15, the efficacy of the PCCM in general increases with the the total concentrations of Ci in the cytosol. Notably, for a given total cytosolic Ci concentration, the efficacy of the two Ci uptake strategies varies with the ratio K_{C/H^-} of CO_2 to HCO_3^- in the cytosol, or the equivalent cytosolic pH, $\mathrm{pH}^{\mathrm{eqv}}_{\mathrm{cyt}}$, defined by $K_{C/H^-} \equiv 10^{\mathrm{pK_{eff}} - \mathrm{pH}^{\mathrm{eqv}}_{\mathrm{cyt}}}$. In particular, the efficacy of the passive CO_2 uptake strategy using LCIB decreases with increasing $\mathrm{pH}^{\mathrm{eqv}}_{\mathrm{cyt}}$ while the active HCO_3^- pumping strategy shows the opposite behavior. Future research could explore whether Chlamydomonas and other organisms employing a PCCM can coordinate the composition of the cytosolic pool of Ci with the Ci uptake strategies of the chloroplast.

II. EVALUATING THE PERFORMANCE OF THE PCCM

In this section, we discuss how to evaluate the functionality of the pyrenoid-based CCM. In Sec. II A we consider the ability to fix CO_2; in Sec. II B we consider the energetic cost to operate the PCCM; and in Sec. II C we attempt to integrate both aspects into a combined measure of PCCM performance. To simplify the notation, we denote by

$$I\big[R_\mathrm{s}, R_\mathrm{e}, f(r), g(r)\big] \equiv \int_{R_\mathrm{s}}^{R_\mathrm{e}} f(r) 4\pi r^2 g(r) dr \tag{S24}$$

the integral of an arbitrary function $f(r)$ from the starting radius R_s to the end radius R_e in spherical coordinates, where $g(r)$ denotes the geometric factor explicitly.

A. Normalized CO_2 fixation flux and net carbon fixation flux

The ability of the chloroplast to fix CO_2 can be quantified by comparing the CO_2 fixation flux through Rubisco to the maximal flux if Rubisco were fully saturated, i.e.,

$$\Phi_C \equiv \frac{I[0, R_\mathrm{chlor}, j_\mathrm{Rbc}, 1-f_\mathrm{v}]}{V^C_\mathrm{max,Rbc} I[0, R_\mathrm{chlor}, \mathcal{L}_\mathrm{Rbc}, 1-f_\mathrm{v}]}, \tag{S25}$$

which we term the Normalized CO_2 Fixation Flux (henceforth, NCFF). As a criterion for a "working" PCCM, we require NCFF > 0.5, i.e., local CO_2 concentration around Rubisco on average higher than the Rubisco K_m. When Rubisco becomes fully saturated, NCFF approaches 1.

As alluded to in Sec. I B, Rubisco also has oxygenase activity. The oxygenation flux can be calculated as

$$J_\mathrm{oxy} = I[0, R_\mathrm{chlor}, j_\mathrm{oxy}, 1-f_\mathrm{v}], \tag{S26}$$

where the oxygenation reaction is described by the Michaelis-Menten kinetics

$$j_\mathrm{oxy} = \frac{V^O_\mathrm{max,Rbc} O}{O + K^O_\mathrm{m,Rbc}(1 + \frac{C}{K^C_\mathrm{m,Rbc}})} \mathcal{L}_\mathrm{Rbc}. \tag{S27}$$

For every two oxygenation reactions, a carbon is lost in the form of CO_2 [41]. Thus, the net carbon fixation flux compared to its maximum is given by

$$\Psi_C \equiv \Phi_C - \Phi_O, \text{ where } \Phi_O \equiv \frac{1}{2}\left(\frac{J_\mathrm{oxy}}{V^C_\mathrm{max,Rbc} I[0, R_\mathrm{chlor}, \mathcal{L}_\mathrm{Rbc}, 1-f_\mathrm{v}]}\right). \tag{S28}$$

We term Ψ_C the Normalized Net Carbon Fixation Flux (henceforce, NNCFF). This metric will be used in Sec. II C when considering net carbon biomass growth.

B. Energetic cost of the PCCM

General formula. We follow the theoretical framework of nonequilibrium thermodynamics [42] to calculate the energetic cost of the PCCM. For a biochemical reaction out of equilibrium, the free-energy dissipation rate (per volume) can be calculated from [43–46]

$$\Delta \dot{W}_\mathrm{reaction} = (j_+ - j_-) \times \Big(RT \ln \frac{j_+}{j_-}\Big) \geqslant 0, \tag{S29}$$

where j_+ and j_- denote, respectively, the forward and backward reaction fluxes (in units of molar concentration per time), $(j_+ - j_-)$ is the net reaction flux, and $RT \ln \frac{j_+}{j_-}$ is the chemical potential difference between the reactants and products. Equation (S29) is equivalent to Crook's fluctuation theorem at nonequilibrium steady state, and can be applied to compute the free-energy dissipation for a wide range of nonequilibrium processes. For example, given a nonuniform concentration profile $c(r)$ in the radial direction, Eq. (S29) can be generalized to calculate the dissipation rate associated with the diffusion flux [42], which yields

$$\Delta \dot{W}_\mathrm{diffusion} = \Big(D \frac{\partial c}{\partial r}\Big) \times \Big(RT \frac{\partial \ln c}{\partial r}\Big), \tag{S30}$$

where D denotes the diffusion coefficient. Similarly, the dissipation rate (per area) of cross-membrane transport (e.g., Eq. S13) is given by

$$\Delta \dot{W}_{\mathrm{transport}} = \kappa(c_+ - \gamma c_-) \times \Big(RT \ln \frac{c_+}{\gamma c_-}\Big), \tag{S31}$$

where κ denotes the membrane transport rate, $c_\pm$ denotes the concentrations on either side of the membrane, and γ denotes the reversibility of the transport, so that $\gamma = 1$ corresponds to a bidirectional channel and $\gamma < 1$ corresponds to a pump.

Detailed derivation. In order to concentrate CO_2 locally around Rubisco at nonequilibrium steady state, free energy must be dissipated through various nonequilibrium processes. Here, we compute the total energetic cost of operating the PCCM, fixing CO_2, and making biomass. Our goal is to derive a general form for the total energetic cost that applies to an arbitrary localization of the PCCM proteins. To simplify notation, we will express the energy cost in units of RT and drop the term RT from the equations below.

Total energy consumption in the pyrenoid matrix: The energetic cost of nonequilibrium processes that occur in the pyrenoid matrix is computed from Eqs. (S29–S31), which yields

$$\begin{aligned}\dot{W}_{\mathrm{pyr}} = {} & I\big[0, R_{\mathrm{pyr}}, D^C(\partial_r C_{\mathrm{pyr}})(\partial_r \ln C_{\mathrm{pyr}}), 1-f_{\mathrm{v}}\big] + I\big[0, R_{\mathrm{pyr}}, D^H(\partial_r H^-_{\mathrm{pyr}})(\partial_r \ln H^-_{\mathrm{pyr}}), 1-f_{\mathrm{v}}\big] \\ & + I\big[0, R_{\mathrm{pyr}}, D^H(\partial_r H^0_{\mathrm{pyr}})(\partial_r \ln H^0_{\mathrm{pyr}}), 1-f_{\mathrm{v}}\big] + I\big[0, R_{\mathrm{pyr}}, (j_{\mathrm{sp}} + j_{\mathrm{LCIB}}) \ln \frac{C_{\mathrm{pyr}}}{K^{\mathrm{eq}}_{\mathrm{pyr}} H^-_{\mathrm{pyr}}}, 1-f_{\mathrm{v}}\big], \\ & + CO_2 \text{ fixation cost},\end{aligned} \tag{S32}$$

where the energy dissipated by diffusion is described by the first three terms and the energy dissipated by converting CO_2 to HCO_3^- is described by the fourth term (see Sec. I B for details). The energy cost of fixing CO_2 and converting it to biomass, termed "CO_2 fixation cost" in Eq. (S32), will be discussed below and further in Sec. II C. Recall that $K^{\mathrm{eq}} \equiv 10^{\mathrm{pK_{eff}}-\mathrm{pH}}$ is the equilibrium CO_2-to-HCO_3^- ratio in each compartment. We assume equal pH in the pyrenoid matrix and stroma, i.e., $K^{\mathrm{eq}}_{\mathrm{pyr}} = K^{\mathrm{eq}}_{\mathrm{str}}$. Integrating Eq. (S32) by parts and using the steady-state conditions Eqs. (S3, S8), we obtain

$$\begin{aligned}\dot{W}_{\mathrm{pyr}} = {} & \big(-J_C \ln C_{\mathrm{pyr}}\big)\big|_{R_{\mathrm{pyr}}} + \big(J_{H^-} \ln H^-_{\mathrm{pyr}}\big)\big|_{R_{\mathrm{pyr}}} + \big(J_{H^0} \ln H^0_{\mathrm{pyr}}\big)\big|_{R_{\mathrm{pyr}}} + I\big[0, R_{\mathrm{pyr}}, \kappa^C(C_{\mathrm{thy}} - C_{\mathrm{pyr}}) \ln C_{\mathrm{pyr}}, f_{\mathrm{s}} f_{\mathrm{v}}\big] \\ & + I\big[0, R_{\mathrm{pyr}}, (\kappa^{H^-} + \kappa^{H^-}_{\mathrm{thy}})(H^-_{\mathrm{thy}} - H^-_{\mathrm{pyr}}) \ln H^-_{\mathrm{pyr}}, f_{\mathrm{s}} f_{\mathrm{v}}\big] + I\big[0, R_{\mathrm{pyr}}, \kappa^{H^0}(H^0_{\mathrm{thy}} - H^0_{\mathrm{pyr}}) \ln H^0_{\mathrm{pyr}}, f_{\mathrm{s}} f_{\mathrm{v}}\big] \\ & - I\big[0, R_{\mathrm{pyr}}, (j_{\mathrm{sp}} + j_{\mathrm{LCIB}}) \ln K^{\mathrm{eq}}_{\mathrm{pyr}}, 1-f_{\mathrm{v}}\big] - I[0, R_{\mathrm{pyr}}, j_{\mathrm{Rbc}} \ln C_{\mathrm{pyr}}, 1-f_{\mathrm{v}}] + CO_2 \text{ fixation cost},\end{aligned} \tag{S33}$$

where we have defined[1] outward CO_2 flux $J_C \equiv -4\pi r^2(1-f_{\mathrm{v}}) D^C \frac{\partial C}{\partial r}$, inward HCO_3^- flux $J_{H^-} \equiv 4\pi r^2 (1-f_{\mathrm{v}}) D^H \frac{\partial H^-}{\partial r}$ and inward H_2CO_3 flux $J_{H^0} \equiv 4\pi r^2(1-f_{\mathrm{v}}) D^H \frac{\partial H^0}{\partial r}$, and we have used the boundary conditions $\frac{\partial C_{\mathrm{pyr}}}{\partial r}|_{r=0} = \frac{\partial H^0_{\mathrm{pyr}}}{\partial r}|_{r=0} = \frac{\partial H^-_{\mathrm{pyr}}}{\partial r}|_{r=0} = 0$.

Total energy consumption in the stroma: Similar to the calculation above, the energetic cost of nonequilibrium processes that occur in the stroma is given by

$$\begin{aligned}\dot{W}_{\mathrm{str}} = {} & I\big[R_{\mathrm{pyr}}, R_{\mathrm{chlor}}, D^C(\partial_r C_{\mathrm{str}})(\partial_r \ln C_{\mathrm{str}}), 1-f_{\mathrm{v}}\big] + I\big[R_{\mathrm{pyr}}, R_{\mathrm{chlor}}, D^H(\partial_r H^-_{\mathrm{str}})(\partial_r \ln H^-_{\mathrm{str}}), 1-f_{\mathrm{v}}\big] \\ & + I\big[R_{\mathrm{pyr}}, R_{\mathrm{chlor}}, D^H(\partial_r H^0_{\mathrm{str}})(\partial_r \ln H^0_{\mathrm{str}}), 1-f_{\mathrm{v}}\big] + I\big[R_{\mathrm{pyr}}, R_{\mathrm{chlor}}, (j_{\mathrm{sp}} + j_{\mathrm{LCIB}}) \ln \frac{C_{\mathrm{str}}}{K^{\mathrm{eq}}_{\mathrm{str}} H^-_{\mathrm{str}}}, 1-f_{\mathrm{v}}\big] \\ & + CO_2 \text{ fixation cost}.\end{aligned} \tag{S34}$$

[1] Note that these definitions also apply to the overall inward/outward flux at the chloroplast envelope $r = R_{\mathrm{chlor}}$, with $D^{C,H}$ replaced with $D^{C,H}_{\mathrm{str}}$.

Integrating Eq. (S34) by parts and using the steady-state conditions Eqs. (S4, S9), we obtain

$$\begin{aligned}\dot{W}_{\text{str}} &= \big(J_C \ln C_{\text{str}}\big)\big|_{R_{\text{pyr}}} - \big(J_C \ln C_{\text{str}}\big)\big|_{R_{\text{chlor}}} - \big(J_{H^-} \ln H^-_{\text{str}}\big)\big|_{R_{\text{pyr}}} + \big(J_{H^-} \ln H^-_{\text{str}}\big)\big|_{R_{\text{chlor}}}\\ &\quad - \big(J_{H^0} \ln H^0_{\text{str}}\big)\big|_{R_{\text{pyr}}} + \big(J_{H^0} \ln H^0_{\text{str}}\big)\big|_{R_{\text{chlor}}} - I\big[R_{\text{pyr}}, R_{\text{chlor}}, (j_{\text{sp}} + j_{\text{LCIB}}) \ln K^{\text{eq}}_{\text{str}}, 1 - f_{\text{v}}\big]\\ &\quad + I\big[R_{\text{pyr}}, R_{\text{chlor}}, \kappa^C (C_{\text{thy}} - C_{\text{str}}) \ln C_{\text{str}}, f_{\text{s}} f_{\text{v}}\big] + I\big[R_{\text{pyr}}, R_{\text{chlor}}, \kappa^{H^0} (H^0_{\text{thy}} - H^0_{\text{str}}) \ln H^0_{\text{str}}, f_{\text{s}} f_{\text{v}}\big]\\ &\quad + I\big[R_{\text{pyr}}, R_{\text{chlor}}, (\kappa^{H^-} + \kappa^{H^-}_{\text{thy}})(H^-_{\text{thy}} - H^-_{\text{str}}) \ln H^-_{\text{str}}, f_{\text{s}} f_{\text{v}}\big]\\ &\quad - I[R_{\text{pyr}}, R_{\text{chlor}}, j_{\text{Rbc}} \ln C_{\text{str}}, 1 - f_{\text{v}}] + \text{CO}_2 \text{ fixation cost.}\end{aligned} \tag{S35}$$

Total energy consumption in the thylakoid lumen: Similar to the calculations above, the energetic cost of nonequilibrium processes that occur in the thylakoid lumen including the tubules is given by

$$\begin{aligned}\dot{W}_{\text{thy}} &= I\big[0, R_{\text{chlor}}, D^C (\partial_r C_{\text{thy}})(\partial_r \ln C_{\text{thy}}), f_{\text{v}}\big] + I\big[0, R_{\text{chlor}}, D^H (\partial_r H^-_{\text{thy}})(\partial_r \ln H^-_{\text{thy}}), f_{\text{v}}\big]\\ &\quad + I\big[0, R_{\text{chlor}}, D^H (\partial_r H^0_{\text{str}})(\partial_r \ln H^0_{\text{str}}), f_{\text{v}}\big] + I\Big[0, R_{\text{chlor}}, (j_{\text{sp}} + j_{\text{CAH3}}) \ln \frac{C_{\text{thy}}}{K^{\text{eq}}_{\text{thy}} H^-_{\text{thy}}}, f_{\text{v}}\Big].\end{aligned} \tag{S36}$$

Integrating Eq. (S36) by parts and using the steady-state conditions Eqs. (S2, S4), we obtain

$$\begin{aligned}\dot{W}_{\text{thy}} &= -I\big[0, R_{\text{pyr}}, \kappa^C (C_{\text{thy}} - C_{\text{pyr}}) \ln C_{\text{thy}}, f_{\text{s}} f_{\text{v}}\big] - I\big[R_{\text{pyr}}, R_{\text{chlor}}, \kappa^C (C_{\text{thy}} - C_{\text{str}}) \ln C_{\text{thy}}, f_{\text{s}} f_{\text{v}}\big]\\ &\quad - I\big[0, R_{\text{pyr}}, (\kappa^{H^-} + \kappa^{H^-}_{\text{thy}})(H^-_{\text{thy}} - H^-_{\text{pyr}}) \ln H^-_{\text{thy}}, f_{\text{s}} f_{\text{v}}\big]\\ &\quad - I\big[R_{\text{pyr}}, R_{\text{chlor}}, (\kappa^{H^-} + \kappa^{H^-}_{\text{thy}})(H^-_{\text{thy}} - H^-_{\text{str}}) \ln H^-_{\text{thy}}, f_{\text{s}} f_{\text{v}}\big]\\ &\quad - I\big[0, R_{\text{pyr}}, \kappa^{H^0} (H^0_{\text{thy}} - H^0_{\text{pyr}}) \ln H^0_{\text{thy}}, f_{\text{s}} f_{\text{v}}\big] - I\big[R_{\text{pyr}}, R_{\text{chlor}}, \kappa^{H^0} (H^0_{\text{thy}} - H^0_{\text{str}}) \ln H^0_{\text{thy}}, f_{\text{s}} f_{\text{v}}\big]\\ &\quad - I\big[0, R_{\text{chlor}}, (j_{\text{sp}} + j_{\text{CAH3}}) \ln K^{\text{eq}}_{\text{thy}}, f_{\text{v}}\big],\end{aligned} \tag{S37}$$

where we have used the boundary conditions $\frac{\partial C_{\text{thy}}}{\partial r}|_{r=0} = \frac{\partial C_{\text{thy}}}{\partial r}|_{r=R_{\text{chlor}}} = \frac{\partial H^0_{\text{thy}}}{\partial r}|_{r=0} = \frac{\partial H^0_{\text{thy}}}{\partial r}|_{r=R_{\text{chlor}}} = \frac{\partial H^-_{\text{thy}}}{\partial r}|_{r=0} = \frac{\partial H^-_{\text{thy}}}{\partial r}|_{r=R_{\text{chlor}}} = 0$.

Total energy consumption of intercompartmental exchange: We consider three intercompartmental transport processes — exchange of carbon molecules (1) between the thylakoid lumen and the pyrenoid matrix/stroma, (2) between the matrix and the stroma, and (3) between the stroma and the cytosol. Their energetic costs are calculated as follows:

$$\begin{aligned}\dot{W}^{(1)}_{\text{intercomp}} &= I\Big[0, R_{\text{pyr}}, \kappa^C (C_{\text{thy}} - C_{\text{pyr}}) \ln \frac{C_{\text{thy}}}{C_{\text{pyr}}}, f_{\text{s}} f_{\text{v}}\Big] + I\Big[R_{\text{pyr}}, R_{\text{chlor}}, \kappa^C (C_{\text{thy}} - C_{\text{str}}) \ln \frac{C_{\text{thy}}}{C_{\text{str}}}, f_{\text{s}} f_{\text{v}}\Big]\\ &\quad + I\Big[0, R_{\text{pyr}}, (\kappa^{H^-} + \kappa^{H^-}_{\text{thy}})(H^-_{\text{thy}} - H^-_{\text{pyr}}) \ln \frac{H^-_{\text{thy}}}{H^-_{\text{pyr}}}, f_{\text{s}} f_{\text{v}}\Big]\\ &\quad + I\Big[R_{\text{pyr}}, R_{\text{chlor}}, (\kappa^{H^-} + \kappa^{H^-}_{\text{thy}})(H^-_{\text{thy}} - H^-_{\text{str}}) \ln \frac{H^-_{\text{thy}}}{H^-_{\text{str}}}, f_{\text{s}} f_{\text{v}}\Big]\\ &\quad + I\Big[0, R_{\text{pyr}}, \kappa^{H^0} (H^0_{\text{thy}} - H^0_{\text{pyr}}) \ln \frac{H^0_{\text{thy}}}{H^0_{\text{pyr}}}, f_{\text{s}} f_{\text{v}}\Big] + I\Big[R_{\text{pyr}}, R_{\text{chlor}}, \kappa^{H^0} (H^0_{\text{thy}} - H^0_{\text{str}}) \ln \frac{H^0_{\text{thy}}}{H^0_{\text{str}}}, f_{\text{s}} f_{\text{v}}\Big],\end{aligned} \tag{S38}$$

$$\dot{W}^{(2)}_{\text{intercomp}} = \Big(J_C \ln \frac{C_{\text{pyr}}}{C_{\text{str}}}\Big)\Big|_{R_{\text{pyr}}} + \Big(J_{H^-} \ln \frac{H^-_{\text{str}}}{H^-_{\text{pyr}}}\Big)\Big|_{R_{\text{pyr}}} + \Big(J_{H^0} \ln \frac{H^0_{\text{str}}}{H^0_{\text{pyr}}}\Big)\Big|_{R_{\text{pyr}}}, \text{ and} \tag{S39}$$

$$\begin{aligned}\dot{W}^{(3)}_{\text{intercomp}} &= \big(J_C\big)\big|_{R_{\text{chlor}}} \ln \frac{(C_{\text{str}})|_{R_{\text{chlor}}}}{C_{\text{cyt}}} + \big(J^{\text{diff}}_{H^-}\big)\big|_{R_{\text{chlor}}} \ln \frac{H^-_{\text{cyt}}}{(H^-_{\text{str}})|_{R_{\text{chlor}}}}\\ &\quad + \big(J^{\text{trans}}_{H^-}\big)\big|_{R_{\text{chlor}}} \ln \frac{H^-_{\text{cyt}}}{\gamma (H^-_{\text{str}})|_{R_{\text{chlor}}}} + \big(J_{H^0}\big)\big|_{R_{\text{chlor}}} \ln \frac{H^0_{\text{cyt}}}{(H^0_{\text{str}})|_{R_{\text{chlor}}}},\end{aligned} \tag{S40}$$

where $J_{H^-}^{\text{diff}}$ and $J_{H^-}^{\text{trans}}$ denote, respectively, the inward HCO_3^- flux via passive diffusion and via active HCO_3^- transporter (see Sec. I E for details).

Total energy consumption of reactions and transport of inorganic carbon molecules: Summing up all the terms in Eqs. (S33, S35, S37 & S38 – S40) yields the energetic cost

$$\begin{aligned}\dot{W}_{\text{carbon}} = &-\big(J_C\big)\big|_{R_{\text{chlor}}} \ln C_{\text{cyt}} + \big(J_{H^-}^{\text{diff}}\big)\big|_{R_{\text{chlor}}} \ln H_{\text{cyt}}^- + \big(J_{H^-}^{\text{trans}}\big)\big|_{R_{\text{chlor}}} \ln(\gamma^{-1} H_{\text{cyt}}^-) + \big(J_{H^0}\big)\big|_{R_{\text{chlor}}} \ln H_{\text{cyt}}^0 \\ &- J_{\text{str}}^{C\to H^-} \ln K_{\text{str}}^{\text{eq}} + J_{\text{thy}}^{H^-\to C} \ln K_{\text{thy}}^{\text{eq}} + \dot{W}_{\text{fixation}} \\ &- I[0, R_{\text{pyr}}, j_{\text{Rbc}} \ln C_{\text{pyr}}, 1-f_{\text{v}}] - I[R_{\text{pyr}}, R_{\text{chlor}}, j_{\text{Rbc}} \ln C_{\text{str}}, 1-f_{\text{v}}],\end{aligned} \tag{S41}$$

where $\dot{W}_{\text{fixation}}$ denotes the total cost of CO_2 fixation and biomass production from the local CO_2 concentration around Rubisco. We have defined the total conversion fluxes from CO_2 to HCO_3^- in the stroma and pyrenoid matrix as $J_{\text{str}}^{C\to H^-} \equiv I[0, R_{\text{chlor}}, j_{\text{sp}} + j_{\text{LCIB}}, 1-f_{\text{v}}]$, and the total conversion fluxes from HCO_3^- to CO_2 in the thylakoids as $J_{\text{thy}}^{H^-\to C} \equiv -I[0, R_{\text{chlor}}, j_{\text{sp}} + j_{\text{CAH3}}, f_{\text{v}}]$, respectively. The total CO_2 fixation flux is given by $J_{\text{Rbc}} = I[0, R_{\text{chlor}}, j_{\text{Rbc}}, 1-f_{\text{v}}]$. Note that $(J_C)|_{R_{\text{chlor}}}$ denotes the total CO_2 leakage flux out of the chloroplast and $(J_H)|_{R_{\text{chlor}}} \equiv (J_{H^-}^{\text{diff}})|_{R_{\text{chlor}}} + (J_{H^-}^{\text{trans}})|_{R_{\text{chlor}}} + (J_{H^0})|_{R_{\text{chlor}}}$ denotes the total inward flux of HCO_3^- and H_2CO_3. Thus, we obtain two global flux-balance conditions: (1) $(J_H)|_{R_{\text{chlor}}} = (J_C)|_{R_{\text{chlor}}} + J_{\text{Rbc}}$, since the carbon molecules entering the chloroplast will either be fixed by Rubisco or diffuse out again; and (2) $J_{\text{thy}}^{H^-\to C} = J_{\text{str}}^{C\to H^-} + (J_H)|_{R_{\text{chlor}}}$, since the HCO_3^- molecules converted to CO_2 in the thylakoids are either imported from outside the chloroplast or are recycling products in the stroma and pyrenoid matrix. Using these two relations to replace $(J_{H^-}^{\text{trans}})|_{R_{\text{chlor}}}$ and $J_{\text{thy}}^{H^-\to C}$ in Eq. (S41), we obtain

$$\begin{aligned}\dot{W}_{\text{carbon}} = &J_{\text{str}}^{C\to H^-} \ln \frac{K_{\text{thy}}^{\text{eq}}}{K_{\text{str}}^{\text{eq}}} + \big(J_C\big)\big|_{R_{\text{chlor}}} \ln \frac{\gamma^{-1} K_{\text{thy}}^{\text{eq}}}{K_{\text{str}}^{\text{eq}}} - \big(J_{H^-}^{\text{diff}}\big)\big|_{R_{\text{chlor}}} \ln \gamma^{-1} - \big(J_{H^0}\big)\big|_{R_{\text{chlor}}} \ln \frac{\gamma^{-1} H_{\text{cyt}}^0}{H_{\text{cyt}}^-} + \dot{W}_{\text{fixation}} \\ &+ J_{\text{Rbc}} \ln \frac{\gamma^{-1} K_{\text{thy}}^{\text{eq}}}{K_{\text{cyt}}^{\text{eq}}} + I\big[0, R_{\text{pyr}}, j_{\text{Rbc}} \ln \frac{C_{\text{cyt}}}{C_{\text{pyr}}}, 1-f_{\text{v}}\big] + I\big[R_{\text{pyr}}, R_{\text{chlor}}, j_{\text{Rbc}} \ln \frac{C_{\text{cyt}}}{C_{\text{str}}}, 1-f_{\text{v}}\big].\end{aligned} \tag{S42}$$

Note that in deriving Eq. (S42) we have assumed fast equilibrium between CO_2 and HCO_3^- in the cytosol, i.e., $C_{\text{cyt}}/H_{\text{cyt}}^- = K_{\text{cyt}}^{\text{eq}}$.

Total energy consumption of proton import: So far, we have only considered the energy cost associated with nonequilibrium reaction and transport of inorganic carbon molecules. Note that we have assumed constant pHs in different compartments and we have not yet explicitly considered the reaction and transport of protons. Nevertheless, protons are consumed by the reaction $HCO_3^- + H^+ \to CO_2 + H_2O$, and thus equal amounts of protons and HCO_3^- need to be taken into the chloroplast to maintain charge neutrality. Proton import from cytosolic pH to stromal pH yields a dissipation of

$$\dot{W}_{\text{proton}} = (J_{H^-})|_{R_{\text{chlor}}} \ln 10^{-\text{pH}_{\text{cyt}} + \text{pH}_{\text{str}}} = (J_{H^-})|_{R_{\text{chlor}}} \ln \frac{K_{\text{cyt}}^{\text{eq}}}{K_{\text{str}}^{\text{eq}}}. \tag{S43}$$

Total energy consumption of CO_2 fixation: Finally, we consider the energy cost in reactions of CO_2 fixation and biomass production. We choose air-level CO_2 $C_{\text{air}} = 10$ μM as our reference concentration and denote by ϵ_{b} the free-energy difference between CO_2 at C_{air} and in carbon biomass. Thus, the total energy cost of CO_2 fixation $\dot{W}_{\text{fixation}}$ in Eq. (S42) is given by

$$\dot{W}_{\text{fixation}} = J_{\text{Rbc}} \epsilon_{\text{b}} + I\big[0, R_{\text{pyr}}, j_{\text{Rbc}} \ln \frac{C_{\text{pyr}}}{C_{\text{air}}}, 1-f_{\text{v}}\big] + I\big[R_{\text{pyr}}, R_{\text{chlor}}, j_{\text{Rbc}} \ln \frac{C_{\text{str}}}{C_{\text{air}}}, 1-f_{\text{v}}\big], \tag{S44}$$

where the last two terms account for the free-energy difference of CO_2 between the local concentration at Rubisco and the reference concentration.

Summary and discussion. Combining Eq. (S42), (S43) and (S44), we obtain the total energetic cost (in units of RT) to be $\dot{W}_{\text{tot}} = \dot{W}_{\text{PCCM}} + \dot{W}_{\text{biomass}}$, where $\dot{W}_{\text{biomass}} = J_{\text{Rbc}} \epsilon_{\text{b}}$ denotes the cost of fixing CO_2 and making biomass

and $\dot{W}_{\rm PCCM}$ denotes the cost of operating the PCCM, which is given by

$$\dot{W}_{\rm PCCM} = J_{\rm str}^{C\to H^-} \ln\frac{K_{\rm thy}^{\rm eq}}{K_{\rm str}^{\rm eq}} + \left(J_{\rm C}\right)\big|_{R_{\rm chlor}} \ln\frac{\gamma^{-1}K_{\rm thy}^{\rm eq}}{K_{\rm str}^{\rm eq}} + J_{\rm Rbc}\Big(\ln\frac{\gamma^{-1}K_{\rm thy}^{\rm eq}}{K_{\rm str}^{\rm eq}} + \ln\frac{C_{\rm cyt}}{C_{\rm air}}\Big) - \left(J_{H^-}^{\rm diff}\right)\big|_{R_{\rm chlor}} \ln\gamma^{-1} - \left(J_{H^0}\right)\big|_{R_{\rm chlor}} \ln\Big(\frac{\gamma^{-1}K_{\rm cyt}^{\rm eq}}{K_{\rm str}^{\rm eq}} 10^{\rm pKa_1 - pH_{cyt}}\Big), \tag{S45}$$

where $\rm pKa_1 = 3.4$ denotes the first pKa of H_2CO_3 [4]. One can readily normalize Eq. (S45) by $J_{\rm Rbc}$ and use ATP hydrolysis energy $|\Delta G_{\rm ATP}| = 20.8RT$ (see [14] Box 13-1) to compute the equivalent ATP cost per CO_2 fixed to operate the PCCM, i.e., $\epsilon_C \equiv \dot{W}_{\rm PCCM}/J_{\rm Rbc}/20.8$.

Note that since the chloroplast membrane is quite impermeable to charged molecules like HCO_3^-, the influx of HCO_3^- via passive diffusion across the chloroplast envelope $\left(J_{H^-}^{\rm diff}\right)\big|_{R_{\rm chlor}}$ is negligible. Note also that all of the compartments in our model have pHs much larger than $\rm pKa_1$. Thus, the concentration of H_2CO_3 and its transport flux are also negligible compared to those of HCO_3^-. Neglecting the last two terms in Eq. (S45), we obtain a simplified (approximate) expression for the energetic cost of the PCCM at air-level CO_2 (10 μM cytosolic) in units of RT:

$$\dot{W}_{\rm PCCM} \approx J_{\rm str}^{C\to H^-} \ln\frac{K_{\rm str}^{\rm eq}}{K_{\rm thy}^{\rm eq}} + \left(J_{\rm C}\right)\big|_{R_{\rm chlor}} \ln\frac{\gamma^{-1}K_{\rm str}^{\rm eq}}{K_{\rm thy}^{\rm eq}} + J_{\rm Rbc}\ln\frac{\gamma^{-1}K_{\rm str}^{\rm eq}}{K_{\rm thy}^{\rm eq}}. \tag{S46}$$

The three terms in Eq. (S46) have clear physical meanings (Supplementary Fig. 13). The first term describes the energetic cost of LCIB participating in a futile cycle: HCO_3^- that is transported into the thylakoids is converted to CO_2, which then diffuses into the pyrenoid matrix and may diffuse back out into the stroma, where it will be recycled back to HCO_3^- by LCIB. For each cycle, the free energy dissipated $\sim \ln\frac{K_{\rm thy}^{\rm eq}}{K_{\rm str}^{\rm eq}} = \ln 10^{\rm pH_{str} - pH_{thy}}$ is equal to the energy needed to pump a proton from the stromal pH to the thylakoid pH. The second term describes the free energy wasted by CO_2 leakage, where the import of one molecule of HCO_3^- costs $\sim \ln\gamma^{-1}$ and pumping a proton into the thylakoid lumen to convert HCO_3^- to CO_2 costs $\sim \ln\frac{K_{\rm thy}^{\rm eq}}{K_{\rm str}^{\rm eq}}$. The third term describes the energetic cost of these same processes, but for those CO_2 molecules that are eventually fixed by Rubisco.

C. A combined measure of PCCM performance

As seen in Figs. 3 and 4, different PCCM performances can be achieved by varying enzyme activity and localization, as well as HCO_3^- transport rates. In this section, we seek to define a combined measure of PCCM performance that takes into account both the efficacy and the cost of concentrating CO_2. We then employ this combined measure to compare different PCCM strategies.

Energy and flux balance. For simplicity, we assume that the Chlamydomonas cell is partitioned into two parts, one with biomass $B_{\rm c}$ performing the PCCM and the other with biomass $(B_{\rm tot} - B_{\rm c})$ collecting light energy (Supplementary Fig. 28). The number of photons absorbed by the cell per unit time is assumed to be $\Gamma_{\rm ph} = \alpha(B_{\rm tot} - B_{\rm c})$ where α is a proportionality constant and depends on the incident light intensity. Part of the absorbed photons $\Gamma_{\rm m} = \nu B_{\rm tot}$ is used for maintenance [47], where ν denotes the number of maintenance photons per biomass. Thus, the total energy input that can be used for running the PCCM and synthesizing biomass is given by $P_{\rm tot} = \epsilon_{\rm ph}(\Gamma_{\rm ph} - \Gamma_{\rm m})$, where $\epsilon_{\rm ph}$ denotes the yield of chemical energy per absorbed photon.

For a particular PCCM strategy $\mathcal{S}$, the total CO_2 fixation flux by Rubisco is given by $\Gamma_C(\mathcal{S}) = \mu B_{\rm c}\Phi_C(\mathcal{S})$, where μ deonotes the maximum rate of CO_2 fixation per CCM biomass and Φ_C is the normalized CO_2 fixation flux. The total cost of concentrating CO_2 is given by $\epsilon_C(\mathcal{S})\Gamma_C(\mathcal{S})$, where ϵ_C denotes the energy cost per CO_2 fixed. However, some of the CO_2 fixed is then released through Rubisco's competitive oxygenation reaction. The total CO_2 flux lost through this reaction is given by $\Gamma_O(\mathcal{S}) = \mu B_{\rm c}\Phi_O(\mathcal{S})$ in which Φ_O is the normalized loss of CO_2 by oxygenation (Eq. S28), with a cost of ϵ_O per CO_2 lost. After fixing a net CO_2 flux of $\Gamma_C - \Gamma_O$ to organic carbon, the cell next converts this to biomass at a constant cost $\epsilon_{\rm b}$ per carbon. The total biomass carbon production flux is denoted by $\Gamma_{\rm b}$. Thus, the balance of energy and carbon flux requires that

$$\Gamma_C(\mathcal{S})\epsilon_C(\mathcal{S}) + \Gamma_{\rm b}\epsilon_{\rm b} + \Gamma_O(\mathcal{S})\epsilon_O \leqslant P_{\rm tot}, \text{ and} \tag{S47a}$$

$$\Gamma_{\rm b} \leqslant \Gamma_C - \Gamma_O. \tag{S47b}$$

The specific growth rate of the cell g can be computed as $g = \frac{\Gamma_b}{\chi_C B_{tot}}$, where χ_C is the fraction of carbon in biomass. Below, we discuss the optimal CCM strategy which maximizes g.

Optimal PCCM strategy.

Constrained optimization: Consider a cell with a fixed PCCM biomass fraction $f_c^0 = B_c^0/B_{tot}$. The specific growth rate depends on the choice of PCCM strategy as follows (Supplementary Fig. 28A): when Γ_C (and/or ϵ_C) is small, growth is limited by CO_2 fixation, i.e., $\Gamma_b = \Gamma_C(\mathcal{S}) - \Gamma_O(\mathcal{S}) = \mu\Psi_C(\mathcal{S})B_{tot}$ (Eq. S28), and the excess light energy is dissipated; when Γ_C (and/or ϵ_C) is large, growth rate becomes energy limited, which yields $\Gamma_b = \epsilon_b^{-1}\left[P_{tot} - \Gamma_C(\mathcal{S})\epsilon_C(\mathcal{S}) - \Gamma_O(\mathcal{S})\epsilon_O\right]$. Taken together, we obtain

$$g^0(\mathcal{S})\chi_C = \begin{cases} \mu\Psi_C(\mathcal{S})f_c^0 & \text{when } \mu f_c^0\left[\Phi_C(\epsilon_b+\epsilon_C)+\Phi_O(\epsilon_O-\epsilon_b)\right] \\ & \quad \le \epsilon_{ph}\left[\alpha(1-f_c^0)-\nu\right], \\ \frac{\epsilon_{ph}}{\epsilon_b}\left[\alpha(1-f_c^0)-\nu\right] - \frac{\epsilon_C}{\epsilon_b}\mu\Phi_C(\mathcal{S})f_c^0 - \frac{\epsilon_O}{\epsilon_b}\mu\Phi_O(\mathcal{S})f_c^0 & \text{otherwise.} \end{cases} \tag{S48}$$

The optimal PCCM strategy that maximizes the growth rate given a particular biomass partition is given by $\mathcal{S}^{\dagger,0} = \arg\max_{\mathcal{S}}\left(g^0(\mathcal{S})\right)$ (Supplementary Fig. 28B, F, and I).

Global optimization: Next we consider a cell that can adjust its PCCM biomass fraction $f_c = B_c/B_{tot}$ and the PCCM strategy to maximize growth. In this case, for a particular $\mathcal{S}$, the growth rate is maximized when both $\Gamma_b = \Gamma_C - \Gamma_O$ and $P_{tot} = \Gamma_b\epsilon_b + \Gamma_C\epsilon_C + \Gamma_O\epsilon_O$ are satisfied, which yields an optimal PCCM biomass fraction

$$f_c^*(\mathcal{S}) = \frac{\alpha-\nu}{\alpha + \mu\left[\Phi_C(\mathcal{S})\frac{\epsilon_b+\epsilon_C(\mathcal{S})}{\epsilon_{ph}} + \Phi_O(\mathcal{S})\frac{\epsilon_O-\epsilon_b}{\epsilon_{ph}}\right]}. \tag{S49}$$

Thus, the biomass growth rate is given by

$$g(\mathcal{S}) = \mu\Psi_C(\mathcal{S})f_c^*(\mathcal{S})\chi_C^{-1} = \chi_C^{-1}(\alpha-\nu)\Big(1-\frac{\Phi_O(\mathcal{S})}{\Phi_C(\mathcal{S})}\Big)\Big[\frac{\alpha}{\mu\Phi_C(\mathcal{S})} + \frac{\epsilon_b+\epsilon_C(\mathcal{S})}{\epsilon_{ph}} + \frac{\Phi_O(\mathcal{S})}{\Phi_C(\mathcal{S})}\Big(\frac{\epsilon_O-\epsilon_b}{\epsilon_{ph}}\Big)\Big]^{-1}. \tag{S50}$$

Optimizing g with respect to $\mathcal{S}$ yields the globally optimal PCCM strategy $\mathcal{S}^\dagger$ and growth rate $g^\dagger$, i.e., $S^\dagger = \arg\max_{\mathcal{S}}\left(g(\mathcal{S})\right)$ and $g^\dagger = g(\mathcal{S}^\dagger)$ (Supplementary Fig. 28C, G, and J).

Values of parameters. The reaction cost of biomass synthesis from CO_2 is $\epsilon_b \approx 1000$ kJ/C-mol biomass = 20 ATP/C biomass [48].[2] The energy cost of oxygenation per CO_2 lost can be computed to be $\epsilon_O \approx 26.4$ ATP [4, 49]. Note that ϵ_O is two times the energy cost of a oxygenation reaction due to the stoichiometry. The fraction of carbon in biomass is measured to be $\chi_C = 0.48$ g C/g biomass = 0.04 mol-C/g biomass [50].

The yield of chemical energy per photon ϵ_{ph} and the maintenance photon-flux coefficient ν are estimated from the chemostat experiments in [47]. Briefly, Chlamydomonas cells were grown in a chemostat at various dilution rates d, i.e., specific growth rates, and the corresponding numbers of absorbed photons per biomass per unit time R_{ph} were measured. R_{ph} was found to increase linearly with d, where the slope N_{ph} is the number of photons absorbed per synthesized biomass and the non-zero offset is the number of photons needed for maintenance, yielding $\nu = 6$ mmol/g biomass/h = 1.67 μmol/g biomass/s. N_{ph} was measured to be 0.8 mol photons/g biomass. Note that the energy requirement per biomass can be calculated by $N_{ph}\epsilon_{ph} = \epsilon_b\chi_C$. Plugging in the values of χ_C, ϵ_b, and N_{ph}, we obtain $\epsilon_{ph} = 1$ ATP/photon. Note that this number is consistent with an independent calculation based on the light reactions of photosynthesis. For linear electron flux (LEF), 8 photons transport 4 electrons, leading to formation of 2 NADPH (8.7 ATP equivalent energy) and the pumping of 12 protons (2.6 ATP equivalent energy) from the stroma to the thylakoid lumen [51, 52]. This gives a chemical energy yield of 1.4 ATP per photon. For cyclic electron flux (CEF), 1 photon drives the cyclic flux of 1 electron, leading to 2 protons pumped from the stroma to the thylakoid lumen, giving a chemical energy yield of 0.43 ATP per photon. In reality, it is likely that the cell uses a mixture of LEF and CEF to maintain a rapid stoichiometric balance of ATP and NADPH [53, 54], and thus ϵ_{ph} is expected to be within the range of 0.43 – 1.4 ATP per photon.[3]

---

[2] This value is for an electron donor that does not require reverse electron flow. The reaction cost is generally between 20 ATP and 60 ATP per biomass carbon.

[3] Here, we have chosen $\epsilon_b = 20$ ATP per carbon to obtain a simple estimate of $\epsilon_{ph} = 1$ ATP / photon.

As discussed above, α depends on the light intensity. Nevertheless, we can estimate the maximum α from light-saturating measurements of photosynthesis (e.g., [55]). In these experiments, cells were grown in medium supplemented with excess inorganic carbon (e.g., > 10 mM HCO_3^-). The measured photosynthesis activity first increases with increasing light intensity and eventually saturates for light intensity larger than $I_S \approx 200$ µmol photons $\cdot\, m^{-2} \cdot s^{-1}$. Thus, the saturating light absorption by a cell is given by $\Gamma_{ph,S} = I_S \times \Delta S \times (a^*_{Chl} C_{Chl} \ell)$, where ΔS is the effective illuminated area of a cell, ℓ is the (average) path length through the cell, a^*_{Chl} is the absorption coefficient (cross section) of chlorophyll and C_{Chl} is the concentration of chlorophyll. $\Gamma_{ph,S}$ can be rewritten as $\Gamma_{ph,S} = I_S \times a^*_{Chl} \times N_{Chl}$, where N_{Chl} is the total number of chlorophyll per cell.[4] Plugging in measurements from [56], $a^*_{Chl} = 10\ m^2/(g\ Chl)$ and $N_{Chl} = 1.5$ pg/cell yields $\Gamma_{ph,S} = 3 \times 10^{-9}$ µmol photons/cell/s. The maximum α can be readily calculated below after we estimate the biomass.

It has been measured that a typical Chlamydomonas cell has a total biomass of $B_{tot} = 43$ pg [56] and contains $B^{(prot)}_{tot} \approx 20$ pg protein in total [57]. The masses of the major CO_2 fixation proteins were measured to be the following [57]: Rubisco large subunit rbcL $B^{(prot)}_{rbcL} = 1.32$ pg, Rubisco small subunit RBCS $B^{(prot)}_{RBCS} = 0.33$ pg, pyrenoid linker protein EPYC1 $B^{(prot)}_{EPYC1} = 0.05$ pg, and Rubisco activase RCA $B^{(prot)}_{RCA} = 0.02$ pg.[5] Thus, we estimate the total biomass responsible for CO_2 fixation to be $B^{(prot)}_{c} \approx B^{(prot)}_{rbcL} + B^{(prot)}_{RBCS} + B^{(prot)}_{EPYC1} + B^{(prot)}_{RCA} = 1.72$ pg, which yields a PCCM biomass fraction $f^0_c \approx 0.086$. Introducing the values of f^0_c and B_{tot} into the relation $\alpha_S = \frac{\Gamma_{ph,S}}{B_{tot}(1-f^0_c)}$ yields the maximum rate of photon absorption $\alpha_S \approx 76$ µmol/g biomass/s. Finally, the maximum CO_2 fixation flux by Rubisco can be calculated from the turnover rate and the total number of active Rubisco catalytic sites [16, 26], which yields $\Gamma_{C,max} = 10^{-16}$ mol C/cell/s. Thus, $\mu = \frac{\Gamma_{C,max}}{B_{tot} f^0_c} \approx 27$ µmol/g biomass/s.

All the estimated parameter values from experiments are summarized in Supplementary Table 3. Guided by our theoretical framework, we wondered whether the cell optimizes its biomass fraction according to Eq. (S49). In particular, we consider the case where both CO_2 and light are in excess. In this case, there is no cost to concentrate CO_2, i.e., $\epsilon_C = 0$, there is no oxygenation $\Phi_O = 0$, and the normalized CO_2 fixation flux is $\Phi_C = 1$. Thus, using the parameter values discussed above, Eq. (S49) yields $f^*_c \approx 0.12$, larger than the estimated f^0_c. As shown in Supplementary Fig. 28B, this suggests that biomass growth of Chlamydomonas under saturating light conditions is predominantly limited by CO_2 fixation, and the growth rate is simply proportional to NNCFF (see Eq. (S28)).

[4] It can be seen here that the saturating energy input from light is proportional to the total biomass of chlorophyll, which justifies our expression $P_{tot} \propto (B_{tot} - B_c)$.

[5] Note that cells are grown in TAP medium under non-saturating light in the experiments in [57]. Nevertheless, the relative abundance of the four proteins above are in line with [16], and the absolute abundance of rbcL and RBSC are in line with [26]. Specifically, the total number of Rubisco can be calculated from its measured density in the pyrenoid matrix 0.63 mM in [26] and the volume of the pyrenoid 3.5 µm^3, which yields $N_{rbc} \approx 17.64$ amol assuming each Rubisco holoenzyme contains 8 large subunits and 8 small subunits. This agrees well with the reported value $N_{rbcL} = 25.2$ amol and $N_{RBCS} = 16.1$ amol in [57].

III. LOCALIZATION AND REGULATION OF KEY PCCM ENZYMES

Previous experiments have shown that the localization patterns and the expression or activity levels of key PCCM enzymes are regulated as Chlamydomonas cells respond to external CO_2 levels [16, 17, 58, 59]. One possibility is that such CO_2-dependent regulation contributes to the efficient functioning of the PCCM under different CO_2 conditions. In this section, we discuss how different localization patterns and activities of key PCCM enzymes impact PCCM performance in the modeled chloroplast.

A. Localization and regulation of CAH3

Previous experiments [17] suggest that CAH3 enzyme activity is upregulated under air-level CO_2 and that CAH3 is localized toward the intra-pyrenoid portion of the thylakoid tubules. In our baseline model, CAH3 serves as the immediate source of CO_2 that elevates the local CO_2 concentration around Rubisco. Thus, we hypothesize that certain activity and localization of CAH3 is needed only when external CO_2 concentration is low.

To test this hypothesis, we hold constant the total number of CAH3 and vary its localization. As shown in Supplementary Fig. 16B, G, and L, localizing CAH3 in the pyrenoid portion of the thylakoids indeed helps to concentrate more CO_2 in the vicinity of Rubisco under air-level CO_2 (10 μM cytosolic). Furthermore, such a localization pattern also reduces energy costs (Supplementary Fig. 16C, H, and M) by avoiding futile cycles when CAH3 and LCIB overlap radially. In the case of such an overlap, CO_2 produced by CAH3 can diffuse diffuse out of thylakoid tubules in the stroma, where it is then converted to HCO_3^- by LCIB. That HCO_3^- can then diffuse back into the tubules, where it is again converted to CO_2 by CAH3. Thus, the energetically least costly PCCM strategy that saturates Rubisco with CO_2 under air-level CO_2 is achieved when CAH3 is localized to the pyrenoid tubules and has a relatively high activity (Supplementary Fig. 16I and N). Finally, simulations verify that, indeed, as the external CO_2 level decreases, high activity and pyrenoid localization of CAH3 become required for a functioning PCCM, which is in line with the reported cellular regulation of CAH3 under air-level CO_2 [17].

B. Localization of LCIB

Under air-level CO_2 (10 μM cytosolic) when the passive CO_2 uptake strategy is used, for a chloroplast employing thylakoid stacks and no starch sheath, it is favorable for the PCCM to localize LCIB toward the chloroplast envelope (Supplementary Fig. 18; see also Sec. V C **Localization of LCIB**). In contrast, for a chloroplast employing an impermeable starch sheath, the localization patterns of LCIB minimally impact the performance of the PCCM (Supplementary Fig. 18). *In vivo*, LCIB is diffusely distributed in the Chlamydomonas chloroplast under air-level CO_2 [59]. We speculate that the passive CO_2 uptake strategy and a strong barrier formed by the starch sheath are employed by Chlamydomonas cells under such conditions.

LCIB puncta versus LCIB shell. Under very low CO_2 (1 μM cytosolic), LCIB is known to form puncta around the periphery of the starch sheath [58, 59]. This localization pattern has been suggested to contribute to PCCM performance [60]. Our model indicates that a punctate localization could be a strategy to avoid loss of inorganic carbon under very low CO_2: when LCIB is close to the envelope, HCO_3^- pumped across the chloroplast envelope is converted to CO_2, which could diffuse back out of the chloroplast immediately (Fig. 5). Furthermore, since the major role of LCIB is to recycle outward diffusing CO_2 and prevent leakage, we wonder whether forming LCIB puncta around the thylakoid tubules could benefit the PCCM more than forming a diffuse shell around the pyrenoid.

To estimate the difference between these two LCIB localization patterns, we consider an infinite cylindrical tubule of radius a_{tub} with a CO_2 concentration of C_i inside the tubule. We consider a case where CO_2 simply undergoes free diffusion outside the tubule, which is described by $\rho^{-1}\partial_\rho(\rho\partial_\rho C) = 0$ ($\rho \geqslant a_{\text{tub}}$) where ρ denotes the perpendicular distance from the central axis of the tubule. We denote by κ^C the membrane permeability to CO_2 on the side of the tubule. Thus, the boundary condition at $\rho = a_{\text{tub}}$ is given by $\kappa^C(C_i - C|_{\rho=a_{\text{tub}}}) = -D^C(\partial_\rho C)|_{\rho=a_{\text{tub}}}$ where D^C denotes the diffusion coefficient in the direction perpendicular to the axis of the tubule. We set the boundary condition away from the tubule to be $C|_{\rho=L} = C_\infty$. Here, we choose $L \approx 1$ μm to be the typical spacing between thylakoid tubules in the stroma, and we denote by C_∞ the concentration of CO_2 in the bulk stroma away from the thylakoid tubules. Thus, we obtain

$$C(\rho) = C_\infty - (C_i - C_\infty)\frac{\ln(\rho/L)}{\ln(L/a_{\text{tub}}) + D^C/(\kappa^C a_{\text{tub}})}. \tag{S51}$$

Introducing numbers listed in Supplementary Table 2, we find that

$$\frac{C|_{\rho=a_{\mathrm{tub}}} - C_\infty}{C_i - C_\infty} = \frac{\ln(L/a_{\mathrm{tub}})}{\ln(L/a_{\mathrm{tub}}) + D^C/(\kappa^C a_{\mathrm{tub}})} \approx 2.3\%. \tag{S52}$$

This suggests that the concentration gradient of CO_2 in the stroma from near to away from the thylakoid tubules is negligible. In this regard, the benefit of forming LCIB puncta around the thylakoid tubules to potentially capture CO_2 at a higher concentration is marginal compared to localizing LCIB in a shell around the pyrenoid.

IV. WELL-MIXED COMPARTMENT MODEL WITH AN IMPERMEABLE STARCH SHEATH

To gain more analytical insights into the two PCCM strategies illustrated in Fig. 3, we consider a simplification of the full reaction-diffusion model with an impermeable starch sheath. We assume that

1. Diffusion of CO_2 and HCO_3^- is fast in the pyrenoid matrix and stroma, and hence each of their concentrations can be described by a single number, $C_{\rm pyr}$, $C_{\rm str}$, $H^-_{\rm pyr}$, and $H^-_{\rm str}$, in the two compartments (Supplementary Fig. 21A).
2. HCO_3^- transport across the thylakoid membranes is fast. Thus, the thylakoid tubule concentrations of HCO_3^- inside and outside the pyrenoid can be approximated by $H^-_{\rm pyr}$ and $H^-_{\rm str}$, respectively.
3. CAH3 is fast and hence CO_2 and HCO_3^- are in fast equilibrium in the thylakoid tubules inside the pyrenoid where CAH3 is localized. Thus, the concentration of CO_2 can be described by a single number, $C_{\rm thy} = K^{\rm eq}_{\rm thy} H^-_{\rm pyr}$, in this intra-pyrenoid portion of the thylakoid tubules. Here, $K^{\rm eq}_{\rm thy} = 10^{0.1}$ is defined in Sec. I B.
4. The concentration of CO_2 in the thylakoid tubules near the chloroplast envelope approaches $C_{\rm str}$ (see for example Supplementary Fig. 7D).

We term this model the well-mixed compartment model, which has four unknowns $C_{\rm pyr}, C_{\rm thy}, C_{\rm str}$, and $H^-_{\rm str}$. In Sec. IV A we derive the algebraic governing equations. In Sec. IV B we consider two extremes of the various PCCM strategies, one employing passive uptake of CO_2 and one employing active pumping of HCO_3^-. In Sec. IV C we discuss the full PCCM strategy space under various external CO_2 conditions. Finally, in Sec. IV D we discuss the biophysical limits posed by the passive CO_2 uptake PCCM strategy.

A. Governing equations

The flux-balance conditions of inorganic carbon for the intra-pyrenoid portion of the chloroplast yield

$$\underbrace{2D^H a_{\rm tub} N_{\rm tub} (H^-_{\rm str} - C_{\rm thy}/K^{\rm eq}_{\rm thy})}_{\text{HCO}_3^- \text{ influx via thylakoid tubules}} = \underbrace{D^C (\pi a^2_{\rm tub}/L_{\rm thy}) N_{\rm tub} (C_{\rm thy} - C_{\rm str})}_{\text{CO}_2 \text{ efflux via thylakoid tubules}} + \underbrace{\Delta S_{\rm thy} \kappa^C (C_{\rm thy} - C_{\rm pyr})}_{\substack{\text{CO}_2 \text{ flux from the thylakoid} \\ \text{lumen to the pyrenoid matrix}}}, \text{ and} \tag{S53}$$

$$\Delta S_{\rm thy} \kappa^C (C_{\rm thy} - C_{\rm pyr}) = \underbrace{I[0, R_{\rm pyr}, 1, 1 - f_{\rm v}] V^C_{\rm max,Rbc} \frac{C_{\rm pyr}}{C_{\rm pyr} + K^{\rm eff}_{\rm m}}}_{\text{CO}_2 \text{ fixation flux by Rubisco}}. \tag{S54}$$

Here, $L_{\rm thy}$ is the typical diffusion length along the thylakoid tubules, which is obtained by fitting the well-mixed compartment model to the full simulation (see below and Supplementary Fig. 21). The diffusion flux of HCO_3^- through a circular hole of radius $a_{\rm tub}$ in an otherwise impermeable membrane is approximated by $2D^H a_{\rm tub} \Delta H$ [61]. $\Delta S_{\rm thy} = I[0, R_{\rm pyr}, f_{\rm s}, f_{\rm v}]$ denotes the total surface area of thylakoid membranes in the pyrenoid.

The flux balance of CO_2 in the chloroplast stroma is given by

$$D^C (\pi a^2_{\rm tub}/L_{\rm thy}) N_{\rm tub} (C_{\rm thy} - C_{\rm str}) = \underbrace{4\pi R^2_{\rm chlor} \kappa^C (C_{\rm str} - C_{\rm cyt})}_{\text{CO}_2 \text{ flux out of the chloroplast}} + \underbrace{I[R_{\rm pyr}, R_{\rm chlor}, 1, 1 - f_{\rm v}] \frac{V^C_{\rm max,LCIB}}{K^C_{\rm m}} (C_{\rm str} - K^{\rm eq}_{\rm str} H^-_{\rm str})}_{\text{conversion from CO}_2 \text{ to HCO}_3^-}. \tag{S55}$$

Here, we assume that LCIB is unsaturated and therefore its reaction is described by first-order kinetics.

Similarly, the flux balance of HCO_3^- in the stroma yields

$$2D^H a_{\rm tub} N_{\rm tub} (H^-_{\rm str} - C_{\rm thy}/K^{\rm eq}_{\rm thy}) = \underbrace{4\pi R^2_{\rm chlor} \kappa^{H^-}_{\rm chlor} (H^-_{\rm cyt} - \gamma H^-_{\rm str})}_{\text{HCO}_3^- \text{ uptake from the cytosol}} + I[R_{\rm pyr}, R_{\rm chlor}, 1, 1 - f_{\rm v}] \frac{V^C_{\rm max,LCIB}}{K^C_{\rm m}} (C_{\rm str} - K^{\rm eq}_{\rm str} H^-_{\rm str}), \tag{S56}$$

in which we assume that CO_2 and HCO_3^- are in fast equilibrium in the cytosol $H^-_{\rm cyt} = 10\ C_{\rm cyt}$ (see also Sec. I E).

Equations (S53–S56) can be written as

$$\alpha^{H^-}_{\rm thy} (H^-_{\rm str} - C_{\rm thy}/K^{\rm eq}_{\rm thy}) = \alpha^C_{\rm thy} (C_{\rm thy} - C_{\rm str}) + \sigma^C_{\rm thy} (C_{\rm thy} - C_{\rm pyr}), \tag{S57a}$$

$$\sigma^C_{\rm thy} (C_{\rm thy} - C_{\rm pyr}) = \frac{C_{\rm pyr}}{C_{\rm pyr} + K^{\rm eff}_{\rm m}}, \tag{S57b}$$

$$\alpha^C_{\rm thy}(C_{\rm thy}-C_{\rm str})=\sigma^C_{\rm chl}(C_{\rm str}-C_{\rm cyt})+\beta_{\rm LCIB}(C_{\rm str}-K^{\rm eq}_{\rm str}H^-_{\rm str}), \tag{S57c}$$

$$\alpha^{H^-}_{\rm thy}(H^-_{\rm str}-C_{\rm thy}/K^{\rm eq}_{\rm thy})=\sigma^{H^-}_{\rm chl}(10\ C_{\rm cyt}-\gamma H^-_{\rm str})+\beta_{\rm LCIB}(C_{\rm str}-K^{\rm eq}_{\rm str}H^-_{\rm str}), \tag{S57d}$$

where we have grouped the parameters into the following inverse concentrations: $\alpha^{H^-}_{\rm thy}=\frac{2D^H a_{\rm tub}N_{\rm tub}}{I[0,R_{\rm pyr},1,1-f_{\rm v}]V^C_{\rm max,Rbc}}=48.2\ {\rm mM}^{-1}$, $\alpha^C_{\rm thy}=\frac{D^C(\pi a^2_{\rm tub}/L_{\rm thy})N_{\rm tub}}{I[0,R_{\rm pyr},1,1-f_{\rm v}]V^C_{\rm max,Rbc}}=6.5\ {\rm mM}^{-1}$, $\sigma^C_{\rm thy}=\frac{\Delta S_{\rm thy}\kappa^C}{I[0,R_{\rm pyr},1,1-f_{\rm v}]V^C_{\rm max,Rbc}}=26.7\ {\rm mM}^{-1}$, $\sigma^C_{\rm chl}=\frac{4\pi R^2_{\rm chlor}\kappa^C}{I[0,R_{\rm pyr},1,1-f_{\rm v}]V^C_{\rm max,Rbc}}=389.5\ {\rm mM}^{-1}$, $\beta_{\rm LCIB}=\frac{I[R_{\rm pyr},R_{\rm chlor},1,1-f_{\rm v}]V^C_{\rm max,LCIB}/K^C_{\rm m}}{I[0,R_{\rm pyr},1,1-f_{\rm v}]V^C_{\rm max,Rbc}}$, $\sigma^{H^-}_{\rm chl}=\frac{4\pi R^2_{\rm chlor}\kappa^{H^-}_{\rm chlor}}{I[0,R_{\rm pyr},1,1-f_{\rm v}]V^C_{\rm max,Rbc}}$. The value of $\alpha^C_{\rm thy}$ is obtained by fitting the well-mixed compartment model to the full simulation (Supplementary Fig. 21B). All the notations and parameter values are summarized in Supplementary Table 4.

B. Two extreme PCCM strategies

Different PCCM strategies are parameterized by $\beta_{\rm LCIB}$, $\sigma^{H^-}_{\rm chl}$ and γ in the well-mixed compartment model. Here, we start by considering the extremes of the two PCCM strategies compared in Fig. 3.

Extreme 1: fast LCIB, no HCO_3^- transport across the chloroplast envelope. In this limit, $\beta_{\rm LCIB}\to\infty$ and $\sigma^{H^-}_{\rm chl}=0$, so CO_2 and HCO_3^- are in fast equilibrium in the stroma, i.e., $C_{\rm str}=K^{\rm eq}_{\rm str}H^-_{\rm str}$. Introducing these expressions into Eq. (S57), we obtain

$$\alpha^{H^-}_{\rm thy}(C_{\rm str}/K^{\rm eq}_{\rm str}-C_{\rm thy}/K^{\rm eq}_{\rm thy})=\alpha^C_{\rm thy}(C_{\rm thy}-C_{\rm str})+\sigma^C_{\rm thy}(C_{\rm thy}-C_{\rm pyr}),\ \text{and} \tag{S58a}$$

$$\sigma^C_{\rm thy}(C_{\rm thy}-C_{\rm pyr})=\frac{C_{\rm pyr}}{C_{\rm pyr}+K^{\rm eff}_{\rm m}}=\sigma^C_{\rm chl}(C_{\rm cyt}-C_{\rm str}). \tag{S58b}$$

Note that there are no undetermined parameters in Eq. (S58). Thus, the model predicts the ability to concentrate CO_2 (e.g., NCFF) under different CO_2 conditions if LCIB is the only inorganic carbon uptake system (Supplementary Fig. 22A). Note also that, consistent with full simulations (Fig. 3), NCFF is indeed larger than 0.5 under air-level CO_2 (10 μM cytosolic) and below 0.5 under very low CO_2 (1 μM cytosolic).

Extreme 2: no LCIB, fast HCO_3^- transport across the chloroplast envelope. In this limit, $\beta_{\rm LCIB}=0$ and $\sigma^{H^-}_{\rm chl}\to\infty$, and thus stroma HCO_3^- level is determined by $H^-_{\rm str}=\gamma^{-1}H^-_{\rm cyt}=\gamma^{-1}10\ C_{\rm cyt}$. Introducing these expressions into Eq. (S57), we obtain

$$\alpha^{H^-}_{\rm thy}(\gamma^{-1}10\ C_{\rm cyt}-C_{\rm thy}/K^{\rm eq}_{\rm thy})=\alpha^C_{\rm thy}(C_{\rm thy}-C_{\rm str})+\sigma^C_{\rm thy}(C_{\rm thy}-C_{\rm pyr}), \tag{S59a}$$

$$\sigma^C_{\rm thy}(C_{\rm thy}-C_{\rm pyr})=\frac{C_{\rm pyr}}{C_{\rm pyr}+K^{\rm eff}_{\rm m}},\ \text{and} \tag{S59b}$$

$$\alpha^C_{\rm thy}(C_{\rm thy}-C_{\rm str})=\sigma^C_{\rm chl}(C_{\rm str}-C_{\rm cyt}). \tag{S59c}$$

As shown in Supplementary Fig. 22B, active HCO_3^- transporters of reversibility $\gamma=0.2$ behave similarly to the passive CO_2 uptake strategy exmploying fast LCIB (see **Extreme 1** above), and active HCO_3^- transporters of smaller reversibility (e.g., $\gamma\leqslant 0.05$) enable NCFF >0.5 under both air-level CO_2 and very low CO_2 conditions.

C. Feasible PCCM strategies under varying CO_2 conditions

We next consider an arbitrary PCCM strategy in the well-mixed compartment model. Similar to the full reaction-diffusion model, we can compute the overall reaction fluxes of the PCCM enzymes as well as the fluxes of inorganic carbon transport between compartments to assess the performance, i.e., NCFF and energy cost, of a given PCCM strategy (Supplementary Fig. 23A and B). The results of the two PCCM strategies are in line with Fig. 3. In addition, Supplementary Fig. 23A and B show that, in the presence of fast HCO_3^- pumping across the chloroplast transport, increasing LCIB activity only minimally affects NCFF but reduces the energy cost by recycling outward diffusing CO_2.

Solving Eq. (S57) under different external CO_2 levels yields the lower bound $C^*_{\rm cyt}$ below which NCFF <0.5 (Supplementary Fig. 23C and D). For facilitated diffusion of HCO_3^- across the chloroplast envelope ($\gamma=1$), the lowest $C^*_{\rm cyt}$ is achieved by employing LCIB to catalyze passive CO_2 uptake with no investment in HCO_3^- transport

across the chloroplast envelope (termed "the passive CO_2 uptake strategy", see Sec. IV D). For the PCCM to function at even lower CO_2 levels, active HCO_3^- pumping ($\gamma < 0.2$) across the chloroplast envelope needs to be turned on. This suggests that the cell might activate different sets of PCCM components depending on the environmental CO_2 conditions. The least costly functional PCCM strategy of the well-mixed compartment model under varying external CO_2 conditions is found to be the sequential activation of LCIB followed by chloroplast envelope HCO_3^- pumps (Supplementary Fig. 23E), which is also the case in the full simulation (Supplementary Fig. 24).

D. Limits of the passive CO_2 uptake strategy

What are the relevant biophysical limits for the PCCM? For the passive CO_2 uptake strategy, where there is no HCO_3^- transport across the chloroplast envelope, the efficacy of the PCCM is limited by the influx of CO_2 which depends on the external CO_2 concentration and the size of the chloroplast. For such strategy, the overall flux balance of carbon takes the same form as Eq. (S58b), since the influx must be in the form of CO_2 and must be equal to the total CO_2 fixation flux. Since C_{str} cannot be negative, we obtain

$$\mathrm{NCFF} = \frac{C_{\mathrm{pyr}}}{C_{\mathrm{pyr}} + K_{\mathrm{m}}^{\mathrm{eff}}} = \sigma_{\mathrm{chl}}^{C}(C_{\mathrm{cyt}} - C_{\mathrm{str}}) \leqslant \sigma_{\mathrm{chl}}^{C} C_{\mathrm{cyt}}, \tag{S60}$$

which yields the lowest external CO_2 concentration, $C_{\mathrm{cyt}}^{*} \sim (\sigma_{\mathrm{chl}}^{C})^{-1} = 2.6$ μM, above which Rubisco could in principle reach saturation (see Supplementary Fig. 22A).

In the rough estimation above, we have assumed that the total CO_2 influx is not diffusion-limited, but rather limited by the chloroplast membrane permeability to CO_2 ($\sigma_{\mathrm{chl}}^{C}$ or κ^{C}). However, the passive CO_2 uptake strategy will fail at a higher CO_2 concentration if the size of the chloroplast is increased — eventually CO_2 absorption becomes diffusion-limited with a flux $\propto R_{\mathrm{chlor}}$ while the saturating CO_2 fixation flux scales as R_{chlor}^{3}. Thus, we wonder whether this diffusion-limited absorption sets a relevant limit to the size of the chloroplast, assuming that the Chlamydomonas cell employs such a PCCM strategy under air-level CO_2 (10 μM cytosolic).

To calculate CO_2 absorption flux by a chloroplast of arbitrary size, we consider free diffusion of CO_2 outside the chloroplast, described by $r^{-2}\partial_r(r^2\partial_r C) = 0$ in which C denotes the concentration of CO_2 and r denotes the distance from the chloroplast center. Assuming complete absorption, the boundary conditions are given by $D^{C}(\partial_r C)|_{r=R_{\mathrm{chlor}}} = \kappa^{C} C|_{r=R_{\mathrm{chlor}}}$ and $C|_{r=\infty} = C_{\mathrm{cyt}}$. Solving the differential equation yields $C(r) = C_{\mathrm{cyt}} - \frac{C_{\mathrm{cyt}}}{1+D^{C}/(\kappa^{C} R_{\mathrm{chlor}})}\frac{R_{\mathrm{chlor}}}{r}$. Thus, the total CO_2 uptake flux is given by $4\pi R_{\mathrm{chlor}}^{2}\kappa^{C} C|_{r=R_{\mathrm{chlor}}} = \frac{4\pi R_{\mathrm{chlor}} D^{C} C_{\mathrm{cyt}}}{1+D^{C}/(\kappa^{C} R_{\mathrm{chlor}})}$. Setting this equal to the saturating Rubisco fixation flux $\frac{4}{3}\pi R_{\mathrm{pyr}}^{3} V_{\mathrm{max,Rbc}}^{C} = \frac{4}{3}\pi(\chi R_{\mathrm{chlor}})^{3} V_{\mathrm{max,Rbc}}^{C}$ yields the upper size limit R_{chlor}^{*} below which the passive CO_2 uptake strategy is functional. Here, we have assumed that the pyrenoid varies its size in proportion to the chloroplast with a proportionality constant χ, while maintaining the same Rubisco density. Plugging in $D^{C} = 1.88 \times 10^{3}$ μm/s, $C_{\mathrm{cyt}} = 10$ μM, $\kappa^{C} = 300$ μm/s and $V_{\mathrm{max,Rbc}}^{C} = 29$ mM/s (Supplementary Table 2), we obtain $R_{\mathrm{chlor}}^{*} = 5.9$ μm for $\chi = 0.3$, and $R_{\mathrm{chlor}}^{*} = 3.2$ μm for $\chi = 0.4$. These values are consistent with the typical dimensions of algal chloroplasts, which are typically smaller than 5 μm, suggesting a relevant biophysical size limit imposed by CO_2 uptake.

V. WELL-MIXED COMPARTMENT MODEL WITH THYLAKOID STACKS

As seen in Fig. 2 and Supplementary Fig. 6, a model with thylakoid stacks slowing inorganic carbon diffusion in the stroma behaves qualitatively differently from a model with a starch sheath. In particular, the former shows a nonmonotonic dependence of NCFF on LCIB activity, while in the latter model NCFF increases monotonically with LCIB activity, plateauing at high LCIB rates. To better understand the differences, we aim here to analyze the diffusion barrier formed by the thylakoid stacks using the framework of the well-mixed compartment model as above (Sec. IV A). In Sec. V A, we derive the typical diffusion length scale of CO_2 in the stroma, which depends on LCIB activity. In Sec. V B, we derive the governing equations of a simplified model. In Sec. V C, we discuss the consequences of the thylakoid stacks acting as a diffusion barrier.

A. Diffusion length scale in the stroma

Consider the reaction-diffusion kinetics of CO_2 in the stroma. For simplicity, we ignore the effect of the thylakoid tubules since they have a negligible volume fraction compared to the stroma (Supplementary Fig. 2). We further approximate the reaction kinetics of LCIB to be linear and ignore the spontaneous interconversion between CO_2 and HCO_3^-. Thus, Eq. (S4) becomes

$$\frac{a_\beta^2}{r^2}\frac{\partial}{\partial r}\left(r^2\frac{\partial C_{\rm str}}{\partial r}\right) - \left(C_{\rm str} - K_{\rm str}^{\rm eq}H_{\rm str}^-\right) = 0, \tag{S61}$$

where $a_\beta = \left(\frac{D_{\rm eff}^C}{V_{\rm max,LCIB}^C/K_{\rm m}^C}\right)^{1/2}$ denotes the typical distance over which a CO_2 molecule can diffuse in the stroma before it is converted to HCO_3^- by LCIB. Assuming that $H_{\rm str}^-$ is a constant in the stroma, we consider the boundary condition problem with $C_{\rm str}(r = R_{\rm pyr}) = C_*$ and $C_{\rm str}(r = R_{\rm chlor}) = C_0$ and compute the diffusive flux of CO_2 across $r = R_{\rm pyr}$. In general, one can solve the exact concentration profile from Eq. (S61) and use the expression $-D_{\rm eff}^C\frac{\partial C}{\partial r}$ to compute the flux. Here, for simplicity, we focus on the limit where either the stroma domain is large enough or LCIB is fast enough that $C_0 \approx K_{\rm str}^{\rm eq}H_{\rm str}^-$ (see Sec. V C for justification). In this limit, we obtain

$$C_{\rm str}(r) = C_0 - (C_* - C_0)\frac{R_{\rm pyr}}{r}\frac{\sinh(r/a_\beta - R_{\rm chlor}/a_\beta)}{\sinh(\Delta R_{\rm str}/a_\beta)}, \tag{S62}$$

where $\Delta R_{\rm str} = R_{\rm chlor} - R_{\rm pyr}$. Thus, the diffusive flux of CO_2 across $r = R_{\rm pyr}$ can be written as $-D_{\rm eff}^C\frac{\partial C_{\rm str}}{\partial r}|_{r=R_{\rm pyr}} = D_{\rm eff}^C\frac{C_*-C_0}{L_{\rm str}}$ where the diffusion length scale in the stroma $L_{\rm str}$ is given by

$$L_{\rm str}^{-1} = R_{\rm pyr}^{-1} + a_\beta^{-1}\coth(\Delta R_{\rm str}/a_\beta). \tag{S63}$$

Note that $L_{\rm str}$ is dependent on LCIB activity via a_β: when LCIB activity is low, i.e., $a_\beta \to \infty$, $L_{\rm str} = \Delta R_{\rm str}R_{\rm pyr}/R_{\rm chlor}$ which is determined solely by the chloroplast geometry; when LCIB activity is high, i.e., $a_\beta \to 0$, $L_{\rm str} \approx a_\beta$ which is determined solely by LCIB activity.

B. Flux-balance equations

Here, we derive the algebraic equations of flux-balance conditions in a chloroplast with thylakoid stacks, similar to the derivations of Eq. (S57) in Sec. IV A. Because the diffusion coefficient of inorganic carbon in the stroma is smaller than that in the pyrenoid matrix due to the modeled thylakoid stacks, the concentration profiles are flatter in the matrix (Supplementary Fig. 7A). Thus, we approximate the CO_2 concentration in the pyrenoid matrix as constant, denoted by a single number $C_{\rm pyr}$. Similarly, we assume that the CO_2 concentration in the thylakoid tubules inside the pyrenoid can be approximated by another constant, $C_{\rm thy}$. CO_2 concentrations in the thylakoids outside the pyrenoid and in the stroma decrease from $C_{\rm thy}$ and $C_{\rm pyr}$, respectively, with increasing radius, and they approach the same plateau $C_{\rm str}$ near the chloroplast envelope. For simplicity, we assume that the concentrations of HCO_3^- in all the compartments can be approximated by the same constant H^-. [6] Below, we consider the passive CO_2 uptake strategy

---

[6] This becomes a good approximation when the HCO_3^- transport rate across the thylakoid membrane $\kappa_{\rm thy}^{H^-}$ is fast. For $\kappa_{\rm thy}^{H^-} \sim \kappa^C$, one could introduce the same set of variables as the CO_2 concentrations, i.e., $H_{\rm pyr}^-$, $H_{\rm thy}^-$, $H_{\rm str}^-$, and consider flux-balance equations similar to Eqs. (S66) and (S67). However, the assumptions that $\kappa_{\rm thy}^{H^-}$ is fast is not essential to understanding the nonmonotonic dependence of NCFF on LCIB activity.

where there is no HCO_3^- transport across the chloroplast envelope.

The overall flux balance of HCO_3^- in the chloroplast is given by

$$I[R_{\rm pyr}, R_{\rm chlor}, 1, 1-f_{\rm v}]\frac{V^C_{\rm max,LCIB}}{K^C_{\rm m}}(C_{\rm str} - K^{\rm eq}_{\rm str}H^-) = I[0, R_{\rm pyr}, 1, f_{\rm v}]\frac{V^C_{\rm max,CAH3}}{K^C_{\rm m}}(K^{\rm eq}_{\rm thy}H^- - C_{\rm thy}), \tag{S64}$$

where the left-hand side and the right-hand side of the equation denote the total HCO_3^- production by LCIB and the total HCO_3^- consumption by CAH3, respectively. Here, we have again assumed linear kinetics of LCIB and CAH3 (see also Sec. IV A).

The overall flux balance of CO_2 in the chloroplast dictates that the flux of CO_2 diffusing into the chloroplast is equal to the flux of CO_2 consumed by Rubisco, i.e.,

$$4\pi R^2_{\rm chlor}\kappa^C(C_{\rm cyt} - C_{\rm str}) = I[0, R_{\rm pyr}, 1, 1-f_{\rm v}]V^C_{\rm max,Rbc}\frac{C_{\rm pyr}}{C_{\rm pyr} + K^{\rm eff}_{\rm m}}. \tag{S65}$$

The flux balance of CO_2 in the pyrenoid matrix yields

$$\Delta S_{\rm thy}\kappa^C(C_{\rm thy} - C_{\rm pyr}) = I[0, R_{\rm pyr}, 1, 1-f_{\rm v}]V^C_{\rm max,Rbc}\frac{C_{\rm pyr}}{C_{\rm pyr} + K^{\rm eff}_{\rm m}} + 4\pi R^2_{\rm pyr}(1 - f_{\rm v}|_{r=R_{\rm pyr}})D^C_{\rm eff}\frac{C_{\rm pyr} - C_{\rm str}}{L_{\rm str}}, \tag{S66}$$

since the CO_2 molecules entering the matrix from the thylakoid lumen (denoted by the left-hand side) must either be fixed by Rubisco (denoted by first term on the right-hand side) or diffuse back into the stroma (denoted by the second term on the right-hand side). Here, we have used results from Sec. V A to express the flux of CO_2 diffusing from the pyrenoid matrix to the stroma. The diffusion length scale in the stroma $L_{\rm str}$ is given by Eq. (S63).

Finally, the flux balance of CO_2 in the thylakoid tubules inside the pyrenoid yields

$$I[0, R_{\rm pyr}, 1, f_{\rm v}]\frac{V^C_{\rm max,CAH3}}{K^C_{\rm m}}(K^{\rm eq}_{\rm thy}H^- - C_{\rm thy}) = \Delta S_{\rm thy}\kappa^C(C_{\rm thy} - C_{\rm pyr}) + D^C(\pi a^2_{\rm tub}/L_{\rm thy})N_{\rm tub}(C_{\rm thy} - C_{\rm str}), \tag{S67}$$

since CO_2 produced by CAH3 (denoted by the left-hand side) must either diffuse into the pyrenoid matrix from the thylakoid lumen (denoted by first term on the right-hand side) or diffuse away via the thylakoid tubules (denoted by second term on the right-hand side; see also Eq. (S53)).

Equations (S64–S67) can be written as

$$\beta_{\rm LCIB}(C_{\rm str} - K^{\rm eq}_{\rm str}H^-) = \beta_{\rm CAH3}(K^{\rm eq}_{\rm thy}H^- - C_{\rm thy}), \tag{S68a}$$

$$\sigma^C_{\rm chl}(C_{\rm cyt} - C_{\rm str}) = \frac{C_{\rm pyr}}{C_{\rm pyr} + K^{\rm eff}_{\rm m}}, \tag{S68b}$$

$$\sigma^C_{\rm thy}(C_{\rm thy} - C_{\rm pyr}) = \frac{C_{\rm pyr}}{C_{\rm pyr} + K^{\rm eff}_{\rm m}} + \alpha^C_{\rm str}(C_{\rm pyr} - C_{\rm str}), \tag{S68c}$$

$$\beta_{\rm CAH3}(K^{\rm eq}_{\rm thy}H^- - C_{\rm thy}) = \sigma^C_{\rm chl}(C_{\rm thy} - C_{\rm pyr}) + \alpha^C_{\rm str}(C_{\rm pyr} - C_{\rm str}), \tag{S68d}$$

where we have again grouped the parameters into the following inverse concentrations (see also Eqs. (S57a–S57d) and the surrounding text for definitions). Here, we define $\beta_{\rm CAH3} \equiv \frac{I[0,R_{\rm pyr},1,f_{\rm v}]V^C_{\rm max,CAH3}/K^C_{\rm m}}{I[0,R_{\rm pyr},1,1-f_{\rm v}]V^C_{\rm max,Rbc}} = 22.2\ {\rm mM}^{-1}$ and $\alpha^C_{\rm str} \equiv \frac{4\pi R^2_{\rm pyr}(1-f_{\rm v}|_{r=R_{\rm pyr}})D^C_{\rm eff}/L_{\rm str}}{I[0,R_{\rm pyr},1,1-f_{\rm v}]V^C_{\rm max,Rbc}}$, which is dependent on LCIB activity. Although much simplified, Eqs. (S68a – S68d) capture many key features of the full simulation. When LCIB activity is low, CAH3 is the dominant carbonic anhydrase in the chloroplast. Since CAH3 works in the thylakoid tubules at pH 6, the HCO_3^- to CO_2 ratios in both the stroma and inner thylakoids are close to the equilibrium ratio at pH 6 (Supplementary Fig. 9A). As LCIB activity increases, more CO_2 diffusing into the chloroplast is converted to HCO_3^- by LCIB. Thus, the concentration of stromal HCO_3^- increases and the HCO_3^- to CO_2 ratio in the stroma approaches the equilibrium ratio at pH 8 (Supplementary Fig. 9A and B). Consequently, more HCO_3^- can diffuse into the thylakoid tubules and become available for the conversion of CO_2 by CAH3, leading to an increase of the CO_2 concentration in the thylakoid tubules inside the pyrenoid (Supplementary Fig. 9B). Eq. (S63) also indicates that the transport coefficient $\alpha^C_{\rm str} \propto L^{-1}_{\rm str}$ increases with LCIB activity. Specifically, in the limit of fast LCIB, $\alpha^C_{\rm str} \sim L^{-1}_{\rm str} \approx a^{-1}_\beta \sim (V^C_{\rm max,LCIB}/K^C_{\rm m})^{1/2}$ (Sec. V A and Supplementary Fig. 9C).

C. Results and discussion

Optimal LCIB activity. To gain more analytical insight into the nonmonotonic dependence of NCFF on LCIB when thylakoid stacks are employed, we ignore the only nonlinear term $\frac{C_{\rm pyr}}{C_{\rm pyr}+K_{\rm m}^{\rm eff}}$ in Eq. (S68) and proceed with $C_{\rm str} = C_{\rm cyt}$. Note that this is a reasonable simplification since the neglected term is smaller than 1 while the coefficients α, β and σ are much larger than 1 (Supplementary Table 4). Solving the simplified linear equations, we obtain

$$C_{\rm pyr} = C_{\rm cyt} + \frac{\sigma^C_{\rm thy}}{\sigma^C_{\rm thy} + \alpha^C_{\rm str}}\big(C_{\rm thy} - C_{\rm cyt}\big), \tag{S69}$$

where $C_{\rm thy}$ is given by

$$C_{\rm thy} = C_{\rm cyt} + \frac{\beta_{\rm CAH3}}{\beta_{\rm CAH3} + \alpha}\big(K^{\rm eq}_{\rm thy}H^- - C_{\rm cyt}\big) \text{ in which } H^- = \frac{\beta_{\rm LCIB} + \alpha^*}{K^{\rm eq}_{\rm str}\beta_{\rm LCIB} + K^{\rm eq}_{\rm thy}\alpha^*}C_{\rm cyt}. \tag{S70}$$

To simplify the expression, we have defined $\alpha \equiv \alpha^C_{\rm thy} + \frac{\alpha^C_{\rm str}\sigma^C_{\rm thy}}{\alpha^C_{\rm str}+\sigma^C_{\rm thy}}$ and $\alpha^* \equiv \frac{\alpha\beta_{\rm CAH3}}{\alpha+\beta_{\rm CAH3}}$. The two competing effects of increasing LCIB activity on concentrating CO_2 are apparent in Eqs. (S69) and (S70). Since $K^{\rm eq}_{\rm str} \ll K^{\rm eq}_{\rm thy}$, Eq. (S70) indicates the benefit of increasing LCIB activity ($\beta_{\rm LCIB}$): HCO_3^- concentration in the chloroplast increases, leading to a high level of CO_2 ($C_{\rm thy}$) in the thylakoid tubules inside the pyrenoid. The concentration of CO_2 is decreased as it diffuses from the thylakoid lumen into the pyrenoid matrix and then back out into the stroma, and the concentration drop from one compartment to another is inversely proportional to the transport coefficients $\alpha^C_{\rm str}$ and $\sigma^C_{\rm thy}$ (inset of Supplementary Fig. 9C). Equation (S69) describes the detrimental contribution of increasing LCIB activity: LCIB converts CO_2 diffusing out of the pyrenoid matrix to HCO_3^-, leading to an increase of $\alpha^C_{\rm str}$ while $\sigma^C_{\rm thy}$ remains constant; thus, the concentration drop from the pyrenoid matrix to the stroma becomes smaller as LCIB activity increases, implying a lower level of CO_2 in the matrix. Together, the two competing effects result in the existence of an intermediate LCIB activity at which NCFF and pyrenoid matrix CO_2 concentration reach the maximum (Supplementary Figs. 7E and 9D). This behavior is unique to the model with thylakoid stacks and no starch sheath since the path of CO_2 diffusing from the pyrenoid matrix to stroma is blocked when a starch sheath is present.

Introducing parameter values into Eqs. (S69) and (S70) allows us to determine quantitatively where the optimal LCIB activity occurs. For the beneficial contribution Eq. (S70), the factor $\frac{\beta_{\rm LCIB}+\alpha^*}{K^{\rm eq}_{\rm str}\beta_{\rm LCIB}+K^{\rm eq}_{\rm thy}\alpha^*}$ reaches half its maximum when $\beta_{\rm LCIB} = (\frac{K^{\rm eq}_{\rm thy}}{K^{\rm eq}_{\rm str}} - 2)\alpha^* = 98\alpha^*$, which corresponds to an LCIB first-order rate constant of $k_{\frac{1}{2}} = 980\ {\rm s}^{-1}$. Similarly, for the detrimental contribution Eq. (S69), setting the factor $\frac{\sigma^C_{\rm thy}}{\sigma^C_{\rm thy}+\alpha^C_{\rm str}}$ to 1/2 yields an LCIB first-order rate constant of 1328 ${\rm s}^{-1}$. These numbers are in good agreement with the optimal LCIB rate of about $10^3\ {\rm s}^{-1}$ found in the full simulation (Supplementary Fig. 9D). Note also that the detrimental contribution becomes important only for high LCIB rates when the beneficial contribution saturates. Thus, in deriving the expression Eq. (S63) for $L_{\rm str}$, which is directly related to the detrimental contribution, we have made the assumption of fast LCIB (see Sec. V A).

Optimal membrane permeability to CO_2. If the permeability to CO_2 of thylakoid membranes κ^C is the same for both the thylakoid tubules and stacks, Eq. (S69) also suggests the competing effects of increasing κ^C: when κ^C is small, i.e., when $\sigma^C_{\rm thy} \propto \kappa^C$ is small, it is difficult for the CO_2 produced by CAH3 in the thylakoid tubules to diffuse into the pyrenoid matrix, leading to a low level of CO_2 therein; when κ^C is large, the diffusion coefficient of CO_2 in the stroma becomes large (Supplementary Fig. 5), leading to a large CO_2 leakage flux out of the pyrenoid matrix and impairing CCM performance (Supplementary Fig. 3). Thus, the theory predicts an intermediate κ^C that maximizes NCFF or pyrenoid matrix CO_2 concentration. This prediction is verified by varying κ^C in the reaction-diffusion model described in Sec. I A (Supplementary Fig. 10A and B). Results obtained from the simplified model Eq. (S68) show good agreement with the full simulation (Supplementary Fig. 10C – E).

Localization of LCIB. In deriving Eq. (S63), we have considered diffuse LCIB in the stroma. Intuitively, moving LCIB away from the boundary between the pyrenoid matrix and stroma will increase the diffusion length scale $L_{\rm str}$ as defined in Sec. V A. We wondered whether this would qualitatively affect the nonmonotonic dependence of NCFF on increasing LCIB activity. To address this question, we consider a scenario in which LCIB range from $R_{\rm pyr} + \delta$ to $R_{\rm chlor}$ where δ denotes the radial width of the LCIB-free buffer zone. Free diffusion is described by $r^{-2}\partial_r(r^2\partial_r C_{\rm str}) = 0$

between $R_{\rm pyr}$ and $R_{\rm pyr}+\delta$, and the reaction-diffusion equation $a_\beta^2 r^{-2}\partial_r(r^2\partial_r C_{\rm str}) - (C_{\rm str} - C_0) = 0$ applies for $r > R_{\rm pyr}+\delta$. Here, for the same reason as above, we have focused on the case where LCIB is fast (i.e., a_β is small) and we consider boundary conditions $C_{\rm str}(r = R_{\rm pyr}) = C_*$ and $C_{\rm str}(r=\infty) = C_0$.[7] Thus, the general solution of $C_{\rm str}$ is given by

$$C_{\rm str}(r) = \begin{cases} C_* + \mathcal{A}(1/r - 1/R_{\rm pyr}) & \text{for } R_{\rm pyr} \leqslant r \leqslant R_{\rm pyr}+\delta, \text{ and} \\ C_0 + \mathcal{B}(a_\beta/r)\exp(-r/a_\beta) & \text{for } r \geqslant R_{\rm pyr}+\delta, \end{cases} \tag{S71}$$

where $\mathcal{A}$ and $\mathcal{B}$ are constants determined by the boundary conditions at $r = R_{\rm pyr}+\delta$, i.e., the concentration $C_{\rm str}$ and its derivative must be continuous, which yields

$$C_* - \mathcal{A}\frac{\delta}{(R_{\rm pyr}+\delta)R_{\rm pyr}} = C_0 + \mathcal{B}\frac{a_\beta}{R_{\rm pyr}+\delta}\exp\Big(-\frac{R_{\rm pyr}+\delta}{a_\beta}\Big), \text{ and} \tag{S72a}$$

$$\mathcal{A}(R_{\rm pyr}+\delta)^{-2} = \mathcal{B}(R_{\rm pyr}+\delta)^{-1}\exp\Big(-\frac{R_{\rm pyr}+\delta}{a_\beta}\Big)\Big(1+\frac{a_\beta}{R_{\rm pyr}+\delta}\Big). \tag{S72b}$$

Solving Eq. (S72), we obtain $\mathcal{A} = (C_* - C_0)\big[R_{\rm pyr}^{-1} - (R_{\rm pyr}+\delta+a_\beta)^{-1}\big]^{-1}$. The diffusive flux of CO_2 across $r = R_{\rm pyr}$ is given by $-D_{\rm eff}^C\partial_r C_{\rm str}|_{r=R_{\rm pyr}} = -D_{\rm eff}^C\mathcal{A}/R_{\rm pyr}^2$. Introducing the expression for $\mathcal{A}$ and rewriting the flux in terms of $L_{\rm str}$ as $D_{\rm eff}^C\frac{C_*-C_0}{L_{\rm str}}$, we obtain

$$L_{\rm str}^{-1} = R_{\rm pyr}^{-1} + (\delta + a_\beta)^{-1}. \tag{S73}$$

Recall that when LCIB is diffuse, $L_{\rm str}\approx a_\beta$ is solely determined by LCIB activity and it approaches 0 in the fast LCIB limit. In contrast, when LCIB is moved away from the pyrenoid by a distance δ, $L_{\rm str}\approx \delta R_{\rm pyr}/(\delta+R_{\rm pyr})$ is determined by the geometry and it approaches some finite value in the fast LCIB limit. Denote by $a_{\frac{1}{2}} = (D_{\rm eff}^C/k_{\frac{1}{2}})^{1/2}$ where $k_{\frac{1}{2}}$ is again the first-order rate constant of LCIB above which the beneficial contribution starts to saturate (see **Optimal LCIB activity** above). Equation (S73) also indicates that when $\delta \gtrsim a_{\frac{1}{2}}$, the detrimental contribution of LCIB at high activity will be masked by the LCIB-free slow diffusion buffer zone. Thus, this analysis predicts a critical width of $\delta^* \approx a_{\frac{1}{2}} \sim (D_{\rm eff}^C)^{1/2}$, below which NCFF has nonmonotonic dependence on LCIB activity and above which NCFF increases monotonically with LCIB activity. This is indeed the case in the full simulation and the dependence of δ^* on the stroma diffusivity $D_{\rm eff}^C$ is verified (Supplementary Fig. 11).

Estimating parameter values in the full simulation. To test whether the above calculations in the simplified model also hold true for the reaction-diffusion model (Sec. I A), we estimate the corresponding parameter values in the full simulation as follows. First, the concentrations of CO_2 and HCO_3^- are estimated by their average in each compartment in the full simulation (Supplementary Fig. 9A and B). Second, according to the definitions of the transport coefficients $\alpha_{\rm str}^C$ and $\sigma_{\rm thy}^C$, we divide the diffusive fluxes of CO_2 by the (average) concentration difference between compartments to estimate the values of $\alpha_{\rm str}^C$ and $\sigma_{\rm thy}^C$ in the simulation (Supplementary Figs. 9 and 10). Finally, we estimate $a_{\frac{1}{2}} = (D_{\rm eff}/10^3\ {\rm s}^{-1})^{1/2}$ (Supplementary Fig. 11) since $k_{\frac{1}{2}}$ is roughly $10^3\ {\rm s}^{-1}$ under various stroma diffusivity conditions.

[7] Using this boundary condition at a finite radius $C_{\rm str}(r = R_{\rm chlor}) = C_0$ yields $L_{\rm str} = \frac{\delta R_{\rm pyr}}{\delta+R_{\rm pyr}} + \frac{a_\beta}{(1+\delta/R_{\rm pyr})^2}\frac{\sinh(x)}{\cosh(x)+\sinh(x)a_\beta/(R_{\rm pyr}+\delta)}$ where $x = (R_{\rm chlor} - R_{\rm pyr} - \delta)/a_\beta$. We recover Eq. (S73) when $a_\beta \ll R_{\rm chlor} - R_{\rm pyr} - \delta$.

VI. SUPPLEMENTARY DISCUSSION

A. Alternative thylakoid morphologies

In our model of the PCCM, the thylakoid tubules that traverse the pyrenoid and converge in the pyrenoid center are responsible for delivering stromal HCO_3^- to the pyrenoid, where this HCO_3^- can be converted to CO_2 by CAH3. This particular architecture of the thylakoid tubules is based on that seen in Chlamydomonas , but it is not necessary to the PCCM. Based on the key insights yielded from our model, we hypothesize that other thylakoid morphologies could also support the functioning of an efficient PCCM, as long as (1) HCO_3^- can diffuse across the thylakoid membranes, (2) the thylakoid lumen maintains a low pH, and (3) there is thylakoid CA in the vicinity of the pyrenoid matrix to convert HCO_3^- to CO_2. To test this hypothesis, we study models with distinct thylakoid morphologies as described below. As a proof of concept, we only consider the passive CO_2 uptake strategy under air-level CO_2, i.e., $C_{\rm cyt} = 10$ μM, and we ignore the spontaneous conversion between HCO_3^- and CO_2 for simplicity. Parameters are the same as in the baseline Chlamydomonas PCCM model.

We start by considering a model with a shell of thylakoid sheet surrounding the pyrenoid matrix (see Supplementary Fig. 25, model 1). Indeed, this thylakoid morphology has been found in other algae [62]. The equations describing the steady-state flux balance of CO_2, HCO_3^-, and H_2CO_3 are:

$$\begin{cases} r^{-2}\partial_r(r^2 D^C \partial_r C_{\rm pyr}) - j_{\rm Rbc} = 0 \\ r^{-2}\partial_r(r^2 D^H \partial_r H_{\rm pyr}) = 0 \end{cases} \qquad \text{for } r \leqslant R_{\rm pyr}, \tag{S74a}$$

$$\begin{cases} r^{-2}\partial_r(r^2 D^C \partial_r C_{\rm thy}) - j_{\rm CAH3} = 0 \\ r^{-2}\partial_r(r^2 D^H \partial_r H_{\rm thy}) + j_{\rm CAH3} = 0 = 0 \end{cases} \qquad \text{for } R_{\rm pyr} \leqslant r \leqslant R_{\rm pyr} + \Delta R_{\rm thy}, \tag{S74b}$$

$$\begin{cases} r^{-2}\partial_r(r^2 D^C \partial_r C_{\rm str}) - j_{\rm LCIB} = 0 \\ r^{-2}\partial_r(r^2 D^H \partial_r H_{\rm str}) + j_{\rm LCIB} = 0 = 0 \end{cases} \qquad \text{for } R_{\rm pyr} + \Delta R_{\rm thy} \leqslant r \leqslant R_{\rm chlor}. \tag{S74c}$$

Here, we assume that the PCCM enzymes are distributed diffusely in their respective compartments and that their reaction fluxes $j_{\rm X}$ take the same form as in the Chlamydomonas PCCM model (Eqs. (S10, S11) in Sec. I B). We set the radial width of the thylakoid sheet to be $\Delta R_{\rm thy} = 0.05\ R_{\rm chlor}$. The boundary conditions of Eq. (S74) are determined by the inter-compartmental transport fluxes of Ci species. Specifically, we obtain

$$\begin{cases} D^C \partial_r C_{\rm pyr} = D^C \partial_r C_{\rm thy} = \kappa^C (C_{\rm thy} - C_{\rm pyr}) \\ D^H \partial_r H_{\rm pyr} = D^H \partial_r H_{\rm thy} = \kappa^{H^0}(H^0_{\rm thy} - H^0_{\rm pyr}) + (\kappa^{H^-} + \kappa^{H^-}_{\rm thy})(H^-_{\rm thy} - H^-_{\rm pyr}) \end{cases} \qquad \text{at } r = R_{\rm pyr}, \tag{S75a}$$

$$\begin{cases} D^C \partial_r C_{\rm thy} = D^C \partial_r C_{\rm str} = \kappa^C (C_{\rm str} - C_{\rm thy}) \\ D^H \partial_r H_{\rm thy} = D^H \partial_r H_{\rm str} = \kappa^{H^0}(H^0_{\rm str} - H^0_{\rm thy}) + (\kappa^{H^-} + \kappa^{H^-}_{\rm thy})(H^-_{\rm str} - H^-_{\rm thy}) \end{cases} \qquad \text{at } r = R_{\rm pyr} + \Delta R_{\rm thy}, \tag{S75b}$$

$$\begin{cases} D^C \partial_r C_{\rm str} = \kappa^C (C_{\rm cyt} - C_{\rm str}) \\ D^H \partial_r H_{\rm str} = \kappa^{H^0}(H^0_{\rm cyt} - H^0_{\rm str}) + (\kappa^{H^-} + \kappa^{H^-}_{\rm chlor})(H^-_{\rm cyt} - H^-_{\rm str}) \end{cases} \qquad \text{at } r = R_{\rm chlor}, \tag{S75c}$$

where $\kappa^{H^-}_{\rm thy}$ and $\kappa^{H^-}_{\rm chlor}$ denote, respectively, the additional permeability to HCO_3^- due to BST channels on the thylakoid membranes and due to LCIA^{C} channels on the chloroplast envelope. We again assume that all Ci species in the cytosol are in equilibrium at pH = 7.1. As shown in Supplementary Fig. 25 (model 1), this modeled thylakoid morphology could also achieve half-saturation of Rubisco, although at a higher energetic cost. In this model, the two layers of thylakoid membranes serve as diffusion barriers to prevent CO_2 leakage out of the pyrenoid matrix, but they are less effective than the impermeable starch sheath in the Chlamydomonas PCCM model. Indeed, if we add an impermeable starch sheath with the same gaps between starch plates as the one considered in the Chlamydomonas PCCM model to further surround the shell of thylakoid sheet, i.e., multiply κ by $f_{\rm v}$ (Supplementary Table 2) in Eq. S75b, we could achieve a more efficient PCCM (Supplementary Fig. 25, model 2). This geometry is energetically even more efficient than our modeled Chlamydomonas PCCM, because the thylakoid membranes spanning the holes on the starch sheath could slow the escape of CO_2 produced in the thylakoid lumen. Note that in these models, we have assumed that low pH is always maintained in the pyrenoid-proximal thylakoids, which is presumably achieved by connecting to the surrounding thylakoids in the stroma as a source of protons.

To account this feature, we finally consider a Chlamydomonas-like configuration with a starch sheath (Fig. 2G),

but with the thylakoids inside the pyrenoid simply forming a shell of width ΔR_{thy} (Supplementary Fig. 25, model 3). The steady-state flux-balance equations are the same as Eqs. (S74) and (S75), except that

$$\text{Eq. (S74c)} \Rightarrow \begin{cases} D^C \frac{1}{f_v r^2} \frac{\partial}{\partial r}\left(f_v r^2 \frac{\partial C_{\text{tub}}}{\partial r}\right) - j^C_{\text{mem}} f_s = 0 \\ D^C \frac{1}{(1-f_v) r^2} \frac{\partial}{\partial r}\left((1-f_v) r^2 \frac{\partial C_{\text{str}}}{\partial r}\right) - j_{\text{LCIB}} + j^C_{\text{mem}} \frac{f_s f_v}{1-f_v} = 0 \\ D^H \frac{1}{f_v r^2} \frac{\partial}{\partial r}\left(f_v r^2 \frac{\partial H_{\text{tub}}}{\partial r}\right) - j^H_{\text{mem}} f_s = 0 \\ D^H \frac{1}{(1-f_v) r^2} \frac{\partial}{\partial r}\left((1-f_v) r^2 \frac{\partial H_{\text{str}}}{\partial r}\right) + j_{\text{LCIB}} + j^H_{\text{mem}} \frac{f_s f_v}{1-f_v} = 0 \end{cases} \quad \text{for } R_{\text{pyr}} + \Delta R_{\text{thy}} \leqslant r \leqslant R_{\text{chlor}} \quad \text{(S76)}$$

$$\text{Eq. (S75b)} \Rightarrow \begin{cases} \partial_r C_{\text{thy}} = \partial_r C_{\text{tub}}, C_{\text{thy}} = C_{\text{tub}}, \partial_r C_{\text{str}} = 0 \\ \partial_r H_{\text{thy}} = \partial_r H_{\text{tub}}, H_{\text{thy}} = H_{\text{tub}}, \partial_r H_{\text{str}} = 0 \end{cases} \quad \text{at } r = R_{\text{pyr}} + \Delta R_{\text{thy}}, \quad \text{(S77)}$$

describing the Ci concentrations in the stroma and in the stromal thylakoid tubules (denoted by subscript "tub"). Here, the cross-membrane fluxes j_{mem} are the same as in Eqs. (S13) and (S14). Indeed, the PCCM model using this hypothetical thylakoid morphology yields an efficacy-cost relation that closely resembles the Chlamydomonas model (Supplementary Fig. 25J). Thus, we conclude that the functioning of the PCCM is largely independent of the morphologies of intra-pyrenoid thylakoids, but that thylakoid morphologies could affect CO_2 leakage out of the pyrenoid, which determines the energetic efficiency of the PCCM.

B. Import of bicarbonate into the cell

In our model, we have assumed constant cytosolic CO_2 and HCO_3^- concentrations representing external Ci conditions. Here, we discuss how cytosolic concentrations of inorganic carbon may be maintained at steady state. Since there is no known CA in the Chlamydomonas cytosol, cytosolic CO_2 and HCO_3^- presumably need to be replenished separately, either by diffusion or transport. We expect that CO_2 can diffuse across the cell envelope, supporting the CO_2 assimilation flux by the chloroplast. A similar calculation to that in Sec. IV D can be made to estimate the conditions under which the passive CO_2 uptake strategy is feasible. Below, we focus on the transport of HCO_3^- into the Chlamydomonas cell. In particular, we estimate under limiting conditions of 10 μM HCO_3^- outside the cell (corresponding to equilibrium with air-level CO_2 at pH 6), (1) how many HCO_3^- channels are required on the cell membrane to support the flux used by the active HCO_3^- pumping strategy, and (2) the cost of synthesizing such HCO_3^- channels.

To start with, we can consider the extreme case where the rate of HCO_3^- transport into the cytosol is not limited by the turnover rate of the hypothetical HCO_3^- channels on the cell envelope, but is rather limited by diffusion through the cavity of these channels. In this case, the diffusive flux through a single channel can be estimated by $J_{\text{hole}} = 2D^H r_{\text{hole}} H^-_{\text{env}}/(1 + \frac{2h}{\pi r_{\text{hole}}})$ [61], where $D^H \approx 10^3$ μm^2/s denotes the diffusion constant of HCO_3^-, $r_{\text{hole}} \approx 1$ nm denotes the radius of the cavity, $h \approx 5$ nm denotes the length of the channel and $H^-_{\text{env}} = 10$ μM denotes the bulk concentration of HCO_3^- in the environment. Plugging in these numbers yields a flux of $J_{\text{hole}} = 0.5 \times 10^{-20}$ mol/s. Since this estimation gives the upper limit, we choose to use a value of $J_{\text{hole}} = 0.5 \times 10^{-21}$ mol/s, i.e., an order of magnitude smaller than the upper bound, as a more realistic estimation of the single-channel flux. Our simulation results show that an active HCO_3^- pumping flux of 2.4×10^{-16}mol/s across the chloroplast envelope can drive > 70% of the maximum CO_2 fixation flux (Supplementary Fig. 12). To support this flux, a total of 4.8×10^5 channels are needed. Finally, one can estimate the fraction of the cell membrane surface area occupied by this many channels. Assuming that the outer radius of the channel is 3.5 nm (a typical value) and that the radius of a Chlamydomonas cell is 5 μm, one can obtain the surface area fraction taken by the channels to be $4.8 \times 10^5 \times (3.5\ \text{nm})^2/(5\ \mu\text{m})^2/4 \approx 6\%$.

To estimate the energetic cost of synthesizing the above number of channels, we consider the synthesis and polymerization of 1000 amino acids per channel, similar to the size of the putative HCO_3^- transporters on the Chlamydomonas cell membrane. Assuming that the cost of protein synthesis is 30 ATP/aa, we obtain the cost to synthesize one HCO_3^- transporter to be 30,000 ATP. The doubling time of Chlamydomonas cells across a wide range of conditions has been measured to be 10 hours [63]. Over this period of time, a Chlamydomonas cell presumably needs to synthesize 4.8×10^5 transporters as discussed above, yielding an energy consumption rate of (30,000 ATP/transporter) × (4.8×10^5 transporters) / (10 hours) = 4×10^5 ATP/s. To compare this cost with the cost of operating the PCCM, we employ the simulation results that the saturating CO_2 fixation flux is 10^{-16} mol/s and the cost is roughly 1 ATP per CO_2 fixed (see main Fig. 3). Thus, an effective PCCM will cost $\sim 6.0 \times 10^7$ ATP/s, which is roughly two orders

of magnitude larger than the cost of synthesizing the HCO_3^- transporters.

C. Ci diffusion in the pyrenoid

In our calculation in Section I H, we assume a value of $D_0 = 1.88 \times 10^3$ µm^2/s for the diffusion coefficient of CO_2 inside the pyrenoid matrix. The crowding of proteins in Rubisco condensates could in principle slow the diffusion of CO_2 in the pyrenoid matrix. Here, we calculate whether this crowding would have a significant effect on CO_2 leakage out of the pyrenoid. The concentration of Rubisco enzymes in the pyrenoid matrix was previously measured to be 377,856 Rubiscos/µm^3 [2]. The volume of each Rubisco protein V_{Rbc} can be estimated from its crystal structure (see for example, PDB ID:7JN4), which yields $\Omega_{\text{Rbc}} \approx 10^3$ nm^3. Taken together, we obtain that the volume fraction of the pyrenoid matrix occupied by Rubisco is $\phi_{\text{Rbc}} \approx 0.38$, and that the volume fraction of the void presumably filled with water is $\phi_{\text{void}} \approx 0.62$. Using the formula for the effective diffusion coefficient in porous media [64], we estimate the effective diffusion coefficient of CO_2 in the pyrenoid matrix to be $D_{\text{eff}} = \frac{\phi_{\text{void}} D_0}{1-\frac{\ln \phi_{\text{void}}}{2}} \approx 0.50 D_0 = 0.94 \times 10^3$ µm^2/s, where $D_0 = 1.88 \times 10^3$ µm^2/s is the diffusion coefficient of CO_2 in water. Taking into account this slower diffusion in the pyrenoid matrix, we obtain that the CO_2 leakage percentage from the pyrenoid matrix is 75% in the absence of diffusion barriers. This result differs only slightly from the original result of 86% using D_0 for the diffusion coefficient of CO_2 in the pyrenoid matrix. Thus, the *in vivo* volume fraction of the matrix occupied by Rubisco is not high enough to dramatically slow CO_2 diffusion, so we have neglected the minor effect of slower diffusion in the pyrenoid matrix for simplicity.

D. Proton transport

In our model, we have focused on the reaction and transport of inorganic carbon molecules, namely, CO_2, HCO_3^- and H_2CO_3, and we have not explicitly considered protons. *In vivo*, protons are produced and consumed in reactions catalyzed by carbonic anhydrases. Additionally, Rubisco catalyzes the addition of CO_2 and H_2O to ribulose-1,5-bisphosphate (RuBP) to yield two molecules of 3-phosphoglycerate (3PGA) and two protons for every CO_2 fixed. In this section, we will discuss how protons consumed/produced in different compartments can be replenished/depleted to maintain pH. Specifically, we first examine whether free diffusion of protons is sufficient for pH maintenance.

pH maintenance of the pyrenoid matrix. Consider a scenario where the PCCM is functional and Rubisco CO_2 fixation flux is close to its maximum $J^C_{\text{max}} \approx 4\pi R^3_{\text{pyr}} V^C_{\text{max,Rbc}}/3$. Introducing $R_{\text{pyr}} \approx 1$ µm and $V^C_{\text{max,Rbc}} = 29$ mM/s, we obtain the total proton production flux to be $2J^C_{\text{max}} \approx 2.4 \times 10^{-16}$ mol/s. At steady state, the overall proton efflux must be equal to $2J^C_{\text{max}}$ in order to ensure flux balance in the pyrenoid matrix.

One possibility is that free protons in the pyrenoid matrix could diffuse to the intra-pyrenoid thylakoid membrane where they can be pumped into the intra-pyrenoid thylakoid lumen. To estimate the flux across the thylakoid membranes, we consider the diffusion-absorption process around a cylindrical tubule of radius a_{tub}, similar to the one discussed in Sec. IV D. Free diffusion of protons outside the tubule is described by $\rho^{-1}\partial_\rho(\rho\partial_\rho P) = 0$ where ρ denotes the perpendicular distance from the central axis of the tubule and P denotes the proton concentration. The boundary conditions are given by (1) $D^P(\partial_\rho P)|_{\rho=a_{\text{tub}}} = \kappa^P P|_{\rho=a_{\text{tub}}}$, in which D^P denotes the diffusion coefficient of protons and κ^P denotes the rate of potential proton transport across the thylakoid membrane, and (2) $P|_{\rho=L} = P_\infty$, where L denotes the typical spacing between thylakoid tubules and P_∞ denotes the bulk proton concentration in the pyrenoid matrix. Solving the diffusion-absorption problem yields $P(\rho) = P_\infty\left[1 + \frac{\ln(\rho/L)}{D^P/(\kappa^P a_{\text{tub}}) + \ln(L/a_{\text{tub}})}\right]$. Thus, the total transport flux by diffusion is given by $\Delta S_{\text{thy}} D^P(\partial_\rho P)|_{\rho=a_{\text{tub}}} = \Delta S_{\text{thy}} \kappa^P P_\infty \frac{D^P/a_{\text{tub}}}{D^P/a_{\text{tub}} + \kappa^P \ln(L/a_{\text{tub}})}$ where ΔS_{thy} denotes the total surface area of the thylakoid membranes inside the pyrenoid. Note that when diffusion is fast, i.e., $D^P/a_{\text{tub}} \gg \kappa^P$, the absorption flux is given by $J^P = \Delta S_{\text{thy}} \kappa^P P_\infty$, and when absorption is diffusion limited, i.e., $D^P/a_{\text{tub}} \ll \kappa^P$, the maximum absorption flux is given by $J^P_{\text{max}} = \Delta S_{\text{thy}} P_\infty \frac{D^P/a_{\text{tub}}}{\ln(L/a_{\text{tub}})}$. Introducing $\Delta S_{\text{thy}} = 8.5$ µm^2 (Supplementary Tables 2 and 4), $P_\infty = 10^{-8}$ M [2], $D^P = 10^4$ µm^2/s [65], $a_{\text{tub}} = 0.05$ µm (Supplementary Table 2), and $L = 10\ a_{\text{tub}}$ into the expression, we obtain $J^P_{\text{max}} = 0.074 \times 10^{-16}$ mol/s, which is one order of magnitude smaller than the proton production flux. In other words, the pH of the pyrenoid matrix would have to be lower than 6.5, i.e., $P_\infty > 3.24 \times 10^{-7}$ M, in order for the protons produced during CO_2 fixation to enter the thylakoid tubules as free protons.

Another possibility is that free protons could diffuse into the stroma, where they are then pumped back into the

thylakoid lumen by Photosystems I and II [52, 66, 67]. Assuming that the starch sheath does not serve as a barrier to proton diffusion, we can estimate the maximum efflux from the Burg-Purcell limit [68] (see also Sec. IV D), which yields $J^P_{\rm max} = 4\pi R_{\rm pyr} D^P \Delta P$. Here, ΔP denotes the concentration difference of protons between the pyrenoid matrix and the stroma. Thus, ΔP must be greater than $\frac{J^P_{\rm max}}{4\pi R_{\rm pyr} D^P} = \frac{2.4\times10^{-16}\ \rm mol/s}{4\pi\times(1\ \mu m)\times10^4\ \mu m^2/s} = 1.91 \times 10^{-6}$ M, i.e., the pH of the pyrenoid matrix must be lower than 5.7, in order for protons to be removed sufficiently fast from the pyrenoid matrix by free diffusion to the stroma.

Thus, both possible free-proton diffusion based mechanisms for pH maintenance in the pyrenoid matrix require an acidic matrix. This clearly disagrees with previous measurements showing that the pH values of both the pyrenoid matrix and stroma are about 8 [2].

pH maintenance of the lumen of thylakoid tubules. In the intra-pyrenoid thylakoid lumen where CAH3 are present, protons are consumed in the HCO_3^--to-CO_2 conversion catalyzed by CAH3. When the PCCM is functional and Rubisco is almost fully saturated by CO_2, our simulation results show that roughly 1 out of 3 CO_2 molecules produced by CAH3 is fixed by Rubisco (Supplementary Fig. 7F). Thus, we estimate the total proton consumption flux in the intra-pyrenoid thylakoid lumen, which is equal to the flux of CAH3-catalyzed reaction, to be $\gtrsim 3J^C_{\rm max} = 3.6 \times 10^{-16}$ mol/s. We wonder whether these protons can be replenished by diffusion of free protons from the extra-pyrenoid to the intra-pyrenoid thylakoid lumen. Similar to our estimation of fluxes in the well-mixed compartment model (Sec. IV), we estimate the maximum influx of free-proton diffusion as $J^P_{\rm max} = \frac{N_{\rm tub}\pi a^2_{\rm tub}}{L^P} D^P \Delta P$, where $N_{\rm tub}$ and $a_{\rm tub}$ denote, respectively, the number and radius of thylakoid tubules, L^P denotes the typical diffusion length scale of protons along the thylakoid tubules, and ΔP denotes the concentration difference of protons between the extra-pyrenoid and intra-pyrenoid thylakoid lumen. To obtain an upper-bound estimate of $J^P_{\rm max}$, we consider the case where the pH of the acidic thylakoid lumen is 5, and we estimate $\Delta P \approx 10^{-2}$ mM. Introducing $N_{\rm tub} = 40$, $a_{\rm tub} = 0.05$ μm, $L^P \approx 0.3$ μm, $D^P = 10^4$ μm^2/s, and $\Delta P \approx 10^{-2}$ mM into the expression for $J^P_{\rm max}$, we obtain an upper-bound estimate $J^P_{\rm max} \approx 10^{-16}$ mol/s, which is smaller than the lower-bound estimate for the proton consumption flux. In other words, the pH in the thylakoid lumen would have to be lower than 4.5, i.e., $\Delta P > 3 \times 10^{-2}$ mM, in order for the protons consumed by CAH3 to be replenished as free protons. This is inconsistent with previous measurements suggesting that the pH of the thylakoid lumen is about 5.7 [69].

Possible mechanisms of proton transport. The above calculations suggest that free-proton concentrations both in the thylakoid lumen and in the matrix are too low to mediate the expected effective proton fluxes by free-proton diffusion (Supplementary Fig. 27), so efficient transport of protons would require additional mechanisms. One possible mechanism involves proton carriers; they could exist at millimolar concentrations in chloroplast compartments. Specifically, proton carriers would be helpful both for (1) recycling the protons released during the fixation of CO_2 back into the intra-pyrenoid thylakoid lumen, and (2) carrying protons from the extra-pyrenoid thylakoid lumen to the intra-pyrenoid thylakoid lumen. A recent study has also highlighted this point and suggests that protonation of RuBP and PGA could play an important role in buffering pH [70]. We speculate that proton carriers such as RuBP and 3PGA are crucial for pH maintenance. As an example, given its pKa of 6.7, RuBP will predominantly exist in the form of RuBP^{4-} in the stroma outside the pyrenoid where the pH is 8. If there are RuBP transporters or channels specific to RuBP^{4-} on the thylakoid membranes, this form could enter the thylakoid lumen (pH 6) and pick up a proton to become RuBP^{3-}, which would then be trapped in the thylakoids. From there, RuBP^{3-} could diffuse toward the pyrenoid carrying its proton. If this proton were then consumed in converting HCO_3^- to CO_2 in the tubules, RuBP^{4-}, the substrate for Rubisco's carboxylation reaction, would be left to diffuse into the pyrenoid matrix. Besides proton carriers, the overall pH maintenance also involves other processes (such as proton pumping driven by light reactions) and thus must be a complex synergy of various mechanisms. Understanding the mechanisms underlying proton transport will be an important topic for future experimental studies, and will help to validate the hypotheses presented here and elsewhere.

E. Effect of LCIB-Rubisco complexes

Modeling the transfer of PCCM machinery into a plant cell requires an approximation of the starting chloroplast configuration in plants (Fig. 8). In our model, we have assumed that plant chloroplasts lack any CO_2-concentrating activity. Here, we offer a justification for this assumption. Previous experiments have suggested that carbonic anhydrases (CAs) and Rubisco could form protein complexes in certain plant chloroplasts [71, 72]. Based on these observations, it has been suggested that protons produced during CO_2 fixation could locally decrease the pH, favoring the production of CO_2. A recent work [70] suggests that in a modeled compartment with a diffusion barrier, Rubisco-

derived protons could indeed drive the conversion of HCO_3^- to CO_2 via co-condensed CA, thus increasing local CO_2 concentration and the rate of Rubisco CO_2 fixation. We wondered whether a similar mechanism could work for protein complexes of plant Rubisco with a stromal CA, hence rendering noticeable CO_2-concentrating activity to the plant chloroplasts.

Consider a condensate of Rubisco and LCIB whose radius is denoted by R_{cond}. We explicitly consider the reactions involving CO_2, HCO_3^-, and H^+ inside the condensate, as well as their diffusion in and out of the condensate (Supplementary Fig. 29A). Denote by P, C, and H^- the concentrations of protons, CO_2, and HCO_3^-, respectively. At steady state, the flux balance of HCO_3^- in the condensate requires that the total flux of HCO_3^--to-CO_2 conversion by LCIB should be equal to the influx of HCO_3^-, i.e.,

$$\frac{4}{3}\pi R_{\mathrm{cond}}^3 k_{\mathrm{LCIB}}\Big(\frac{P}{P_{\mathrm{eq}}}H^- - C\Big) = 4\pi R_{\mathrm{cond}} D^H (H_\infty^- - H^-), \tag{S78}$$

where k_{LCIB} denotes the first-order rate constant of LCIB, $P_{\mathrm{eq}} = 10^{-\mathrm{pK_{eff}}} = 10^{-6.1}$ M denotes the proton concentration at which CO_2 and HCO_3^- have equal equilibrium concentrations, and H_∞^- denotes the concentration of HCO_3^- in the bulk stroma away from the condensate. Here, we have used a linear approximation for CA kinetics, and we have again used the Burg-Purcell calculation for the HCO_3^- influx. At steady state, CO_2 converted by LCIB must either be fixed by Rubisco or diffuse out of the condensate, i.e.,

$$\frac{4}{3}\pi R_{\mathrm{cond}}^3 k_{\mathrm{LCIB}}\Big(\frac{P}{P_{\mathrm{eq}}}H^- - C\Big) = \frac{4}{3}\pi R_{\mathrm{cond}}^3 V_{\mathrm{max,Rbc}}^C \frac{C}{C + K_{\mathrm{m}}^{\mathrm{eff}}} + 4\pi R_{\mathrm{cond}} D^C (C - C_\infty), \tag{S79}$$

where C_∞ denotes the concentration of CO_2 in the bulk stroma. Finally, the protons involved in the HCO_3^--to-CO_2 conversion must either come from Rubisco CO_2 fixation or from the bulk stroma, which yields

$$\frac{4}{3}\pi R_{\mathrm{cond}}^3 k_{\mathrm{LCIB}}\Big(\frac{P}{P_{\mathrm{eq}}}H^- - C\Big) = 2\times\Big(\frac{4}{3}\pi R_{\mathrm{cond}}^3 V_{\mathrm{max,Rbc}}^C \frac{C}{C + K_{\mathrm{m}}^{\mathrm{eff}}}\Big) + 4\pi R_{\mathrm{cond}} D_P (P_\infty - P). \tag{S80}$$

Here, the factor 2 accounts for the two protons produced for each CO_2 fixed.

Choice of parameters. Consider a stroma of pH 8 under air-level CO_2 (10 μM cytosolic), i.e., $P_\infty = 10^{-8}$ M $= 10^{-5}$ mM, and $C_\infty = 10$ μM $= 0.01$ mM. We further assume that HCO_3^- is equilibrated with CO_2, i.e., $H_\infty^- = 10^{2.9}$ μM ≈ 0.794 mM (however, this assumption is not crucial for the main conclusion we will draw below). We assume that the Rubisco density is the same as that in the phase separated droplet in Chlamydomonas, and thus $V_{\mathrm{max,Rbc}}^C \approx 19$ mM/s. $K_{\mathrm{m}}^{\mathrm{eff}}$ of typical plant Rubisco is 0.021 mM (Supplementary Table 2). We choose $k_{\mathrm{LCIB}} = 10^6$ s^{-1} since the typical turnover number for CA is 10^6 s^{-1} and in a condensate the concentration of protein could be in the mM range, similar to the K_{m} of typical CA. The values of the diffusion coefficients are the same as previous sections (see Sec. I A, Sec. VI D and Supplementary Table 2).

Solving Eqs. (S78 – S80) at varying R_{cond}, we find that for small R_{cond}, the reaction fluxes of enzymes in the condensate are negligible since they scale with R_{cond}^3 while the absorption/leakage fluxes scale with R_{cond}, and thus the concentration of CO_2 in the condensate is roughly the same as the environment (Supplementary Fig. 29B). The mechanism discussed above doubles the CO_2 concentration in the condensate roughly when the two terms on the right-hand side of Eq. (S79) are comparable, i.e., when $R_{\mathrm{cond}} \gtrsim (3D^C C_\infty / V_{\mathrm{max,Rbc}}^C)^{1/2} \approx 2$ μm, which is much larger than the size of protein complexes we consider. In the limit of large R_{cond}, the absorption of carbon molecules become diffusion-limited, which leads to a low CO_2 concentration in the condensate again (Supplementary Fig. 29C).

SUPPLEMENTARY FIGURES AND TABLES

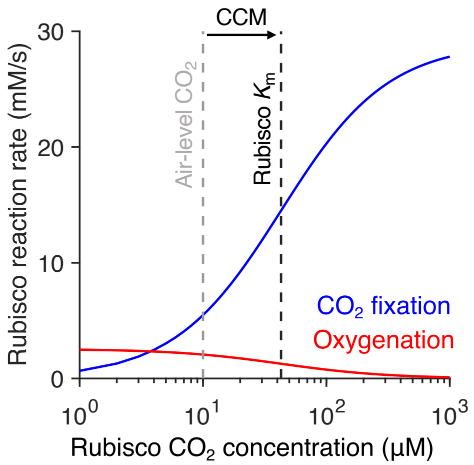

Supplementary Fig. 1. CO_2 fixation by Rubisco is inhibited by oxygen at air-level CO_2. Rates of CO_2 fixation (blue, Eq. S11) and oxygenation (red, Eq. S27) by Rubisco at varying CO_2 concentrations. Gray dashed line denotes air-level CO_2 (10 μM cytosolic). Black dashed line denotes the effective K_m of Rubisco (43 μM, see Sec. I B). Kinetic parameters of Chlamydomonas Rubisco are listed in Supplementary Table 2.

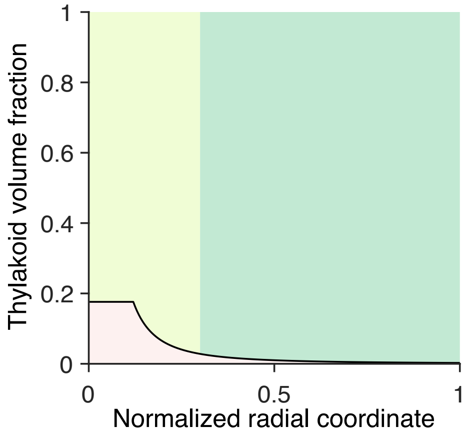

Supplementary Fig. 2. Volume fraction of the modeled chloroplast compartments. Volume fraction of the chloroplast taken up by the thylakoid tubules (pink), pyrenoid matrix (light green), and stroma (green), versus the normalized radial coordinate. Note that our model does not consider the volume of thylakoid stacks traversing the stroma.

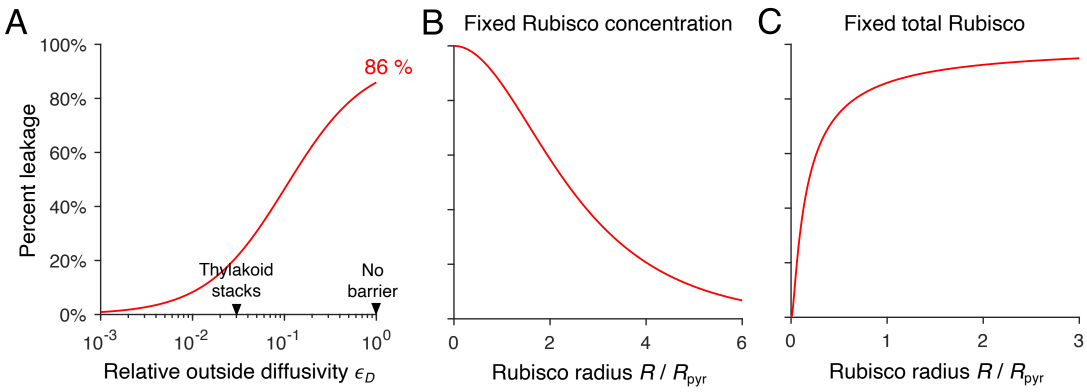

Supplementary Fig. 3. Low fixation efficiency at air-level CO_2 (10 μM cytosolic) in the absence of a diffusion barrier is due to severe CO_2 leakage from the pyrenoid matrix. CO_2 percent leakage computed from a simplified reaction-diffusion model (see Sec. I H for details) for (*A*) varying relative diffusivity ϵ_D outside the sphere of Rubisco, and at varying hypothetical Rubisco radii assuming (*B*) fixed Rubisco concentration or (*C*) fixed number of Rubisco molecules. In *A*, Rubisco radius $R = R_{\text{pyr}}$, and arrowheads mark the ϵ_D values corresponding to the case of diffusion slowed by thylakoid stacks and the case of no diffusion barrier. In *B* and *C*, relative diffusivity outside the Rubisco region compared to inside the Rubisco region is $\epsilon_D = 1$.

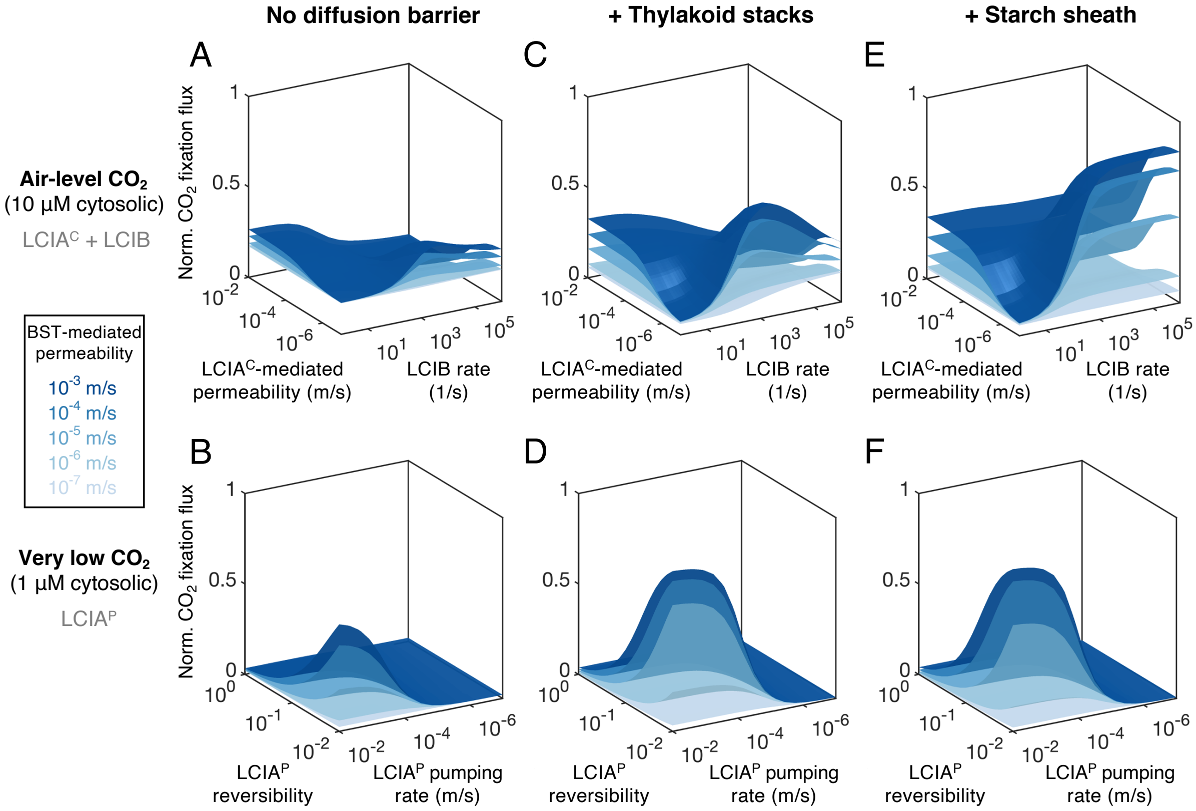

Supplementary Fig. 4. CO_2 fixation flux increases with BST-mediated thylakoid membrane permeability to HCO_3^-. This is verified in (*A*, *B*) a model without a diffusion barrier, (*C*, *D*) a model with thylakoid stacks slowing inorganic carbon diffusion in the stroma, and (*E*, *F*) a model with an impermeable starch sheath. (*A*, *C*, *E*) A modeled chloroplast employing LCIB and $LCIA^C$ HCO_3^- channels is considered under air-level CO_2 (10 µM cytosolic). Normalized CO_2 fixation fluxes at varying $LCIA^C$-mediated permeabilities to HCO_3^- and varying LCIB rates are shown for the designated BST-mediated permeability. (*B*, *D*, *F*) A modeled chloroplast employing $LCIA^P$ HCO_3^- pumps and no LCIB is considered under very low CO_2 (1 µM cytosolic). Normalized CO_2 fixation fluxes at varying $LCIA^P$ transport rates and reversibilities are shown for the designated BST-mediated permeability.

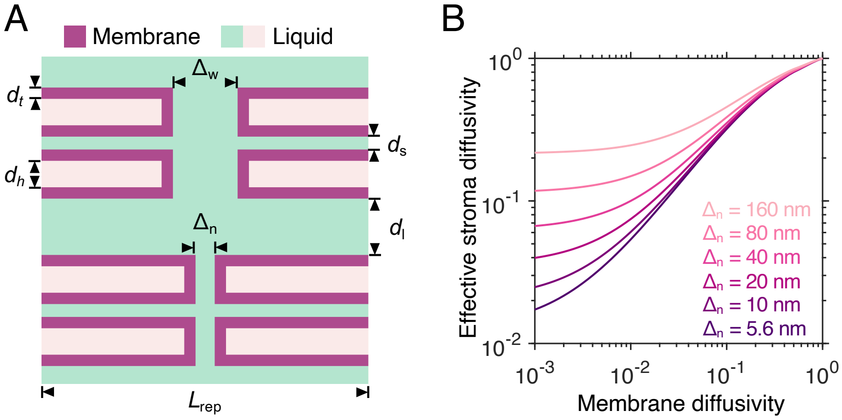

Supplementary Fig. 5. Model for the effect of thylakoid stacks on diffusion in the stroma. (*A*) Schematic geometry of modeled thylakoid stacks. Key geometric parameters are described in Sec. I G. Color code is the same as in Fig. 1A. (*B*) Effective stroma diffusivity plotted against membrane diffusivity for the designated gap sizes Δ_n of the thylakoid stacks. Diffusivities are normalized by the diffusion coefficient in liquid (see Sec. I G for details). $\Delta_\mathrm{w} - \Delta_\mathrm{n}$ is fixed when varying Δ_n in the simulations. We infer from previous experiments that $\Delta_\mathrm{n} = 5.6$ nm [1], and relative membrane diffusivity is roughly 10^{-3} [29]. Values of other geometric parameters are provided in Sec. I G **Choice of parameters**.

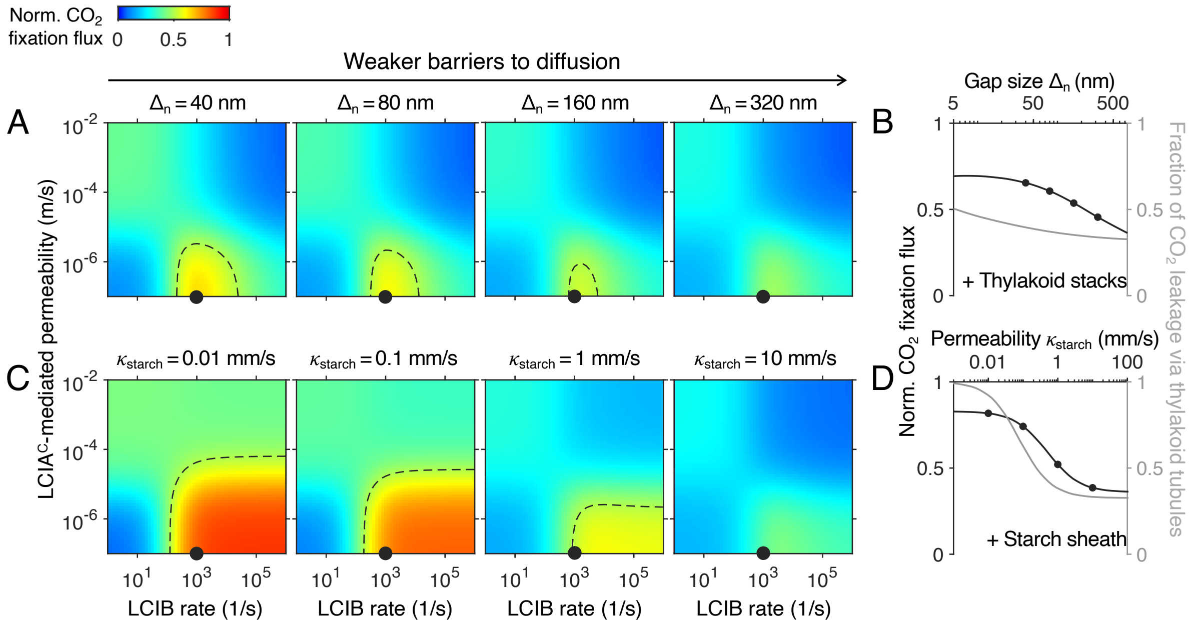

Supplementary Fig. 6. CO_2-concentrating performance is compromised by weaker barriers to the diffusion of inorganic carbon. A model with thylakoid stacks slowing inorganic carbon diffusion in the stroma (*A*, *B*) is compared to a model with a starch sheath (*C*, *D*) under air-level CO_2 (10 μM cytosolic). (*A*, *C*) Heatmaps of normalized CO_2 fixation flux (see Sec. II A) at varying $LCIA^C$-mediated chloroplast membrane permeabilities to HCO_3^- and varying LCIB activities shown for the designated parameters of the two diffusion barriers. Plots correspond to Figs. 2*E* and *H*. (*B*, *D*) Normalized CO_2 fixation flux (black) and fraction of CO_2 leakage from the pyrenoid that occurs via thylakoid tubules (and not directly from the pyrenoid matrix into the stroma) (gray) at varying strengths of the diffusion barriers. $LCIA^C$-mediated permeability and LCIB activity are indicated by black dots in *A* and *C*.

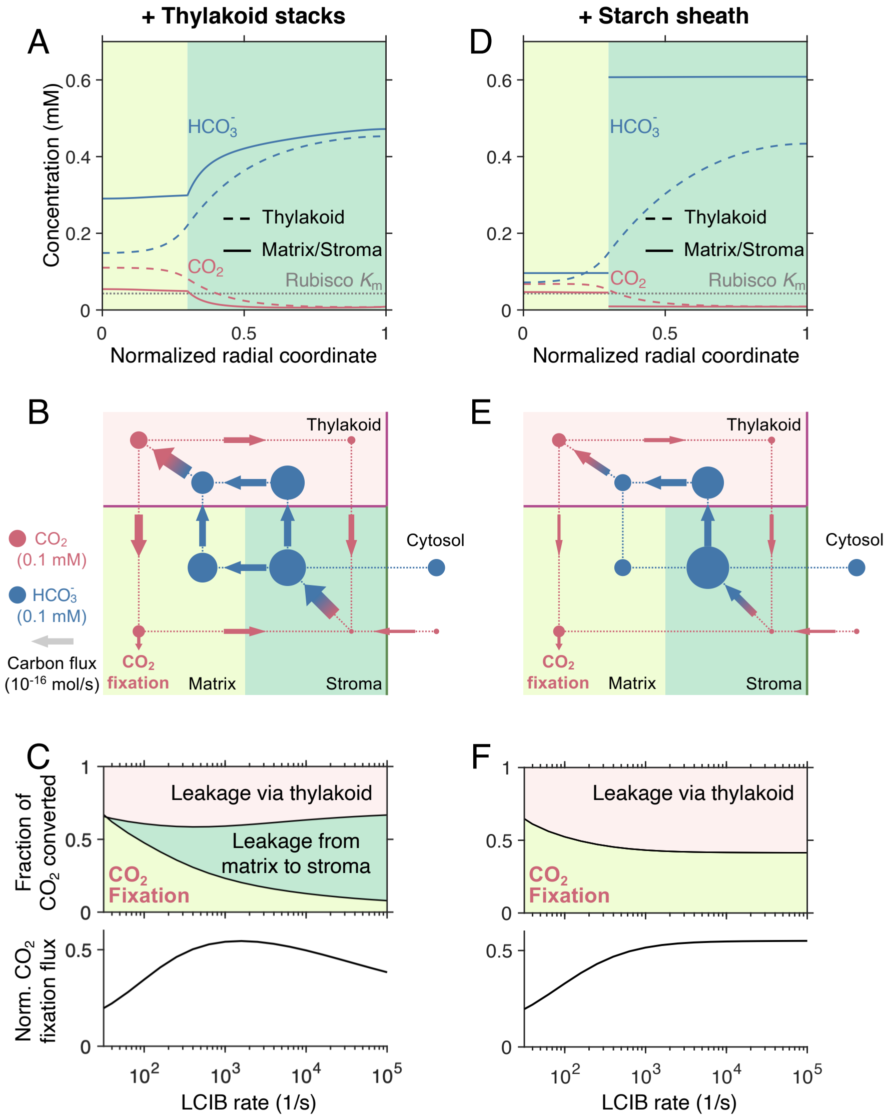

Supplementary Fig. 7. Comparison between two types of diffusion barriers under air-level CO_2 (10 μM cytosolic). A model with thylakoid stacks slowing inorganic carbon diffusion in the stroma (A–C) is compared to a model with an impermeable starch sheath (D–F). (A, D) Concentration profiles of CO_2 and HCO_3^- in the thylakoid (dashed curves) and in the matrix/stroma (solid curves). Dotted gray line indicates the effective Rubisco K_m for CO_2 (see Sec. I B). Plots correspond to Fig. 1C. (B, E) Net fluxes of inorganic carbon between the indicated CCM compartments. Plots correspond to Fig. 1D. (C, F) Decomposition of the total CO_2 flux converted by CAH3 (top) and normalized CO_2 fixation flux (bottom, see Sec. II A for definition) at varying rates of LCIB activity. Both models have minimal HCO_3^- transport across the chloroplast envelope. BST-mediated thylakoid membrane permeability to HCO_3^- is set to $\kappa_{\text{thy}}^{H^-} = 10^{-4}$ m/s in A–C and $\kappa_{\text{thy}}^{H^-} = 10^{-4.8}$ m/s in D–F, which are chosen such that the maximum normalized CO_2 fixation flux is roughly 0.5. LCIB rates are $V_{\text{max,LCIB}}^C / K_m^C = 10^3$ s^{-1} in A–B and D–E.

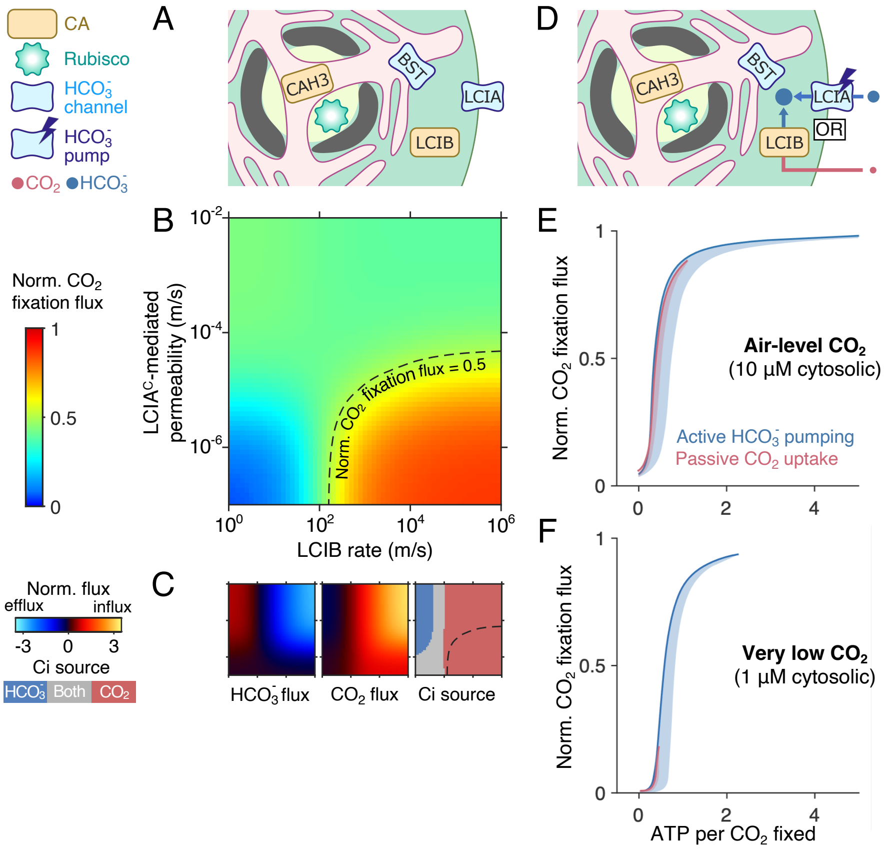

Supplementary Fig. 8. A model including both thylakoid stacks and an impermeable starch sheath behaves similarly to a model with only the latter. (*A*) Schematic of a modeled chloroplast with both thylakoid stacks and impermeable starch sheath, employing LCIB and LCIA^{C} HCO_3^- channel. (*B*) Heatmap of normalized CO_2 fixation flux (see Sec. II A) at varying LCIA^{C}-mediated chloroplast membrane permeabilities to HCO_3^- channel and varying LCIB rates. (*C*) Heatmaps of (*Left subpanels*) the total HCO_3^- flux, (*Middle subpanels*) the total CO_2 flux, and (*Right subpanels*) the type(s) of inorganic carbon (Ci), taken up by the chloroplast. Fluxes are normalized by the maximum CO_2 fixation flux for saturated Rubisco. Negative values of flux indicate efflux. The x and y axes are the same as in *B*. Panels *A*-*C* correspond to Fig. 2. In *B* and *C*, parameters are the same as in Fig. 2. (*D*) Schematic of a modeled chloroplast with both thylakoid stacks and impermeable starch sheath, employing LCIA^{P} HCO_3^- pumping across the chloroplast envelope and no LCIB activity (blue), or employing LCIB for passive CO_2 uptake (red). (*E, F*) PCCM performance under (*E*) air-level CO_2 (10 µM cytosolic) and (*F*) very low CO_2 (1 µM cytosolic), measured by normalized CO_2 fixation flux versus ATP spent per CO_2 fixed (see Sec. II B), for the two PCCM strategies in *D*. Solid curves indicate the minimum energy input necessary to achieve a certain normalized CO_2 fixation flux. Shaded regions represent the range of possible performances found by varying HCO_3^- transport rates and/or LCIB rates. Panels *D*-*F* correspond to Fig. 3.

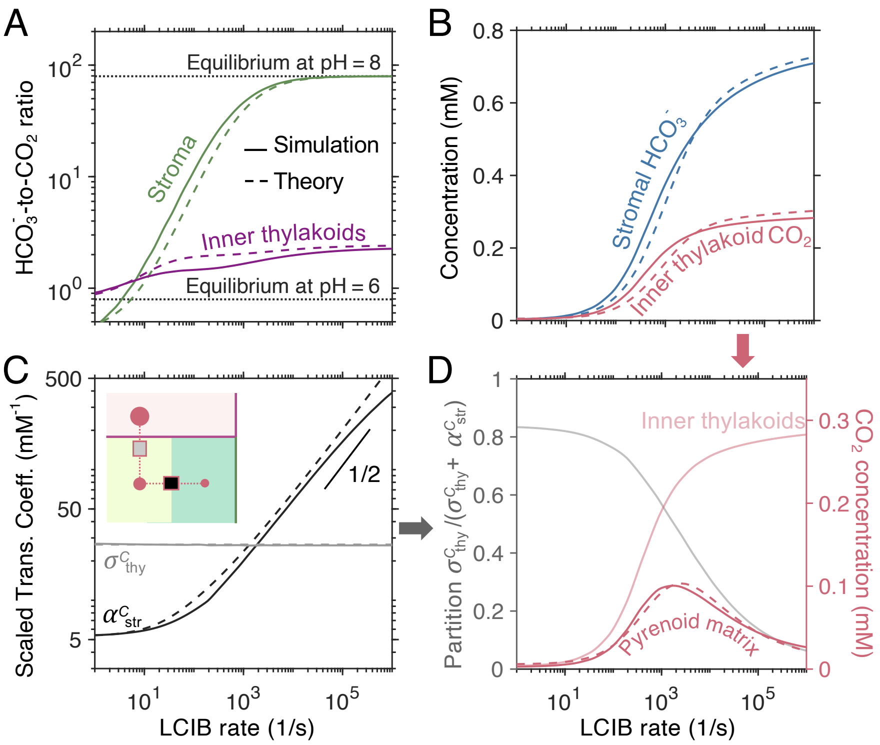

Supplementary Fig. 9. In a modeled chloroplast employing thylakoid stacks and no starch sheath, LCIB activity increases intra-chloroplast HCO_3^- concentration by converting external CO_2 but also effectively "steals" CO_2 from the pyrenoid matrix, leading to a nonmonotonic impact of LCIB rate on CO_2 concentration inside the pyrenoid matrix. (*A*) The concentration ratio of HCO_3^- to CO_2 in the stroma (green) and in the thylakoid tubules inside the pyrenoid (purple; denoted by inner thylakoids) at varying LCIB rates. Black dotted lines indicate the equilibrium HCO_3^--to-CO_2 ratio for the designated pH values. (*B*) Concentrations of HCO_3^- in the stroma (blue) and CO_2 in the inner thylakoids increase with LCIB rates. (*C*) The linear transport coefficients of CO_2, defined in Sec. IV C, between the thylakoid tubules and pyrenoid matrix (gray) and between the matrix and stroma (black) at varying LCIB rates. *Inset:* schematic of the transport coefficients. Color code is the same as Fig. 1D. The short black line indicates a slope of 1/2 on a log-log scale. (*D*) Decreasing partition factor $\sigma^C_{\rm thy}/(\alpha^C_{\rm str}+\sigma^C_{\rm thy})$ defined in Eq. S69 (light gray) and increasing concentration of CO_2 in the inner thylakoids (light red) with increasing LCIB rates together lead to the nonmonotonic dependence of pyrenoid matrix CO_2 level on the rate of LCIB. Solid and dashed curves denote results from the full simulation and the simplified model Eq. S68, respectively.

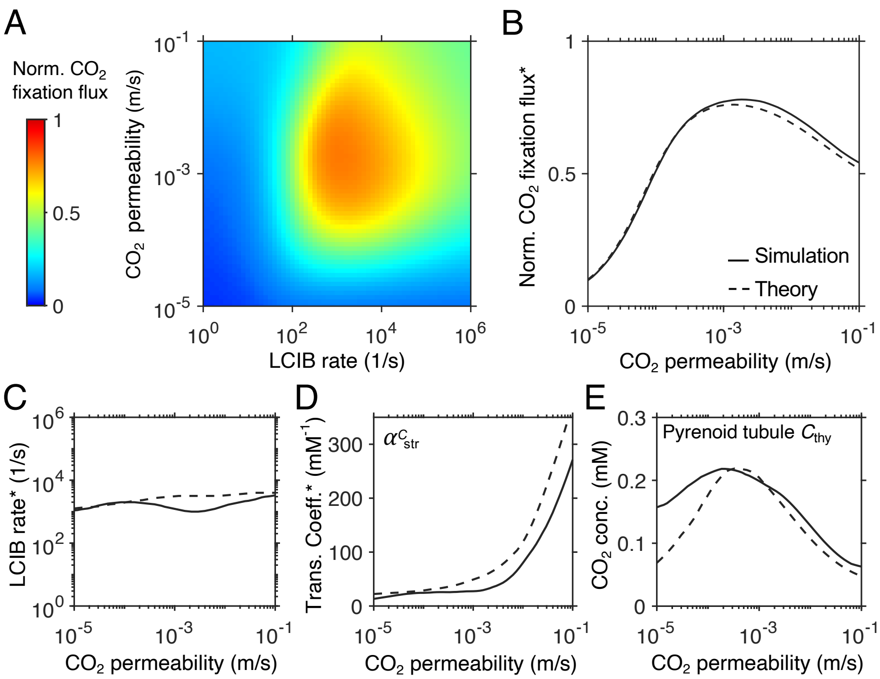

Supplementary Fig. 10. A trade-off between CO_2 transport and CO_2 leakage leads to the existence of an intermediate membrane permeability to CO_2 that optimizes CO_2-concentrating performance. (*A*) Normalized CO_2 fixation flux at varying LCIB rates and membrane permeabilities to CO_2 (κ^C) in a modeled chloroplast with thylakoid stacks slowing the diffusion of inorganic carbon in the stroma and no starch sheath. (*B*, *C*) The maximum normalized CO_2 fixation flux (shown in *B*) and the correponding LCIB rate (shown in *C*), denoted by superscript *, at varying membrane permeabilities to CO_2. (*D*, *E*) The transport coefficient $\alpha^C_{\rm str}$ between the pyrenoid matrix and stroma (shown in *D*; see Sec. V B) and the CO_2 concentration $C_{\rm thy}$ in the pyrenoid tubules (shown in *E*) at varying membrane permeabilities to CO_2 when normalized CO_2 fixation flux is maximized with respect to varying LCIB rates. In *B*–*E*, solid and dashed curves denote results from the full simulation and the simplified model Eq. S68, respectively.

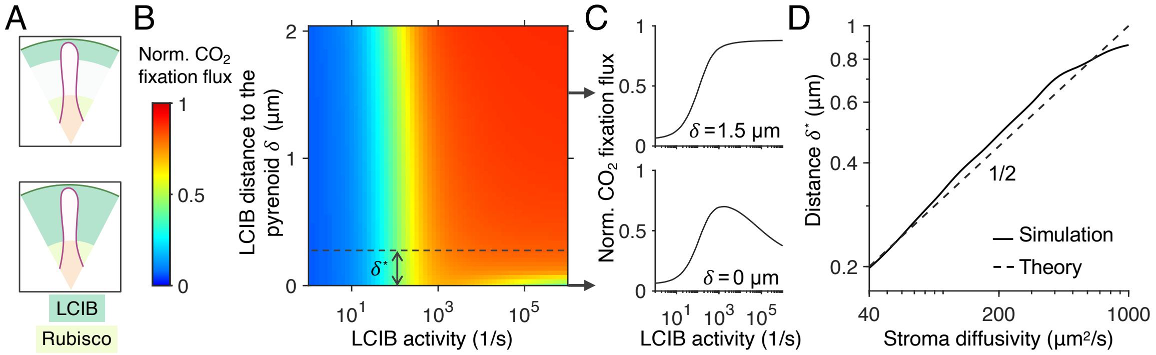

Supplementary Fig. 11. Localizing LCIB close to the chloroplast envelope prevents excessive CO_2 efflux drawn by LCIB from the pyrenoid matrix in a modeled chloroplast with thylakoid stacks slowing diffusion in the stroma and no starch sheath. (*A*) Schematics of hypothetical LCIB localization patterns. (*B*) Normalized CO_2 fixation flux at varying LCIB activities and LCIB localization patterns. LCIB activity is characterized by the first-order rate constant when LCIB is uniformly distributed in the stroma. LCIB localization is characterized by its distance δ to the pyrenoid. For a given LCIB activity, the total number of LCIB is held constant when varying the localization pattern. (*C*) Normalized CO_2 fixation flux at varying LCIB activities for the designated values of δ, indicated by the arrows in *B*. (*D*) The critical distance δ^* between the pyrenoid and LCIB, defined as the distance above which the normalized CO_2 fixation flux increases monotonically with LCIB activity (indicated in *B*), at varying CO_2 stroma diffusivities is plotted for the simulation result (solid) and the theoretical prediction (dashed; see Sec. V C **Localization of LCIB**). 1/2 indicates the slope of the theory line on a log-log scale. For all panels, simulation parameters are the same as Figs. 1C and D.

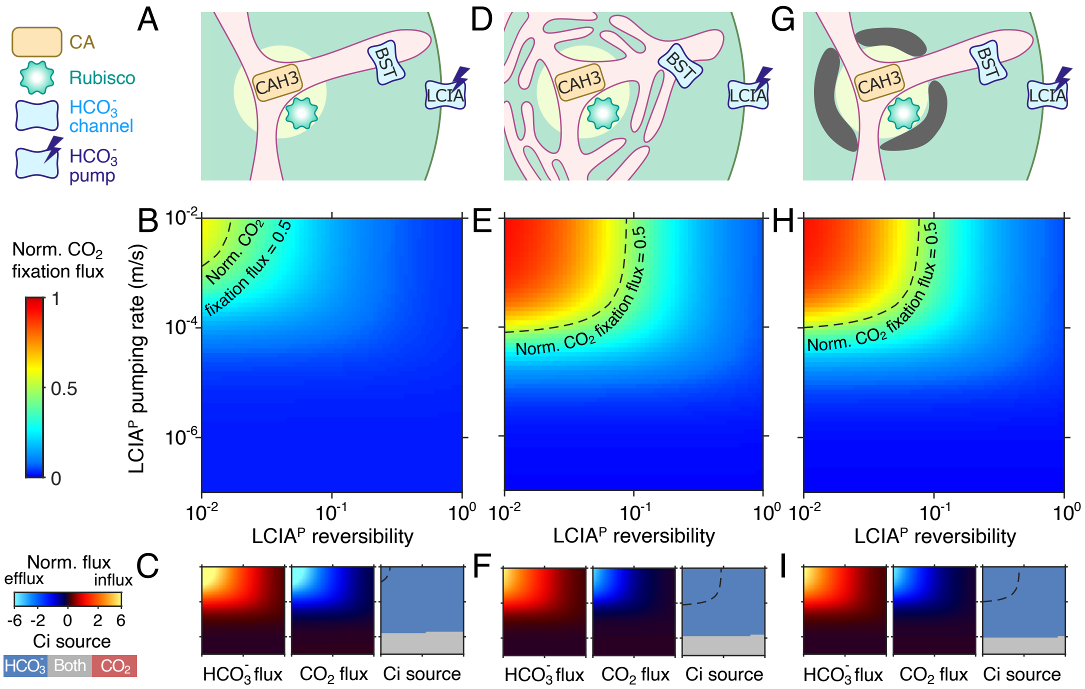

Supplementary Fig. 12. Active HCO_3^- transport across the chloroplast envelope enables an effective PCCM under very low CO_2. A model with no barrier to CO_2 diffusion out of the pyrenoid matrix (*A*–*C*) is compared to a model with thylakoid stacks slowing inorganic carbon diffusion in the stroma (*D*–*F*) and a model with an impermeable starch sheath (*G*–*I*) under very low CO_2 (1 μM cytosolic). (*A*, *D*, and *G*) Schematics of the modeled chloroplast. (*B*, *E*, and *H*) Heatmaps of normalized CO_2 fixation flux at varying $LCIA^P$ pumping rates and reversibilities. The BST-mediated thylakoid membrane permeability to HCO_3^- is the same as in Figs. 1C and D. For *E* and *H*, dashed black curves indicate a normalized CO_2 fixation flux of 0.5. (*C*, *F*, and *I*) Overall fluxes of HCO_3^- (*Left subpanels*) and CO_2 (*Middle subpanels*) into the chloroplast, normalized by the maximum CO_2 fixation flux if Rubisco were saturated, at varying $LCIA^P$ pumping rates and reversibilities. Negative values denote efflux out of the chloroplast. The inorganic carbon (Ci) species with a positive influx is defined as the Ci source, shown in *Right subpanels*. Axes are the same as *B*, *E*, and *H*.

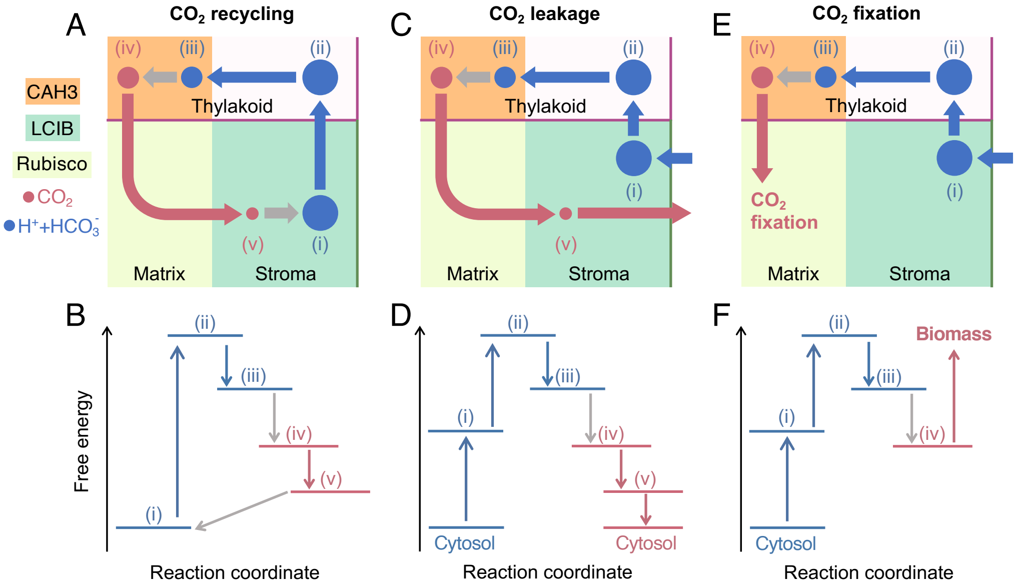

Supplementary Fig. 13. Illustrations of the nonequilibrium processes that cost free energy. Energetic calculation Eq. S46 yields a decomposition of the nonequilibrium fluxes into three processes: (*A, B*) CO_2 recycling, (*C, D*) CO_2 leakage, and (*E, F*) CO_2 fixation (see Sec. II B for details). (*A*, *C*, *E*) Schematics of carbon fluxes. Color code is the same as Fig. 4 except that gray denotes interconversion fluxes between CO_2 and HCO_3^-. (i) HCO_3^- in the stroma; (ii) HCO_3^- in the thylakoids outside the pyrenoid; (iii) HCO_3^- in the thylakoids inside the pyrenoid; (iv) CO_2 in the thylakoids inside the pyrenoid; and (v) CO_2 in the stroma. (*B*, *D*, *F*) Schematics of the free-energy change between the states indicated in *A*, *C*, and *E*. Upward arrows denote processes coupled to energy input, including proton pumping across the thylakoid membranes (blue, from (i) to (ii)), HCO_3^- pumping across the chloroplast envelope (blue, from cytosol to (i)), and CO_2 fixation and biomass production (red). Downward arrows denote nonequilibrium processes that dissipate free energy, including diffusion/transport of CO_2 (red) and HCO_3^- (blue), and reactions catalyzed by carbonic anhydrases (gray).

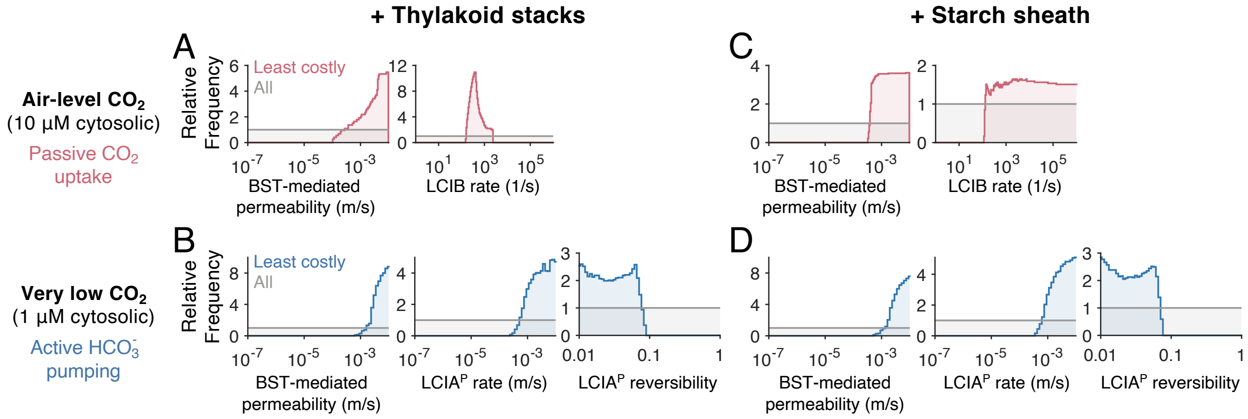

Supplementary Fig. 14. The least energetically costly PCCM strategies in (*A*–*B*) a model with thylakoid stacks slowing the diffusion of inorganic carbon in the stroma and (*C*–*D*) a model with an impermeable starch sheath. The same ensembles of strategies as in Fig. 3 are considered. (*A*,*C*) Relative frequency of (*left*) BST-mediated thylakoid membrane permeability to HCO_3^-, and (*middle*) LCIB rate for the PCCM strategy employing LCIB for passive CO_2 uptake and no HCO_3^- transport across the chloroplast envelope under air-level CO_2 (10 µM cytosolic). (*B*,*D*) Relative frequency of (*left*) BST-mediated thylakoid membrane permeability to HCO_3^-, and (*middle*) the rate and (*right*) reversibility of HCO_3^- transport across the chloroplast envelope by $LCIA^P$, for the CCM strategy employing no LCIB and active HCO_3^- pumping across the chloroplast envelope under very low CO_2 (1 µM cytosolic). In all panels, red or blue denotes parameters underlying the least costly PCCM strategies (Fig. 3 solid curves) that achieve normalized CO_2 fixation flux larger than 0.5. Gray denotes all tested parameters.

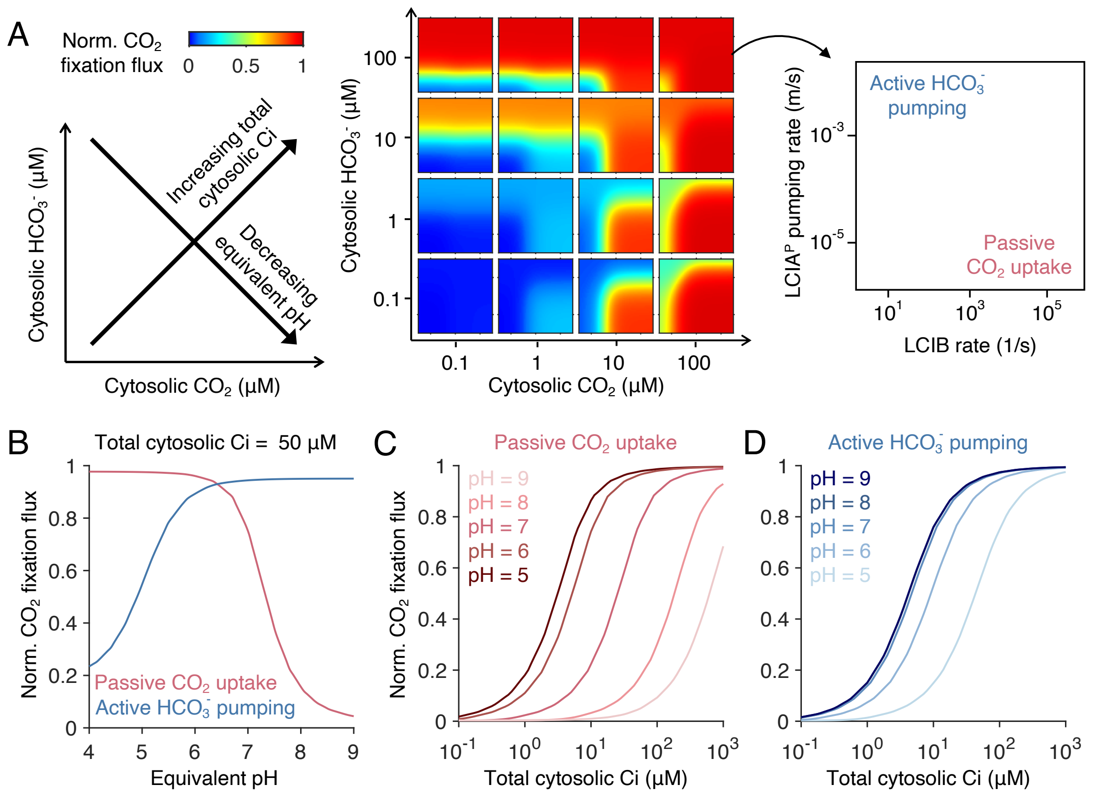

Supplementary Fig. 15. The composition of cytosolic Ci impacts the efficacy of different Ci import strategies. (*A*) Heatmaps of normalized CO_2 fixation flux at varying LCIB rate and $LCIA^P$ pumping rate for different cytosolic CO_2 and HCO_3^- concentrations. *Left:* Schematic of the varying cytosolic Ci compositions. Equivalent pH is defined as the pH value for which the CO_2 to HCO_3^- ratio corresponds to equilibrium. *Right:* Schematic of one heatmap panel, showing regions where the active HCO_3^- import strategy is employed and where the passive CO_2 uptake strategy is employed. (Note that in some cases the activity of LCIA HCO_3^- pumps ($LCIA^P$) may result in a net export of HCO_3^- from the chloroplast; this occurs when the external CO_2 concentration is high, the LCIB rate is high, and the LCIA pump ($LCIA^P$) is fast, since then the HCO_3^- concentration inside the cell becomes much higher than in the cytosol.) (*B*) Normalized CO_2 fixation flux under the two Ci import strategies versus equivalent pH of the cytosol. Total cytosolic Ci fixed at 50 µM. (*C*, *D*) Normalized CO_2 fixation flux plotted against total cytosolic Ci concentrations at the indicated equivalent pH values, using (*C*) passive CO_2 uptake, and (*D*) active HCO_3^- pumping. For all panels, the reversibility of the HCO_3^- pumps ($LCIA^P$) is $\gamma = 10^{-1.5}$.

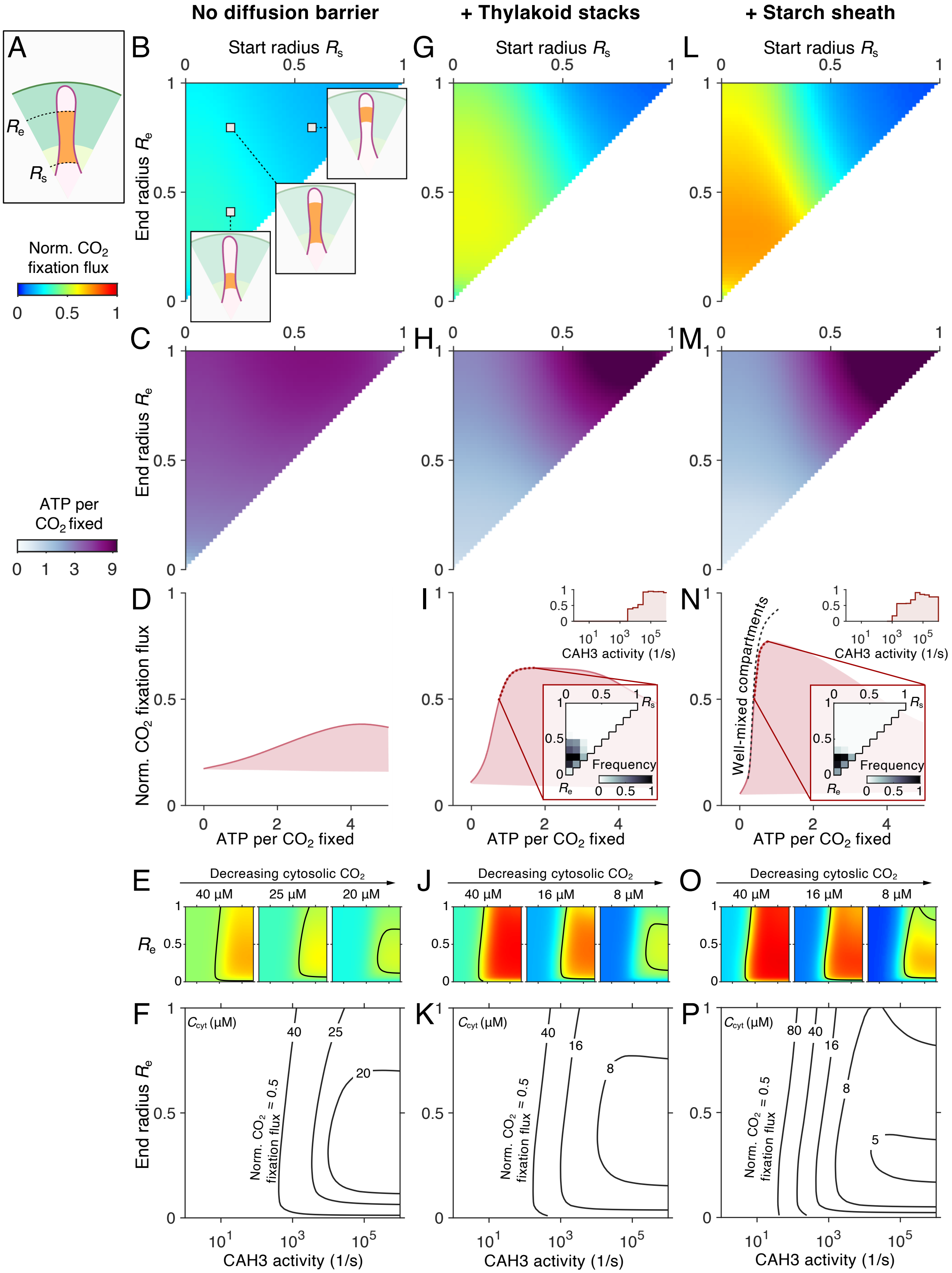

No diffusion barrier
+ Thylakoid stacks
+ Starch sheath
A
B
C
D
E
F
G
H
I
J
K
L
M
N
O
P
Start radius R_s
End radius R_e
Norm. CO_2 fixation flux
ATP per CO_2 fixed
Frequency
CAH3 activity (1/s)
Well-mixed compartments
Decreasing cytosolic CO_2
40 μM
25 μM
20 μM
16 μM
8 μM
C_{cyt} (μM)
Norm. CO_2 fixation flux = 0.5

Supplementary Fig. 16. CAH3 localization and activity that enable an effective PCCM depend on environmental CO_2 levels. (*A*) Schematic of CAH3 (orange) whose localization ranges from start radius R_s to end radius R_e. The varying localization patterns are compared in (*B–F*) a model without diffusion barriers, (*G–K*) a model with thylakoid stacks slowing diffusion of inorganic carbon in the stroma, and (*L–P*) a model with an impermeable starch sheath. (*B*,*G*,*L*) Normalized CO_2 fixation flux (NCFF) and (*C*,*H*,*M*) ATP spent per CO_2 fixed at varying R_s and R_e under air-level CO_2 (10 μM cytosolic). Insets in *B* illustrate the localization patterns for the designated parameter values. CAH3 activity, defined by the first-order rate constant in the baseline model ($R_s = 0$, $R_e = R_{pyr}$), is set to be 10^4 s^{-1}. The total number of CAH3 is held constant for a fixed CAH3 activity. (*D*,*I*,*N*) NCFF versus ATP cost per CO_2 fixed under air-level CO_2 (10 μM cytosolic). Shaded regions show the possible performances by varying CAH3 localization patterns and activities. Solid curves indicate the lowest energy cost for a particular NCFF. Dotted curves indicate the points where NCFF $\geqslant 0.5$, which can be achieved by the activities (*top*) and localization patterns (*bottom*) shown in the insets. Dashed black curve in *N* indicates the optimal PCCM performance of a well-mixed compartment model (Fig. 3 and Sec. IV A). (*E*,*J*,*O*) NCFF at varying activities and end radii of CAH3 under the designated cytosolic CO_2 concentrations. Black curves indicate NCFF = 0.5. (*F*,*K*,*P*) Contours of NCFF = 0.5 under the designated cytosolic CO_2 concentrations. In all panels, the passive CO_2 uptake strategy (Sec. IV B) is considered, i.e., $\kappa_{chlor}^{H^-} = 10^{-8}$ m/s, LCIB first-order rate constant is 10^3 s^{-1}, $\kappa_{thy}^{H^-} = 3 \times 10^{-4}$ m/s.

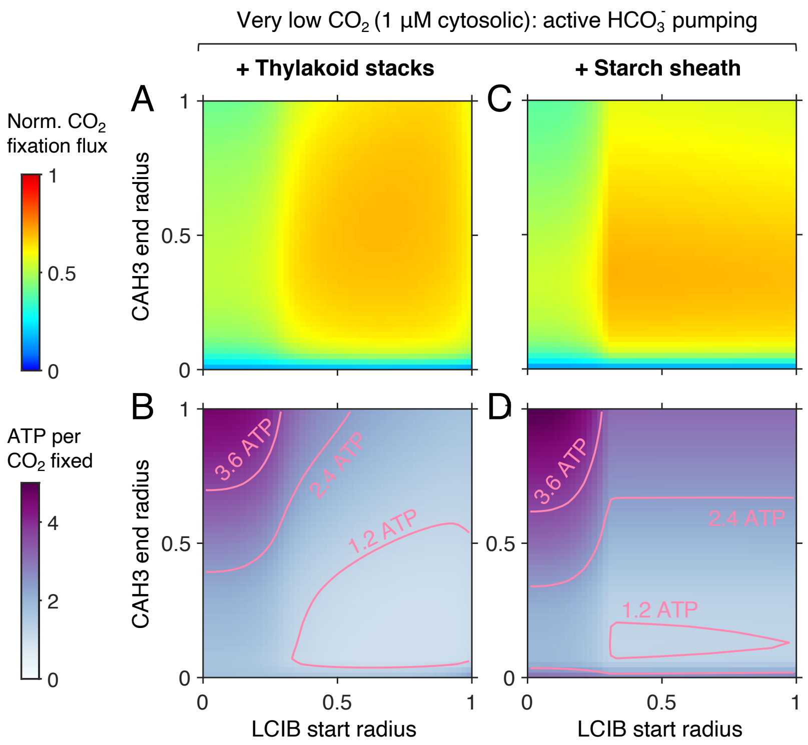

Supplementary Fig. 17. Localization of carbonic anhydrases enhances PCCM performance under very low CO_2. CAH3 end radius and LCIB start radius are varied in a modeled chloroplast employing the active HCO_3^- pumping strategy under very low CO_2, (*A*–*B*) with thylakoid stacks slowing inorganic carbon diffusion in the stroma or (*C*–*D*) with an impermeable starch sheath. (*A*, *C*) Normalized CO_2 fixation flux and (*B*, *D*) ATP spent per CO_2 fixed when the localizations of carbonic anhydrases are varied. Plots correspond to Figs. 4B–E. The rate and reversibility of $\mathrm{LCIA^P}$-mediated HCO_3^- pumping across the chloroplast envelope are $\kappa_{\mathrm{chlor}}^{H^-} = 3 \times 10^{-4}$ m/s and $\gamma = 0.01$, respectively. The parameters are chosen such that CO_2 fixation flux reaches a level similar to that in Fig. 4. Other simulation parameters are the same as in Fig. 4.

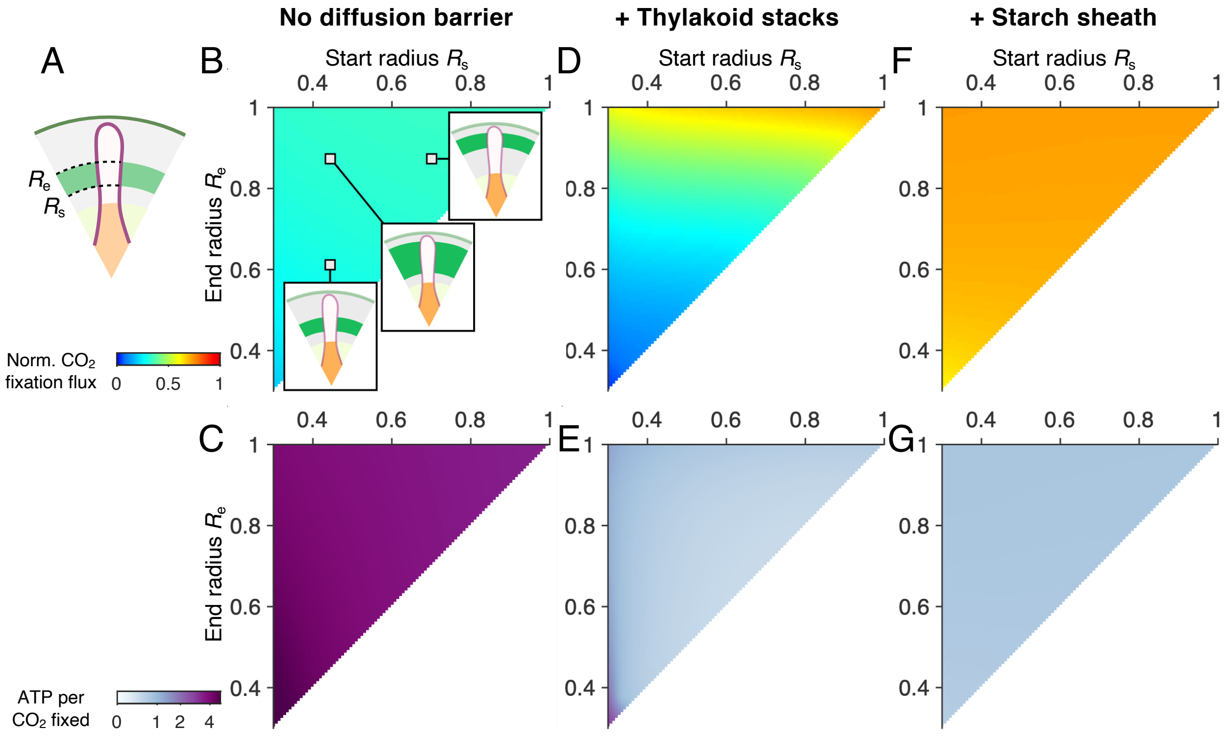

Supplementary Fig. 18. A strong diffusion barrier limits the dependence of PCCM activity on LCIB localization for a passive CO_2 uptake strategy. (*A*) Schematic of LCIB localization (green) ranging from start radius R_s to end radius R_e. Varying localization patterns are compared in (*B*–*C*) a model without diffusion barriers, (*D*–*E*) a model with thylakoid stacks slowing diffusion of inorganic carbon in the stroma, and (*F*–*G*) a model with an impermeable starch sheath. (*B*,*D*,*F*) Normalized CO_2 fixation flux and (*C*,*E*,*G*) ATP spent per CO_2 fixed at varying R_s and R_e under air-level CO_2 (10 μM cytosolic). Insets in *B* illustrate localization patterns for the designated parameter values. LCIB activity, defined by the first-order rate constant in the baseline model ($R_\mathrm{s} = R_\mathrm{pyr}$, $R_\mathrm{e} = R_\mathrm{chlor}$), is $10^3\ \mathrm{s}^{-1}$. The total number of LCIB is held constant for this fixed LCIB activity. In all panels, the CO_2-uptake-dominant PCCM strategy (Sec. IV B) is considered, i.e., $\kappa_\mathrm{chlor}^{H^-} = 10^{-7}$ m/s, and $\kappa_\mathrm{thy}^{H^-} = 3 \times 10^{-4}$ m/s.

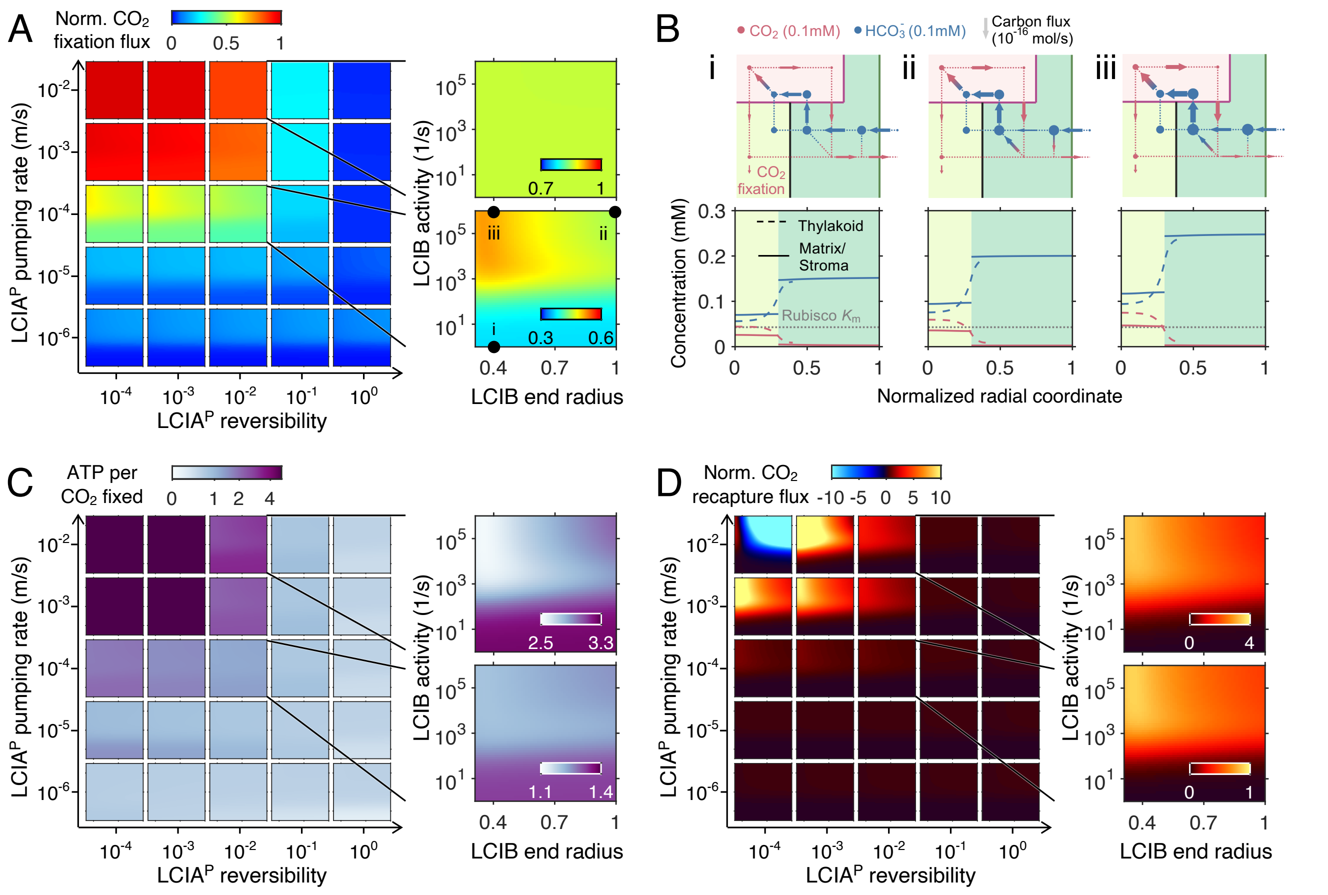

Supplementary Fig. 19. Localization of LCIB around the pyrenoid in a model with a starch sheath allows for CO_2 recycling without inducing carbon leakage from the chloroplast when the active HCO_3^- pumping strategy is employed. (*A*) Heatmaps of normalized CO_2 fixation flux at varying LCIB end radii and LCIB activities are shown for different rates $\kappa_{\mathrm{chlor}}^{H^-}$ and reversibilities γ of LCIA^{P}-mediated HCO_3^- transport across the chloroplast envelope. *Right:* Close-up heatmaps for (*top*) $\kappa_{\mathrm{chlor}}^{H^-} = 10^{-2}$ m/s$, \gamma = 10^{-2}$, and for (*bottom*) $\kappa_{\mathrm{chlor}}^{H^-} = 10^{-4}$ m/s$, \gamma = 10^{-2}$. (*B*) Flux diagrams (*top*) and concentration profiles (*bottom*) of inorganic carbon molecules for the parameters indicated in *A*. Color code is the same as Figs. 1C and D. (*C*, *D*) Same plots as *A* for (*C*) ATP per CO_2 fixed, and (*D*) normalized CO_2 capture flux, defined as the conversion flux of CO_2 to HCO_3^- by LCIB normalized by the maximum CO_2 fixation if Rubisco were saturated. In *D*, a negative value of normalized CO_2 capture flux denotes conversion from HCO_3^- to CO_2. For all panels, parameters are the same as in Fig. 5.

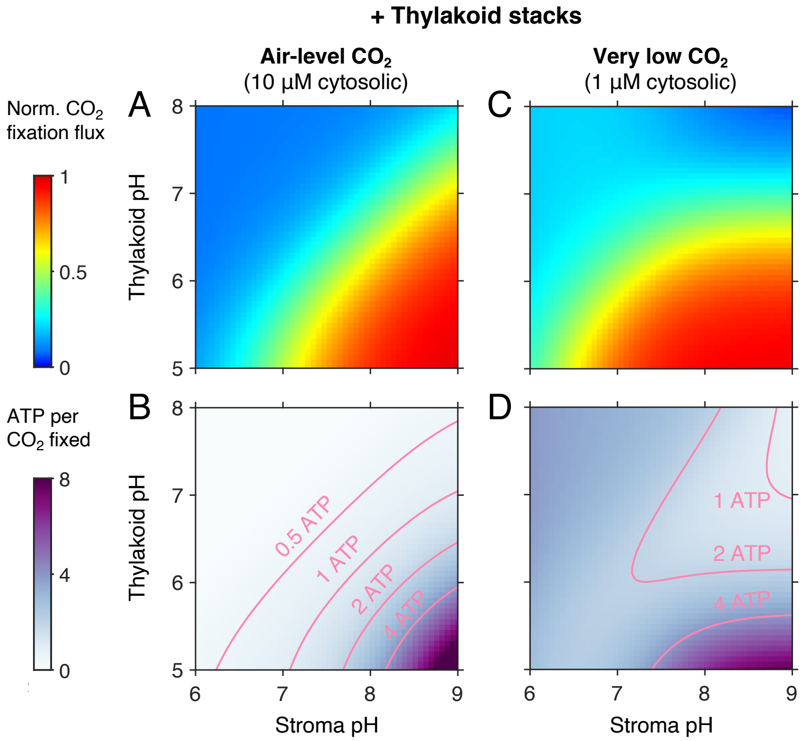

Supplementary Fig. 20. High PCCM performance requires low–pH thylakoids and high–pH stroma. pH values of the thylakoid lumen and the stroma are varied in a modeled chloroplast with thylakoid stacks slowing inorganic carbon diffusion in the stroma: (*A*, *B*) employing the passive CO_2 uptake under air-level CO_2 (10 µM cytosolic), and (*C*, *D*) employing active HCO_3^- pumping under very low CO_2 (1 µM cytosolic). (*A*, *C*) Normalized CO_2 fixation flux and (*B*, *D*) ATP spent per CO_2 fixed as functions of the pH values in the two compartments. Plots correspond to Fig. 6 *A*,*B* and *D*,*E*. Simulation parameters as in Fig. 6.

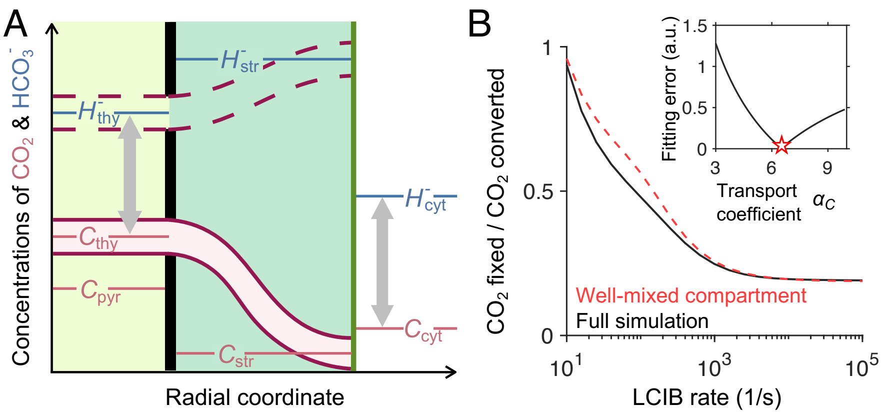

Supplementary Fig. 21. A well-mixed compartment model of the PCCM. (*A*) Schematic of the model. Diffusion is assumed to be fast within each compartment (see Sec. IV for details). Thus, concentrations of CO_2 (red) and HCO_3^- (blue) are represented by horizontal lines, i.e., no gradients. Dashed purple curves indicate that HCO_3^- transport across the thylakoid membrane is fast. Gray arrows indicate species that are in fast equilibrium. Color code is the same as Fig. 1. (*B*) CO_2 fixed per CO_2 converted from HCO_3^- by CAH3 at varying LCIB rates, computed from the full simulation (black solid curve) and from the well-mixed compartment model (red dashed curve). In both calculations, HCO_3^- transport across the chloroplast envelope is negligible. For the full simulation, we set $\kappa_{\text{thy}}^{H^-} = 10^{-2}$ m/s to approximate the fast HCO_3^- transport across the thylakoid membrane. For the well-mixed compartment model, the CO_2 transport coefficient is $\alpha_{\text{thy}}^{C} = 6.5$. *Inset:* Fitting error, defined as the rescaled mean squared difference between the two curves in the main panel when LCIB rate is larger than 10^3 s^{-1}, is plotted against the transport coefficient α_{thy}^{C} (see Sec. IV A). The red star indicates a value of $\alpha_{\text{thy}}^{C} = 6.5$ minimizes the fitting error.

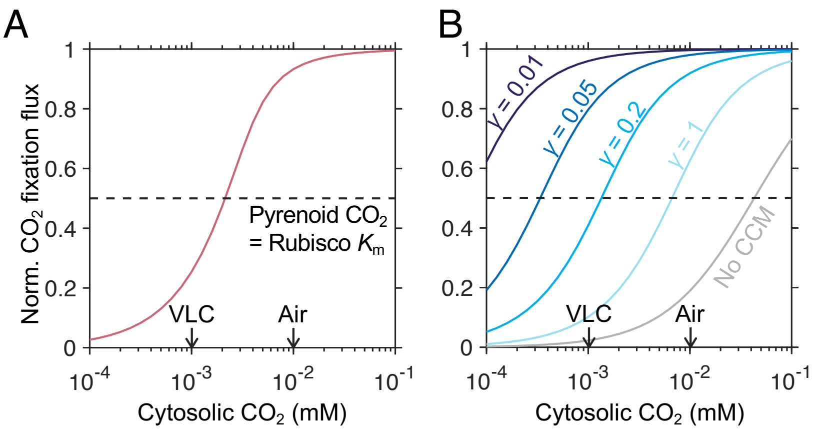

Supplementary Fig. 22. Two extreme CO_2 uptake strategies are compared under various CO_2 conditions for a model employing an impermeable starch sheath and well-mixed compartments. Normalized CO_2 fixation flux under different CO_2 conditions (described by cytosolic CO_2 concentration C_{cyt} in the model) for (*A*) a model that employs fast LCIB for passive CO_2 uptake but no HCO_3^- transport across the chloroplast envelope, and (*B*) a model that employs no LCIB and fast HCO_3^- transport of the designated reversibilties γ across the chloroplast envelope. Black dashed line denotes a normalized CO_2 fixation flux of 0.5. Black arrows indicate air-level CO_2 (Air; 10 μM cytosolic) and very low CO_2 (VLC; 1 μM cytosolic) conditions. In *B*, the gray curve indicates when there is no PCCM, i.e., when the pyrenoid matrix CO_2 concentration is the same as that in the environment.

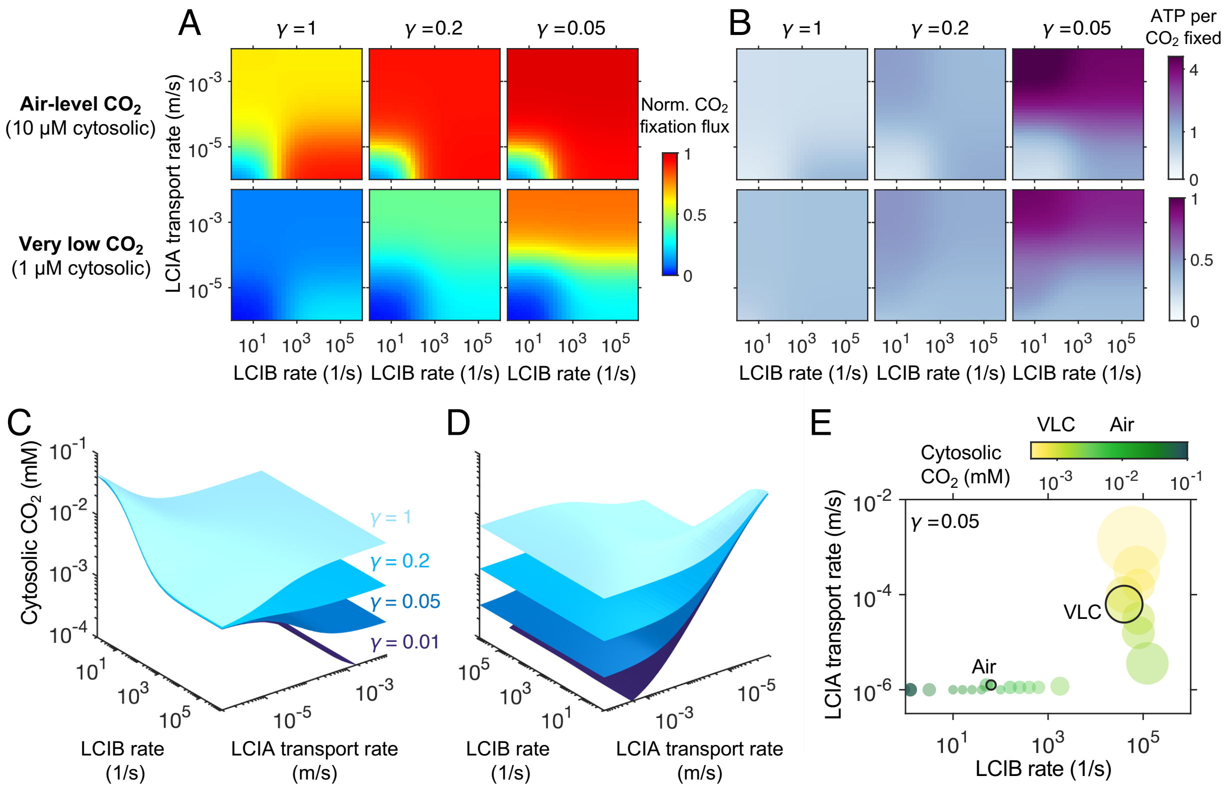

Supplementary Fig. 23. Feasible PCCM strategies depend on external CO_2 levels in the well-mixed compartment model. (*A*) Normalized CO_2 fixation flux and (*B*) ATP spent per CO_2 fixed at varying LCIB rates and LCIA transport rates for the designated LCIA reversibilities γ under air-level CO_2 (top, 10 μM cytosolic) and under very low CO_2 (bottom, 1 μM cytosolic). (*C*) Surfaces of normalized CO_2 fixation flux of 0.5 for the designated values of γ. (*D*) Same as *C* but rotated 180 degrees about the z axis. (*E*) The energetically most efficient PCCM strategies under varying external CO_2 conditions. Circles indicate the regions of parameters in which normalized CO_2 fixation flux is larger than 0.5 and the energetic cost is no more that 5% larger than the minimal cost. The center of each circle is chosen as the regional mean on log scale, and the radius of each circle is proportional to the regional area on log scale. Black outlines denote 10 μM (Air) and 1 μM (VLC) cytosolic CO_2, respectively.

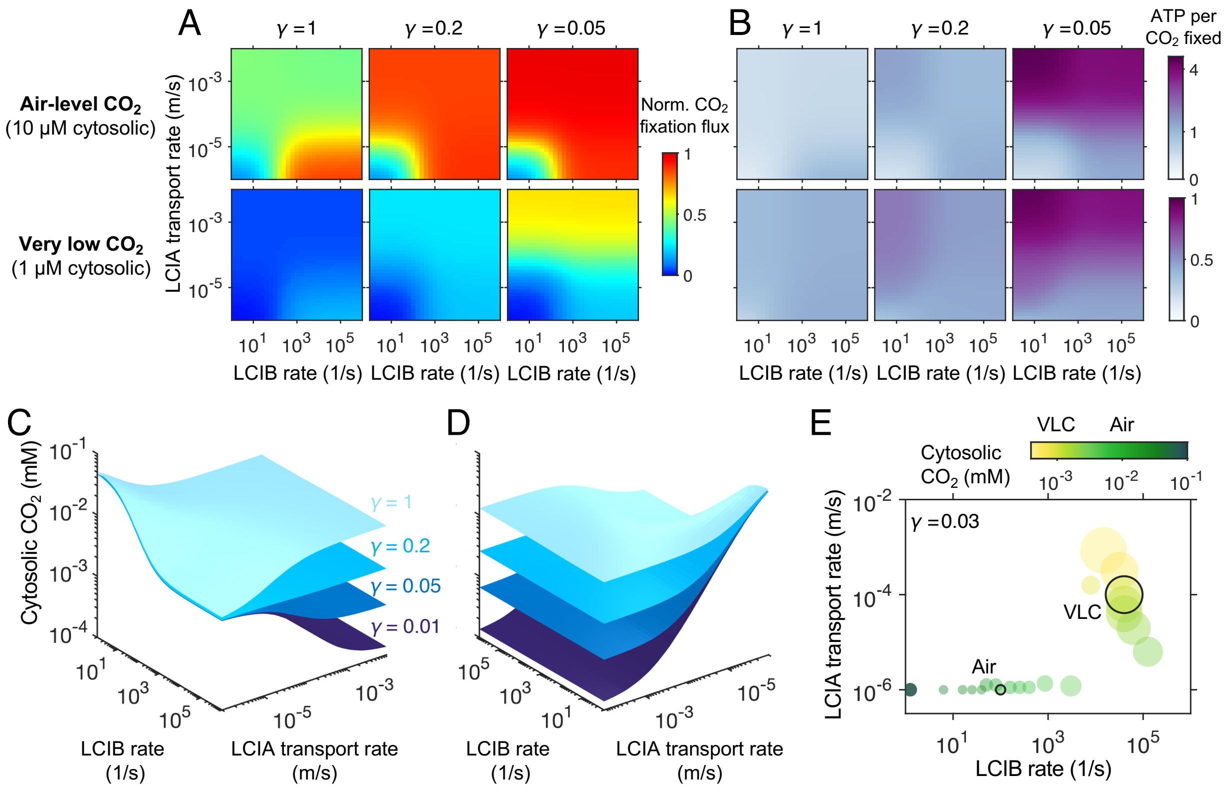

Supplementary Fig. 24. Feasible PCCM strategies depend on external CO_2 levels in the full reaction-diffusion model. (*A*) Normalized CO_2 fixation flux and (*B*) ATP spent per CO_2 fixed at varying LCIB rates and LCIA transport rates for the designated LCIA reversibilities γ under air-level CO_2 (top, 10 μM cytosolic) and under very low CO_2 (bottom, 1 μM cytosolic). (*C*) Surfaces of normalized CO_2 fixation flux of 0.5 for the designated values of γ. (*D*) Same as *C* but rotated 180 degrees about the z axis. (*E*) The energetically most efficient PCCM strategies under varying external CO_2 conditions. Circles indicate the regions of parameters in which normalized CO_2 fixation flux is larger than 0.5 and the energetic cost is no more that 5% larger than the minimal cost. The center of each circle is chosen as the regional mean on log scale, and the radius of each circle is proportional to the regional area on log scale. Black outline denotes 10 μM (Air) and 1 μM (VLC) cytosolic CO_2. All plots shown are for the full model with an impermeable starch sheath around the pyrenoid matrix.

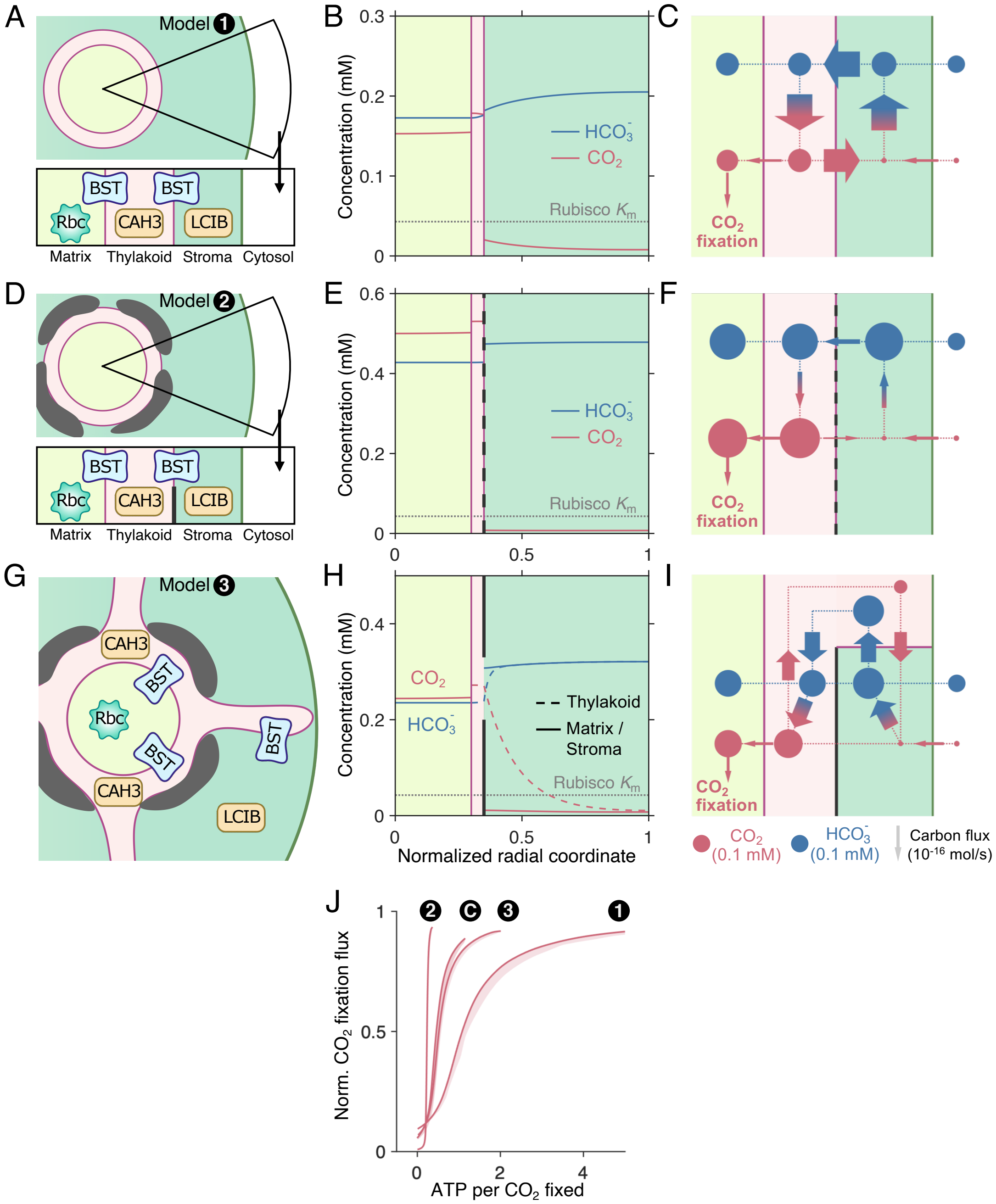

Supplementary Fig. 25. Alternative thylakoid morphologies also support an effective PCCM. We consider a pyrenoid matrix localized at the center of the chloroplast, surrounded by a thylakoid sheet, which is (*A*-*C*) surrounded directly by a stroma (model 1), or (*D*-*F*) surrounded by a gapped starch sheath and a stroma (model 2), or (*G*-*I*) surrounded by a gapped starch sheath and extended by cylindrical tubules into the stroma (model 3). See Sec. VI A for details. (*A*, *D*, *G*) Schematics of the modeled chloroplast. Color code as in Fig. 1A. (*B*, *E*, *H*) Concentration profiles of CO_2 (red) and HCO_3^- (blue) in all compartments. Vertical gray lines denote the starch sheath. Horizontal dotted gray lines indicate the effective K_m of Rubisco (Sec. I B). Color code as in Fig. 1C. (*C*, *F*, *I*) Net fluxes of inorganic carbon between compartments. The width of arrows is proportional to flux; the area of circles is proportional to the average molecular concentration in the compartment. Color code as in Fig. 1D. (*J*) PCCM performance of the passive CO_2 uptake strategy under air-level CO_2 (10 μM cytosolic), measured by normalized CO_2 fixation flux versus ATP spent per CO_2 fixed, is compared among models with Chlamydomonas-like thylakoids (C) and distinct thylakoid morphologies described above (models 1-3). Plots correspond to Fig. 3E. For all panels, parameters are the same as Fig. 1D (Supplementary Table 2, baseline).

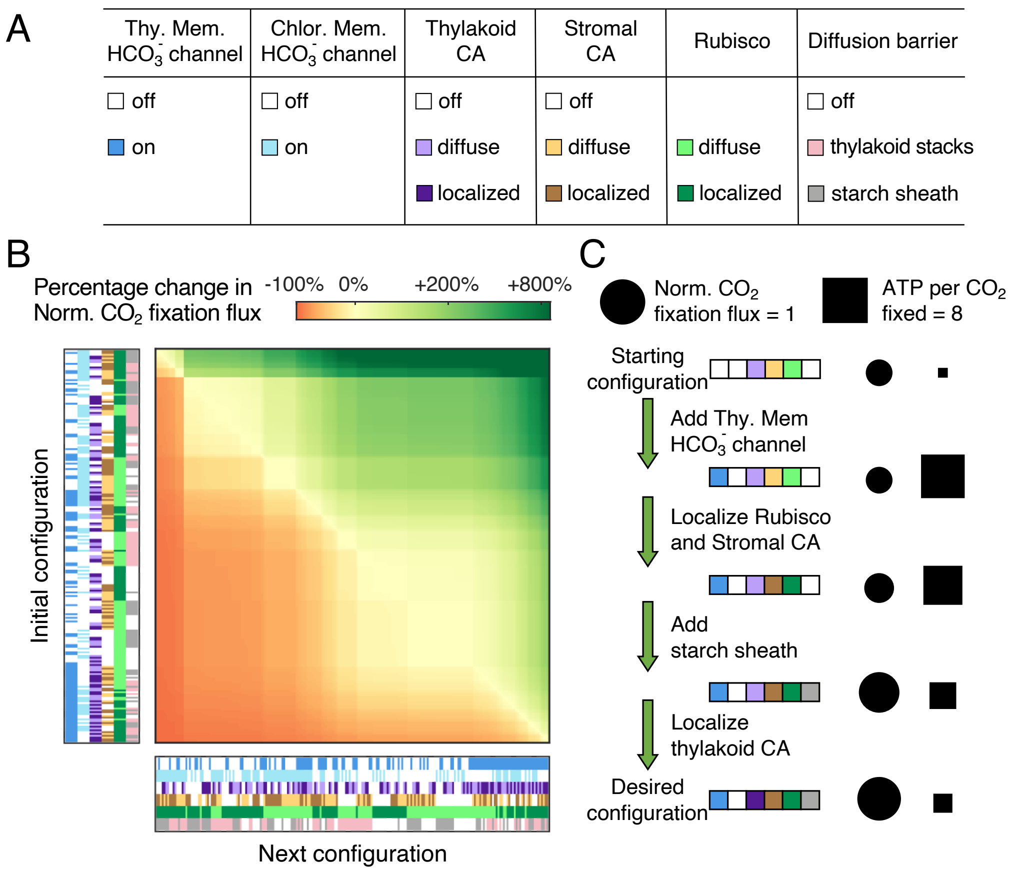

Supplementary Fig. 26. Characterizing various PCCM configurations yields feasible engineering paths for installing a PCCM into plant chloroplasts. (*A*) PCCM components varied in the model. The "off" state denotes no additional permeability to HCO_3^- due to passive channels, rate constant of zero for and carbonic anhydrases (CAs), and $\kappa_{\mathrm{starch}} = 10$ m/s for the starch diffusion barrier (see Sec. I F). The "on" state denotes $\kappa^{H^-}_{\mathrm{thy}} = \kappa^{H^-}_{\mathrm{chlor}} = 10^{-2}$ m/s for the HCO_3^- channels. The thylakoid and stromal CAs, as well as Rubisco, take the baseline activity (Supplementary Table 6), i.e., total number of enzyme molecules, in both the "diffuse" and "localized" states. Note that kinetic parameters for plant Rubisco are used (Supplementary Table 2). The "diffuse" state of the enzymes denotes a start radius $R_s = 0$ and an end radius $R_e = R_{\mathrm{chlor}}$ in their respective compartments (see Sec. I B). The "localized" state of the enzymes denotes the same localization as in the baseline Chlamydomonas model (see Sec. I B). We use the Chlamydomonas thylakoid stacks (Sec. I G), and an impermeable starch at $r = R_{\mathrm{pyr}}$, to model the potential diffusion barriers, regardless of whether Rubisco is localized in a pyrenoid matrix. This yields a combination of 216 CCM configurations. (*B*) Percentage change in the normalized CO_2 fixation flux between every pair of configurations described in *A*. Each configuration is indicated by a six-element color code corresponding to *A*. (*C*) Normalized CO_2 fixation flux (circle, area in proportion to magnitude) and ATP spent per CO_2 fixed (square, area in proportion to magnitude) of all the configurations (indicated by the colored barcode) along one shortest engineering path from the starting configuration representing a typical plant chloroplast to the desired configuration representing a Chlamydomonas chloroplast (see also main Fig. 8). The path above yields the fastest increase in CO_2 fixation flux.

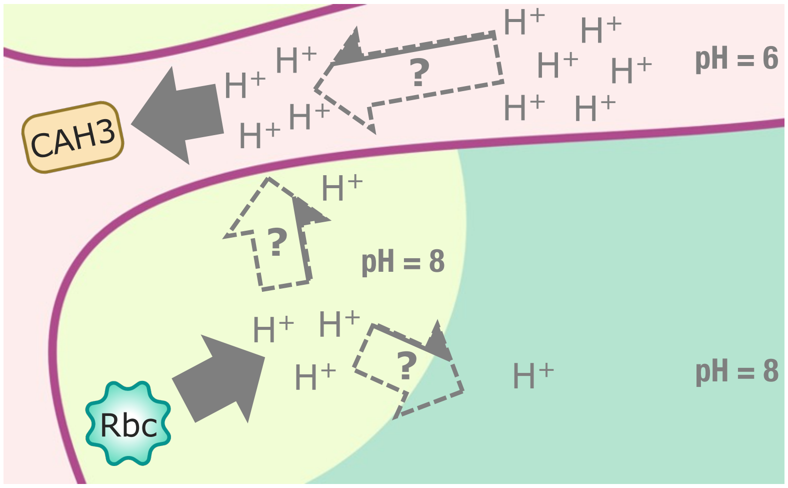

Supplementary Fig. 27. Diffusion fluxes of free protons are not sufficient to maintain compartment pH. Protons are consumed in the acidic lumen of intra-pyrenoid thylakoids via CAH3 and produced in the basic pyrenoid matrix via Rubisco (solid gray arrows). To maintain compartment pH, these protons must be replenished and depleted, respectively (dashed arrows). The diffusion of free protons can only account for a small portion of the fluxes required to maintain constant pH in each compartment (gray portions of dashed arrows). Color code is the same as in Figs. 1C and D.

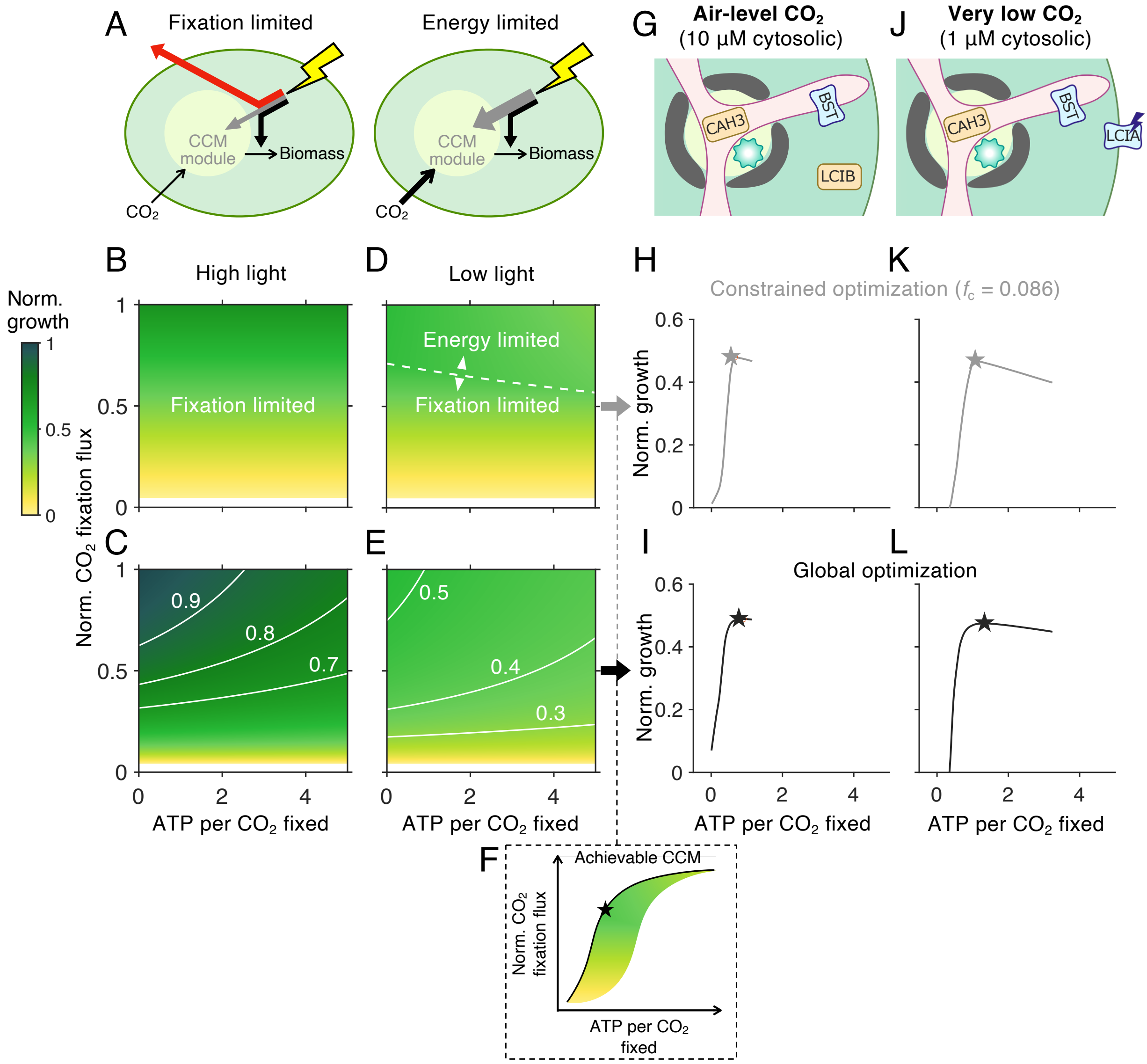

Supplementary Fig. 28. Modeled cell growth rate is determined jointly by the efficacy and energy cost of the PCCM. (*A*) Schematics of two scenarios under which cell growth rate is limited. The total energy input from light (lightning symbol) is either used to concentrate and fix CO_2 (gray), used to convert CO_2 to biomass (black), or dissipated (red). In *left*, the biomass growth rate is limited by CO_2 fixation. In *right*, the biomass growth rate is limited by energy. See Sec. II C for details. (*B* - *E*) Normalized growth rate, defined as the growth rate (Eqs. S48 and S50) compared to its global maximum under saturating light, at varying normalized CO_2 fixation flux and ATP cost of the PCCM under (*B* – *C*) high light (> 200 µmol photons $\cdot$ m^{-2} $\cdot$ s^{-1}), and (*D* – *E*) low light (100 µmol photons $\cdot$ m^{-2} $\cdot$ s^{-1}). *B* and *D* show heatmaps at the estimated PCCM biomass fraction $f_c^0 = 0.086$. White dashed curves indicate the boundary above which growth is energy-limited and below which growth is CO_2 fixation-limited. *C* and *E* show the maximum projection over all values of f_c. (*F*) Schematic of the optimal PCCM strategy (denoted by star) which maximizes the growth rate. Note that the shape of the colored region, which indicates all achievable PCCM strategies, is schematic, and does not represent any data. The optimal PCCM strategies are found by filtering *D* and *E* with the achievable PCCM strategies (denoted by the arrows coming from *D* and *E*) for (*G* – *I*) a modeled chloroplast employing LCIB for passive CO_2 uptake under air-level CO_2, and (*J* – *L*) a modeled chloroplast employing active HCO_3^- pumping across the chloroplast envelope and no LCIB under very low CO_2 (Fig. 3). (*G*, *J*) Schematics of the modeled chloroplast. (*H*, *K*, and *I*, *L*) Normalized growth rates are plotted against ATP spent by the PCCM per CO_2 fixed along the solid curve indicated in *F* for (*H*, *K*) the designated $f_c = 0.086$ (constrained optimization, gray), and for (*I*, *L*) optimal f_c (global optimization, black).

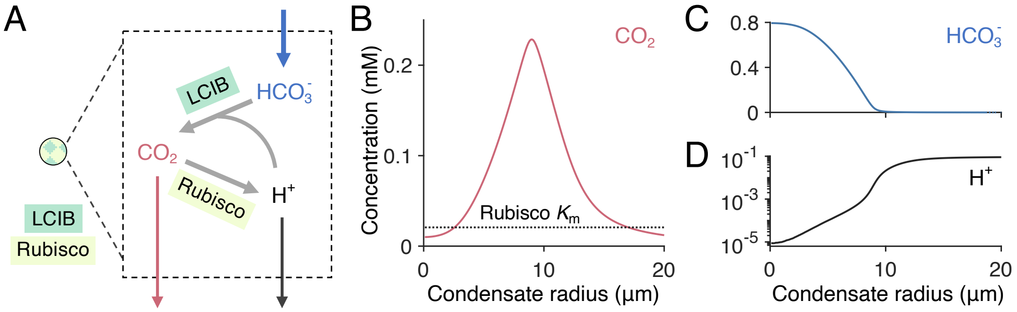

Supplementary Fig. 29. Protons derived from Rubisco CO_2 fixation lead to a notable increase in local proton concentration and CO_2 availability only in 10 µm CA-Rubisco condensates. (*A*) Schematic of the modeled condensate. CO_2 fixation by Rubisco produces protons which in turn drive the conversion of HCO_3^- to CO_2 by LCIB. Color code is the same as in Fig. 1 and Supplementary Fig. 11. Black denotes H^+. (*B*–*D*) Concentrations of (*B*) CO_2, (*C*) HCO_3^-, and (*D*) H^+ in the condensate at varying condensate radius, obtained by solving Eqs. (S78) - (S80). Note that kinetic parameters for plant Rubisco are used (Supplementary Table 2).

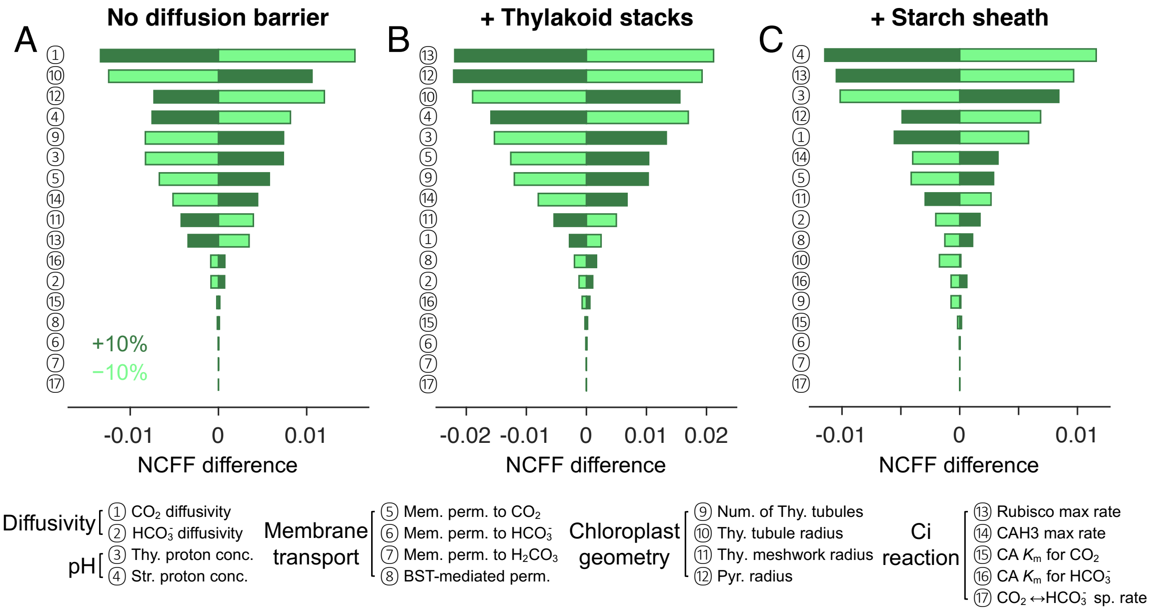

Supplementary Fig. 30. Parameter sensitivity analysis of the multi-compartment reaction-diffusion model. Tornado graphs show the changes in normalized CO_2 fixation flux (NCFF, Sec. II A) when the designated parameters (see Supplementary Table 2) are varied individually by 10% from their baseline values, in (*A*) a model with no diffusion barrier, (*B*) a model with thylakoid stacks slowing inorganic carbon diffusion in the stroma, and (*C*) a model with an impermeable starch sheath. Parameters that impact normalized CO_2 fixation flux the most are shown on the top. Sensitivity analyses are done for the PCCM strategy employing minimal HCO_3^- transport across the chloroplast envelope ($\kappa_{\mathrm{chlor}}^{H^-} = 10^{-8}$ m/s) and intermediate LCIB activity (first-order rate constant 10^3 s^{-1}) under air-level CO_2 (10 μM cytosolic).

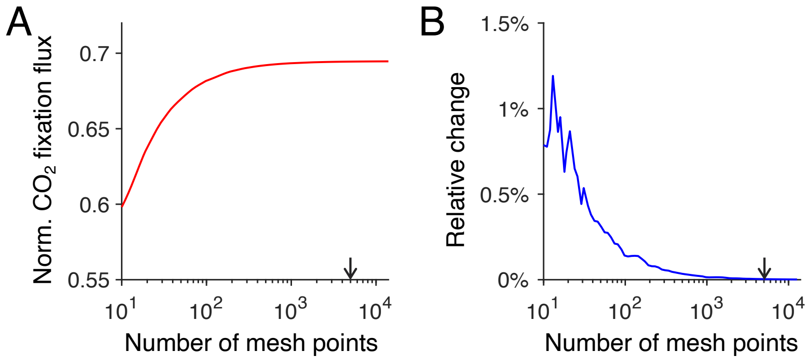

Supplementary Fig. 31. Mesh convergence of the finite-element simulations. The full reaction-diffusion model is simulated for a chloroplast with thylakoid stacks under air-level CO_2 (10 μM cytosolic). Parameters are the same as in Fig. 1D. (*A*) Normalized CO_2 fixation flux, and (*B*) its relative change upon 10% increase of the number of mesh points, plotted against the number of mesh points. Black arrows indicate simulation results with 5000 mesh points, which we use for all other figures.

Supplementary Table 1: Key Chlamydomonas PCCM proteins considered in the model.

Names	Modeled functions	Ref.
LCI1	Plasma membrane CO_2 transporter *	[40]
HLA3	Plasma membrane HCO_3^- transporter (ABC transporter family) *	[34, 73]
LCIA	Chloroplast membrane HCO_3^- transporter (FNT transporter family)	[18, 34]
$LCIA^P$	LCIA modeled as active HCO_3^- pump	
$LCIA^C$	LCIA modeled as passive HCO_3^- channel	
BST	Thylakoid membrane HCO_3^- channel (bestrophin family)	[27]
LCIB	Stromal carbonic anhydrase (β-CA)	[11, 18, 74]
CAH3	Thylakoid carbonic anhydrase (α-CA)	[75, 76]

* Modeled by variation in cytosolic inorganic carbon levels.

Supplementary Table 2: Summary of the notations and parameter values used in the reaction-diffusion model of the PCCM. Subheadings of parameter groups are in bold.

Symbols	Descriptions	Values	Ref.
**Compartments**			
pyr, str, thy	The pyrenoid matrix, stroma, and thylakiod	subscripts	
chlor	The chloroplast	subscripts	
cyt	The cytosol	subscripts	
**Inorganic carbon concentration**			
C, H^-, H^0	Concentrations of CO_2, HCO_3^-, and H_2CO_3		
H	Total concentration of HCO_3^- and H_2CO_3	$H^0 + H^-$	
**pH and pKa**			
$\mathrm{pH_{pyr}}$	pH of the pyrenoid in light	8.0	[2]
$\mathrm{pH_{str}}$	pH of the stroma in light	8.0	[2, 77]
$\mathrm{pH_{thy}}$	pH of the thylakoid lumen in light	6.0	[78]
$\mathrm{pH_{cyt}}$	pH of the cytosol	7.1	[33]
$\mathrm{pKa_1}$	First pKa of H_2CO_3	3.4	[79]
$\mathrm{pK_{eff}}$	pH at which C and H^- are equal	6.1	[80]
η	Equilibrium HCO_3^- to H_2CO_3 concentration ratio	$10^{\mathrm{pH-pKa_1}}$	
K^{eq}	Equilibrium CO_2 to HCO_3^- concentration ratio	$10^{\mathrm{pK_{eff}-pH}}$	
**Chloroplast geometry**			
R_{chlor}	Radius of the chloroplast	3.14 µm	[1, 6]
R_{pyr}	Radius of the pyrenoid	$0.3\,R_{\mathrm{chlor}}$	[1, 6]
N_{tub}	Number of thylakoid tubules	40	[1]
R_{mesh}	Radius of the tubule meshwork	0.4 µm	[1]
a_{tub}	Cylindrical radius of the thylakoid tubules	50 nm	[1]
$f_{\mathrm{v}}(r)$	Volume fraction of the thylakoids at radius r		Sec. I C
$f_{\mathrm{s}}(r)$	Thylakoid surface-to-volume ratio at radius r		Sec. I C
**Diffusion coefficient and permeability**			
D^C	Diffusion coefficient of CO_2 in aqueous solution	1.88×10^3 µm^2/s	[7]
D^{H^-}	Diffusion coefficient of HCO_3^- in aqueous solution	1.15×10^3 µm^2/s	[8]
D_{eff}	Effective diffusion coefficient in the stroma		Sec. I G
κ^C	Membrane permeability to CO_2	300 µm/s	[29]
κ^{H^-}	Membrane permeability to HCO_3^-	0.05 µm/s	[29]
κ^{H^0}	Membrane permeability to H_2CO_3	30 µm/s	[4, 30]
κ_{starch}	Starch sheath permeability to inorganic carbon		Sec. I F
**Enzyme kinetics of carbonic anhydrases**			
$V_{\mathrm{max}}^C/K_{\mathrm{m}}^C$	First-order rate of $CO_2 + H_2O \to HCO_3^- + H^+$	10^4 s^{-1} for CAH3<br>10^3 s^{-1} for LCIB	*<br>*
$K_{\mathrm{m}}^C, K_{\mathrm{m}}^{H^-}$	K_{m} for substrates CO_2 and HCO_3^-	5 mM	[20]
k_{sp}^C	Spontaneous rate of $CO_2 + H_2O \to HCO_3^- + H^+$	0.036 s^{-1}	[81]
**Enzyme kinetics of Chlamydomonas Rubisco**			
$V_{\mathrm{max,Rbc}}^C$	Maximum rate of CO_2 fixation by Rubisco	29 mM/s	[22, 26]
$K_{\mathrm{m,Rbc}}^C$	K_{m} of CO_2 fixation	29 µM	[23]
$K_{\mathrm{m,Rbc}}^O$	K_{m} of oxygenation	480 µM	[23]
O	Dissolved oxygen concentration	230 µM	[24]
$K_{\mathrm{m}}^{\mathrm{eff}}$	Effective K_{m} of CO_2 fixation at $O = 230$ µM	43 µM	Sec. I B
**Enzyme kinetics of plant Rubisco**			
$V_{\mathrm{max,Rbc}}^C$	Maximum rate of CO_2 fixation by Rubisco	19 mM/s	[22, 26]
$K_{\mathrm{m,Rbc}}^C$	K_{m} of CO_2 fixation	14 µM	[23]
$K_{\mathrm{m,Rbc}}^O$	K_{m} of oxygenation	480 µM	[23]
$K_{\mathrm{m}}^{\mathrm{eff}}$	Effective K_{m} of CO_2 fixation at $O = 230$ µM	21 µM	Sec. I B
**Kinetics of HCO_3^- transport**			
$\kappa_{\mathrm{thy}}^{H^-}$	BST-mediated thylakoid membrane permeability to HCO_3^-	10^{-2} m/s	*
$\kappa_{\mathrm{chlor}}^{H^-}$	LCIA^{C}-mediated chloroplast membrane permeability to HCO_3^- or LCIA^{P} HCO_3^- pumping rate	10^{-8} m/s	*
γ	LCIA^{P} reversibility	$0 < \gamma \leqslant 1$	Sec. I E

* Values of unknown parameters in the baseline model. See Fig. 2 and Supplementary Fig. 4 for a thorough parameter scan.

Supplementary Table 3: Summary of the notations and parameter values used in the combined metric calculation (Sec. II C). Subheadings of parameter groups are in bold.

Symbols	Descriptions	Values	Ref.
**Biomass partitioning**			
ν	Number of maintenance photons per biomass	1.67 µmol/g biomass/s	[47]
χ_C	Fraction of carbon in biomass	0.04 mol C/g biomass	[50]
B_{tot}	Total cellular protein biomass	20 pg	[57]
B_{c}	Total PCCM protein biomass	1.76 pg	[57]
f_{c}^0	Fraction of biomass in the PCCM	0.086	[57]
f_{c}^*	Fraction of biomass in the PCCM, excess light, CO_2	0.12	Sec. II C
**Chlamydomonas characteristics**			
N_{Chl}	Amount of chlorophyll per cell	1.5 pg	[56]
a_{Chl}^*	Absorption coefficient of chlorophyll	10 m^2/(g Chl)	[56]
μ	Turnover number per PCCM biomass	27 µmol/g biomass/s	Sec. II C
**Fluxes**			
Γ_C	Flux of CO_2 fixation by Rubisco	$\mu B_{\mathrm{c}} \Phi_C$	
Γ_O	Flux of CO_2 lost to oxygenation	$\mu B_{\mathrm{c}} \Phi_O$	
Γ_{b}	Flux of carbon biomass produced		Sec. II C
Γ_{ph}	Photons absorbed per unit time		
α	Proportionality constant, depends on light intensity	$\frac{\Gamma_{\mathrm{ph}}}{B_{\mathrm{tot}} - B_{\mathrm{c}}}$	
Γ_{m}	Absorbed photons used for cellular maintenance	νB_{tot}	
Φ_C	Normalized CO_2 fixation flux		Sec. II A
Φ_O	Normalized CO_2 loss from oxygenation		Sec. II A
Ψ_C	Normalized net CO_2 fixation flux	$\Phi_C - \Phi_O$	Sec. II A
**Energetic costs and yields**			
ϵ_{b}	Energetic cost of biomass synthesis from CO_2	20 ATP	[48]
ϵ_O	Energetic cost of oxygenation per CO_2 lost	26.4 ATP	[4, 49]
ϵ_{ph}	Yield of chemical energy per absorbed photon	1 ATP	Sec. II C
ϵ_C	Energetic cost of the PCCM		Sec. II B
P_{tot}	Total energy input to the cell	$\epsilon_{\mathrm{ph}}(\Gamma_{\mathrm{ph}} - \Gamma_{\mathrm{m}})$	Sec. II C
**Variables under saturating light**			
$\Gamma_{c,\mathrm{max}}$	Flux of CO_2 fixation at saturating light and CO_2	10 fmol C/cell/s	[16, 26]
$\Gamma_{\mathrm{ph,S}}$	Photons absorbed under saturating light	3 pmol photon/cell/s	Sec. II C
α_S	Proportionality constant at saturating light	76 µmol/g biomass/s	Sec. II C
I_S	Saturating light intensity	200 µmol photon/m^2/s	[55]

Supplementary Table 4: Summary of the parameters and their values used in the well-mixed compartment model. Subheadings of parameter groups are in bold.

Symbols	Descriptions	Values
**Starch sheath model (Sec. IV A)**		
H_i, H_o	HCO_3^- concentrations inside and outside the pyrenoid	
L_{thy}	Diffusion length scale in the thylakoid tubules	≈ 0.95 µm (Supplementary Fig. 21)
ΔS_{thy}	Area of thylakoid membranes in the pyrenoid	8.49 µm^2
$\alpha_{\text{thy}}^{H^-}$	$\frac{2D^H a_{\text{tub}} N_{\text{tub}}}{I[0,R_{\text{pyr}},1,1-f_{\text{v}}]V_{\text{max,Rbc}}^C}$	48.2 mM^{-1}
$I[0, R_{\text{pyr}}, 1, 1-f_{\text{v}}]$	Pyrenoid matrix volume	3.29 µm^3
$I[R_{\text{pyr}}, R_{\text{chlor}}, 1, 1-f_{\text{v}}]$	Stroma volume	125.5 µm^3
α_{thy}^C	$\frac{D^C(\pi a_{\text{tub}}^2/L_{\text{thy}})N_{\text{tub}}}{I[0,R_{\text{pyr}},1,1-f_{\text{v}}]V_{\text{max,Rbc}}^C}$	6.5 mM^{-1} (Supplementary Fig. 21)
σ_{thy}^C	$\frac{\Delta S_{\text{thy}}\kappa^C}{I[0,R_{\text{pyr}},1,1-f_{\text{v}}]V_{\text{max,Rbc}}^C}$	26.7 mM^{-1}
σ_{chl}^C	$\frac{4\pi R_{\text{chlor}}^2\kappa^C}{I[0,R_{\text{pyr}},1,1-f_{\text{v}}]V_{\text{max,Rbc}}^C}$	389.5 mM^{-1}
$\sigma_{\text{chl}}^{H^-}$	$\frac{4\pi R_{\text{chlor}}^2\kappa_{\text{chlor}}^{H^-}}{I[0,R_{\text{pyr}},1,1-f_{\text{v}}]V_{\text{max,Rbc}}^C}$	
β_{LCIB}	$\frac{I[R_{\text{pyr}},R_{\text{chlor}},1,1-f_{\text{v}}]V_{\text{max,LCIB}}^C/K_{\text{m}}^C}{I[0,R_{\text{pyr}},1,1-f_{\text{v}}]V_{\text{max,Rbc}}^C}$	
**Thylakoid stacks model (Sec. V B)**		
β_{CAH3}	$\frac{I[0,R_{\text{pyr}},1,f_{\text{v}}]V_{\text{max,CAH3}}^C/K_{\text{m}}^C}{I[0,R_{\text{pyr}},1,1-f_{\text{v}}]V_{\text{max,Rbc}}^C}$	22.2 mM^{-1}
L_{str}	diffusion length scale in the stroma	Eq. (S63)
α_{str}^C	$\frac{4\pi R_{\text{pyr}}^2(1-f_{\text{v}}\vert_{r=R_{\text{pyr}}})/L_{\text{str}}}{I[0,R_{\text{pyr}},1,1-f_{\text{v}}]V_{\text{max,Rbc}}^C}$	

REFERENCES

[1] Engel, B. D. *et al.* Native architecture of the *Chlamydomonas* chloroplast revealed by in situ cryo-electron tomography. *eLife* **4**, e04889 (2015).

[2] Freeman Rosenzweig, E. S. *Dynamics and liquid-like behavior of the pyrenoid of the green algae Chlamydomonas reinhardtii.* Ph.D. thesis, Stanford University (2017).

[3] Antal, T. K., Kovalenko, I. B., Rubin, A. B. & Tyystjärvi, E. Photosynthesis-related quantities for education and modeling. *Photosynth. Res.* **117**, 1–30 (2013).

[4] Mangan, N. M., Flamholz, A., Hood, R. D., Milo, R. & Savage, D. F. pH determines the energetic efficiency of the cyanobacterial CO_2 concentrating mechanism. *Proc. Natl. Acad. Sci. U.S.A.* **113**, E5354–E5362 (2016).

[5] Gibbons, B. H. & Edsall, J. T. Rate of hydration of carbon dioxide and dehydration of carbonic acid at 25 degrees. *J. Biol. Chem.* **238**, 3502–3507 (1963).

[6] Weiss, D. *et al.* Computed tomography of cryogenic biological specimens based on X-ray microscopic images. *Ultramicroscopy* **84**, 185–197 (2000).

[7] Mazarei, A. F. & Sandall, O. C. Diffusion coefficients for helium, hydrogen, and carbon dioxide in water at 25 °C. *AIChE J.* **26**, 154–157 (1980).

[8] Walker, N., Smith, F. & Cathers, I. Bicarbonate assimilation by fresh-water charophytes and higher plants: I. Membrane transport of bicarbonate ions is not proven. *J. Membr. Biol.* **57**, 51–58 (1980).

[9] Leaist, D. & Lyons, P. Diffusion in dilute aqueous acetic acid solutions at 25 °C. *J. Solution Chem.* **13**, 77–85 (1984).

[10] Karlsson, J. *et al.* A novel α-type carbonic anhydrase associated with the thylakoid membrane in *Chlamydomonas reinhardtii* is required for growth at ambient CO_2. *EMBO J.* **17**, 1208–1216 (1998).

[11] Wang, Y. & Spalding, M. H. An inorganic carbon transport system responsible for acclimation specific to air levels of CO_2 in *Chlamydomonas reinhardtii. Proc. Natl. Acad. Sci. U.S.A.* **103**, 10110–10115 (2006).

[12] Hanson, D. T., Franklin, L. A., Samuelsson, G. & Badger, M. R. The *Chlamydomonas reinhardtii cia3* mutant lacking a thylakoid lumen-localized carbonic anhydrase is limited by CO_2 supply to Rubisco and not photosystem II function in vivo. *Plant Physiol.* **132**, 2267–2275 (2003).

[13] Duanmu, D., Wang, Y. & Spalding, M. H. Thylakoid lumen carbonic anhydrase (*CAH3*) mutation suppresses air-dier phenotype of *LCIB* mutant in *Chlamydomonas reinhardtii. Plant Physiol.* **149**, 929–937 (2009).

[14] Nelson, D. L., Lehninger, A. L. & Cox, M. M. *Lehninger principles of biochemistry* (Macmillan, 2008).

[15] Borkhsenious, O. N., Mason, C. B. & Moroney, J. V. The intracellular localization of ribulose-1,5-bisphosphate carboxylase/oxygenase in *Chlamydomonas reinhardtii. Plant Physiol.* **116**, 1585–1591 (1998).

[16] Mackinder, L. C. *et al.* A repeat protein links Rubisco to form the eukaryotic carbon-concentrating organelle. *Proc. Natl. Acad. Sci. U.S.A* **113**, 5958–5963 (2016).

[17] Blanco-Rivero, A., Shutova, T., Román, M. J., Villarejo, A. & Martinez, F. Phosphorylation controls the localization and activation of the lumenal carbonic anhydrase in *Chlamydomonas reinhardtii. PLoS One* **7** (2012).

[18] Wang, Y. & Spalding, M. H. Acclimation to very low CO_2: contribution of limiting CO_2 inducible proteins, LCIB and LCIA, to inorganic carbon uptake in *Chlamydomonas reinhardtii. Plant Physiol.* **166**, 2040–2050 (2014).

[19] Magid, E. The dehydration kinetics of human erythrocytic carbonic anhydrases B and C. *BBA Enzymol.* **151**, 236–244 (1968).

[20] Bar-Even, A. *et al.* The moderately efficient enzyme: evolutionary and physicochemical trends shaping enzyme parameters. *Biochemistry* **50**, 4402–4410 (2011).

[21] Johnson, K. S. Carbon dioxide hydration and dehydration kinetics in seawater. *Limnol. Oceanogr.* **27**, 849–855 (1982).

[22] Tcherkez, G. G., Farquhar, G. D. & Andrews, T. J. Despite slow catalysis and confused substrate specificity, all ribulose bisphosphate carboxylases may be nearly perfectly optimized. *Proc. Natl. Acad. Sci. U.S.A.* **103**, 7246–7251 (2006).

[23] Jordan, D. B. & Ogren, W. L. Species variation in the specificity of ribulose biphosphate carboxylase/oxygenase. *Nature* **291**, 513–515 (1981).

[24] Kihara, S., Hartzler, D. A. & Savikhin, S. Oxygen concentration inside a functioning photosynthetic cell. *Biophys. J.* **106**, 1882–1889 (2014).

[25] Evans, J. R. & Seemann, J. R. Differences between wheat genotypes in specific activity of ribulose-1,5-bisphosphate carboxylase and the relationship to photosynthesis. *Plant Physiol.* **74**, 759–765 (1984).

[26] Freeman Rosenzweig, E. S. *et al.* The eukaryotic CO_2-concentrating organelle is liquid-like and exhibits dynamic reorganization. *Cell* **171**, 148–162 (2017).

[27] Mukherjee, A. *et al.* Thylakoid localized bestrophin-like proteins are essential for the CO_2 concentrating mechanism of *Chlamydomonas reinhardtii. Proc. Natl. Acad. Sci. U.S.A.* **116**, 16915–16920 (2019).

[28] Lau, C. S. *Characterising the missing thylakoid bicarbonate transport component in Chlamydomonas reinhardtii CO_2 concentrating mechanism.* Master's thesis, York University (2019).

[29] Hopkinson, B. M., Dupont, C. L., Allen, A. E. & Morel, F. M. Efficiency of the CO_2-concentrating mechanism of diatoms. *Proc. Natl. Acad. Sci. U.S.A.* **108**, 3830–3837 (2011).

[30] Xiang, T.-X. & Anderson, B. The relationship between permeant size and permeability in lipid bilayer membranes. *J. Membr. Biol.* **140**, 111–122 (1994).

[31] Bichet, D., Grabe, M., Jan, Y. N. & Jan, L. Y. Electrostatic interactions in the channel cavity as an important determinant of potassium channel selectivity. *Proc. Natl. Acad. Sci. U.S.A.* **103**, 14355–14360 (2006).

[32] Noskov, S. Y., Berneche, S. & Roux, B. Control of ion selectivity in potassium channels by electrostatic and dynamic properties of carbonyl ligands. *Nature* **431**, 830–834 (2004).

[33] Braun, F.-J. & Hegemann, P. Direct measurement of cytosolic calcium and pH in living *Chlamydomonas reinhardtii* cells. *Eur. J. Cell Biol.* **78**, 199–208 (1999).

[34] Yamano, T., Sato, E., Iguchi, H., Fukuda, Y. & Fukuzawa, H. Characterization of cooperative bicarbonate uptake into chloroplast stroma in the green alga *Chlamydomonas reinhardtii. Proc. Natl. Acad. Sci. U.S.A.* **112**, 7315–7320 (2015).

[35] Ramazanov, Z. *et al.* The induction of the CO_2-concentrating mechanism is correlated with the formation of the starch sheath around the pyrenoid of *Chlamydomonas reinhardtii. Planta* **195**, 210–216 (1994).

[36] Villarejo, A., Martinez, F., del Pino Plumed, M. & Ramazanov, Z. The induction of the CO_2 concentrating mechanism in a starch-less mutant of *Chlamydomonas reinhardtii. Physiol. Plant* **98**, 798–802 (1996).

[37] Toyokawa, C., Yamano, T. & Fukuzawa, H. Pyrenoid starch sheath is required for LCIB localization and the CO_2-concentrating mechanism in green algae. *Plant Physiol.* **182**, 1883–1893 (2020).

[38] Wietrzynski, W. *et al.* Charting the native architecture of *Chlamydomonas* thylakoid membranes with single-molecule precision. *eLife* **9**, e53740 (2020).

[39] Van, K. & Spalding, M. H. Periplasmic carbonic anhydrase structural gene (*Cah1*) mutant in *Chlamydomonas reinhardtii. Plant Physiol.* **120**, 757–764 (1999).

[40] Kono, A. & Spalding, M. H. LCI1, a *Chlamydomonas reinhardtii* plasma membrane protein, functions in active CO_2 uptake under low CO_2. *Plant J.* **102**, 1127–1141 (2020).

[41] Buchanan, B. B., Gruissem, W. & Jones, R. L. *Biochemistry and molecular biology of plants* (John Wiley & Sons, 2015).

[42] Beard, D. A. & Qian, H. *Chemical biophysics: quantitative analysis of cellular systems* (Cambridge University Press, 2008).

[43] Qian, H. Phosphorylation energy hypothesis: open chemical systems and their biological functions. *Annu. Rev. Phys. Chem.* **58**, 113–142 (2007).

[44] Mehta, P. & Schwab, D. J. Energetic costs of cellular computation. *Proc. Natl. Acad. Sci. U.S.A.* **109**, 17978–17982 (2012).

[45] Cao, Y., Wang, H., Ouyang, Q. & Tu, Y. The free-energy cost of accurate biochemical oscillations. *Nat. Phys.* **11**, 772 (2015).

[46] Fei, C., Cao, Y., Ouyang, Q. & Tu, Y. Design principles for enhancing phase sensitivity and suppressing phase fluctuations simultaneously in biochemical oscillatory systems. *Nat. Commun.* **9**, 1434 (2018).

[47] Kliphuis, A. M. *et al.* Metabolic modeling of *Chlamydomonas reinhardtii*: energy requirements for photoautotrophic growth and maintenance. *J. Appl. Phycol.* **24**, 253–266 (2012).

[48] Heijnen, J. & Van Dijken, J. In search of a thermodynamic description of biomass yields for the chemotrophic growth of microorganisms. *Biotechnol. Bioeng.* **39**, 833–858 (1992).

[49] Peterhansel, C. *et al.* Photorespiration. *Arabidopsis Book* **8** (2010).

[50] Boyle, N. R. & Morgan, J. A. Flux balance analysis of primary metabolism in *Chlamydomonas reinhardtii. BMC Syst. Biol.* **3**, 4 (2009).

[51] Milo, R. What governs the reaction center excitation wavelength of photosystems I and II? *Photosynth. Res.* **101**, 59–67 (2009).

[52] Yamori, W. & Shikanai, T. Physiological functions of cyclic electron transport around photosystem I in sustaining photosynthesis and plant growth. *Annu. Rev. Plant Biol.* **67**, 81–106 (2016).

[53] Amthor, J. S. From sunlight to phytomass: on the potential efficiency of converting solar radiation to phyto-energy. *New Phytol.* **188**, 939–959 (2010).

[54] Avenson, T. J., Cruz, J. A., Kanazawa, A. & Kramer, D. M. Regulating the proton budget of higher plant photosynthesis. *Proc. Natl. Acad. Sci. U.S.A.* **102**, 9709–9713 (2005).

[55] Leverenz, J. W., Falk, S., Pilström, C.-M. & Samuelsson, G. The effects of photoinhibition on the photosynthetic light-response curve of green plant cells (*Chlamydomonas reinhardtii*). *Planta* **182**, 161–168 (1990).

[56] Langner, U., Jakob, T., Stehfest, K. & Wilhelm, C. An energy balance from absorbed photons to new biomass for *Chlamydomonas reinhardtii* and *Chlamydomonas acidophila* under neutral and extremely acidic growth conditions. *Plant Cell Environ.* **32**, 250–258 (2009).

[57] Hammel, A., Zimmer, D., Sommer, F., Mühlhaus, T. & Schroda, M. Absolute quantification of major photosynthetic protein complexes in *Chlamydomonas reinhardtii* using quantification concatamers (QconCATs). *Front. Plant Sci.* **9**, 1265 (2018).

[58] Mackinder, L. C. *et al.* A spatial interactome reveals the protein organization of the algal CO_2-concentrating mechanism. *Cell* **171**, 133–147 (2017).

[59] Yamano, T. *et al.* Light and low-CO_2-dependent LCIB–LCIC complex localization in the chloroplast supports the carbon-

concentrating mechanism in *Chlamydomonas reinhardtii*. *Plant Cell Physiol.* **51**, 1453–1468 (2010).

[60] Yamano, T., Asada, A., Sato, E. & Fukuzawa, H. Isolation and characterization of mutants defective in the localization of LCIB, an essential factor for the carbon-concentrating mechanism in *Chlamydomonas reinhardtii*. *Photosynth. Res.* **121**, 193–200 (2014).

[61] Zarnitsyn, V., Rostad, C. A. & Prausnitz, M. R. Modeling transmembrane transport through cell membrane wounds created by acoustic cavitation. *Biophys. J.* **95**, 4124–4138 (2008).

[62] Meyer, M. T., Whittaker, C. & Griffiths, H. The algal pyrenoid: key unanswered questions. *J. Exp. Bot.* **68**, 3739–3749 (2017).

[63] Vance, P. & Spalding, M. H. Growth, photosynthesis, and gene expression in *Chlamydomonas* over a range of CO_2 concentrations and CO_2/O_2 ratios: CO_2 regulates multiple acclimation states. *Can. J. Bot.* **83**, 796–809 (2005).

[64] Weissberg, H. L. Effective diffusion coefficient in porous media. *J. Appl. Phys.* **34**, 2636–2639 (1963).

[65] Agmon, N. The Grotthuss mechanism. *Chem. Phys. Lett.* **244**, 456–462 (1995).

[66] Rochaix, J.-D. Regulation and dynamics of the light-harvesting system. *Annu. Rev. Plant Biol.* **65**, 287–309 (2014).

[67] Allen, J. F. Cyclic, pseudocyclic and noncyclic photophosphorylation: new links in the chain. *Trends Plant Sci.* **8**, 15–19 (2003).

[68] Berg, H. C. & Purcell, E. M. Physics of chemoreception. *Biophys. J.* **20**, 193–219 (1977).

[69] Takizawa, K., Cruz, J. A., Kanazawa, A. & Kramer, D. M. The thylakoid proton motive force *in vivo*. Quantitative, non-invasive probes, energetics, and regulatory consequences of light-induced *pmf*. *BBA-Bioenergetics* **1767**, 1233–1244 (2007).

[70] Long, B. M., Förster, B., Pulsford, S. B., Price, G. D. & Badger, M. R. Rubisco proton production can drive the elevation of CO_2 within condensates and carboxysomes. bioRxiv 2020.07.08.125609 (2021).

[71] Anderson, L. E. & Carol, A. A. Enzyme co-localization with Rubisco in pea leaf chloroplasts. *Photosynth. Res.* **82**, 49–58 (2004).

[72] Jebanathirajah, J. A. & Coleman, J. R. Association of carbonic anhydrase with a Calvin cycle enzyme complex in *Nicotiana tabacum*. *Planta* **204**, 177–182 (1998).

[73] Duanmu, D., Miller, A. R., Horken, K. M., Weeks, D. P. & Spalding, M. H. Knockdown of limiting-CO_2–induced gene *HLA3* decreases HCO_3^- transport and photosynthetic Ci affinity in *Chlamydomonas reinhardtii*. *Proc. Natl. Acad. Sci. U.S.A.* **106**, 5990–5995 (2009).

[74] Jin, S. *et al.* Structural insights into the LCIB protein family reveals a new group of β-carbonic anhydrases. *Proc. Natl. Acad. Sci. U.S.A.* **113**, 14716–14721 (2016).

[75] Markelova, A., Sinetova, M., Kupriyanova, E. & Pronina, N. Distribution and functional role of carbonic anhydrase CAH3 associated with thylakoid membranes in the chloroplast and pyrenoid of *Chlamydomonas reinhardtii*. *Russ. J. Plant Physiol.* **56**, 761–768 (2009).

[76] Sinetova, M. A., Kupriyanova, E. V., Markelova, A. G., Allakhverdiev, S. I. & Pronina, N. A. Identification and functional role of the carbonic anhydrase CAH3 in thylakoid membranes of pyrenoid of *Chlamydomonas reinhardtii*. *BBA-Bioenergetics* **1817**, 1248–1255 (2012).

[77] Heldt, H. W., Werdan, K., Milovancev, M. & Geller, G. Alkalization of the chloroplast stroma caused by light-dependent proton flux into the thylakoid space. *BBA-Bioenergetics* **314**, 224–241 (1973).

[78] Kramer, D. M., Sacksteder, C. A. & Cruz, J. A. How acidic is the lumen? *Photosynth. Res.* **60**, 151–163 (1999).

[79] Adamczyk, K., Prémont-Schwarz, M., Pines, D., Pines, E. & Nibbering, E. T. Real-time observation of carbonic acid formation in aqueous solution. *Science* **326**, 1690–1694 (2009).

[80] Millero, F. J. Thermodynamics of the carbon dioxide system in the oceans. *Geochim. Cosmochim. Acta* **59**, 661–677 (1995).

[81] Kern, D. M. The hydration of carbon dioxide. *J. Chem. Educ.* **37**, 14 (1960).
